# Complex Evaluation of Antioxidant Capacity of Milk Thistle Dietary Supplements

**DOI:** 10.3390/antiox8080317

**Published:** 2019-08-18

**Authors:** Jitka Viktorova, Milena Stranska-Zachariasova, Marie Fenclova, Libor Vitek, Jana Hajslova, Vladimir Kren, Tomas Ruml

**Affiliations:** 1 Department of Biochemistry and Microbiology, University of Chemistry and Technology Prague, Technická 5, 166 28 Prague, Czech Republic; 2 Department of Food Analysis and Nutrition, University of Chemistry and Technology Prague, Technická 5, 166 28 Prague, Czech Republice; 3 4th Department of Internal Medicine and Institute of Medical Biochemistry and Laboratory Diagnostics, 1st Faculty of Medicine, Charles University, Katerinska 32, 12000 Prague, Czech Republic; 4 Laboratory of Biotransformation, Institute of Microbiology, Czech Academy of Sciences, Vídeňská 1083, 142 20 Prague, Czech Republice

**Keywords:** antioxidant activity, DPPH, ABTS, ORAC, cellular antioxidant assay, silymarin, milk thistle, U-HPLC-HRMS/MS

## Abstract

Numerous in vitro assays are used to characterize the antioxidant properties of natural-based matrices. However, many of them generate contradictory and non-compliant results. In our study, we focused on the characterization of traditionally used biochemical (2,2′-azino-*bis-*(3-ethylbenzothiazoline-6 sulfonic acid) (ABTS), Oxygen Radical Absorption Capacity (ORAC), and 2,2-diphenyl-1-picrylhydrazyl (DPPH)) and cellular (CAA) antioxidant tests on a broad set of milk thistle dietary supplements containing silymarin. In addition to 26 commercially available preparations, also the natural silymarin extract available from Sigma Aldrich, St. Louis, MI, USA, and a model mixture of pure flavonoid/flavonolignans mimicking the silymarin composition were investigated as control samples. Significant differences in the antioxidant capacity of the supplements were observed. Unlike the DPPH, the results of the ABTS and ORAC methods correlated with the silymarin components determined by U-HPLC-HRMS/MS. The responses in CAA were considerably lower than in other assays. Silymarin exhibited a significantly higher antioxidant capacity than the artificially prepared flavonoid/flavonolignans mixture in all tests, indicating possible presence of other antioxidants of natural origin. The follow-up U-HPLC-HRMS/MS screening revealed the presence of tens of non-silymarin compounds with reported antioxidant activity (not only in the silymarin extract, but also in the milk thistle preparations). The sum of the total phenolics and the sum of the simple phenolics correlated with CAA results more than silymarin.

## 1. Introduction

Antioxidants that compensate for deleterious effects of free radicals on cells and their relations to certain diseases continue to stimulate the research of the antioxidant and antiradical properties of components contained in various natural products and dietary supplements. Among the most important antioxidants and free radicals scavengers are polyphenols, such as phenolic acids and flavonoids.

For the in vitro evaluation of antioxidant activity, a large number of biochemical assays have been developed. Most of them are based on the scavenging of artificial reactive oxygen or nitrogen species. The most frequently used are 2,2′-Azino-*bis*(3-ethylbenzothiazoline-6 sulfonic acid) (ABTS), Oxygen Radical Absorption Capacity (ORAC) [1]. 2,2-Diphenyl-1-picrylhydrazyl (DPPH) assay is a colorimetric, rapid, and sensitive reaction based on the neutralization of a nitrogen radical. 2,2′-Azino-*bis-*(3-ethylbenzothiazoline-6 sulfonic acid) (ABTS) assay is based on the spectrophotometric measurement of specific cation radical neutralization, and in ORAC assay, the oxygen radical absorption capacity is measured kinetically with fluorimetric detection.

However, many of these methods do not exhibit a good correlation with the ability of the compounds to inhibit oxidative deterioration in vivo [2]. This is mainly associated with the fact that the biological manifestation of antioxidant activity depends not only on the chemical reactivity of the antioxidant, but also on its pharmacokinetics. The important factors are bioavailability/bioaccessibility, target location in the organism, related environment, and interaction with other components present. To compensate (at least partially) for these aspects, utilizing cellular assays is usually the preferred option. Nevertheless, in general, due to the complexity of mixtures of antioxidants occurring in complex foods, and different reaction targets, there is no single method capable of accurately describing antioxidant activities, and individual models should be developed for particular food matrices.

In our paper, we assessed the antioxidant properties of silymarin, a complex of bioactive flavonolignans and their flavonoid precursor taxifolin, which are a significant part of the milk thistle (*Silybum marianum* (L.)) plant. The major components of the silymarin complex are silybin A and B, isosilybin A and B, silydianin, silychristin, and isosilychristin, accounting, together with taxifolin, for approximately 70% of the silymarin complex (the remaining part is an undefined yet potentially bioactive polyphenolic fraction) [3,4]. Besides its significant antioxidative potential, the popularity of silymarin is steadily increasing due to its putative chemopreventive and hepatoptotective effects [5,6,7]. However, it should be noted that although most experimental reports and some clinical data suggest that it does play a beneficial role [8,9,10], the clinical importance of silymarin is negligible [5,7,11,12]. Moreover, the results of clinical studies conducted to date are often rather controversial and non-reproducible [7,11,13]. This is probably related to the ambiguously defined chemical composition of the silymarin complex [14] and possible contaminants [15].

The aim of this study was to characterize chemical composition and antioxidation activity of 26 commercial milk thistle-based dietary supplements by a panel of antioxidant activity assays.

## 2. Materials and Methods

### 2.1. Analytical Standards and Chemicals

2,2′-Azo-*bis-*(2-methylpropionamidine) dihydrochloride (AAPH, Sigma-Aldrich, USA); 2,2’-azino-*bis-*(3-ethylbenzothiazoline-6-sulphonic acid) (ABTS, Sigma-Aldrich, USA); 2,2-diphenyl-1-picrylhydrazyl (DPPH, Sigma-Aldrich, USA); 2′,7′-dichlorfluorescein diacetate (DCFH-DA, Sigma-Aldrich, USA); Antibiotic Antimycotic Solution (Sigma-Aldrich, USA); Fetal Bovine Serum (FBS, Sigma-Aldrich, USA); Fluorescein (Sigma-Aldrich, USA); Minimum Essential Medium Eagle (EMEM, Sigma-Aldrich, USA); Potassium persulfate (K_2_S_2_O_8_, Sigma-Aldrich, USA); Quercetin (Sigma-Aldrich, USA); Trolox (Sigma-Aldrich, USA).

Silymarin (defined as’ flavonolignan mixture extracted from the seeds of *Silybum marianum’*, product number S0292, Lot BCBM3466V, declared content of silybin A/B 42.6%, Sigma-Aldrich, USA; further referred to as silymarin SA); Silibinin (mixture of silybin A and B diastereoisomers, product number S0417, Lot BCBP6193V, declared purity 99.1%, Sigma-Aldrich, USA; further referred to as silibinin SA).

The analytical standards of silybin A; silybin B; isosilybin A; isosilybin B; 2,3-dehydrosilybin; silychristin; silydianin; and taxifolin isolated from commercially available silymarin (purchased from Liaoning Senrong Pharmaceutical, Panjin, People’s Republic of China, batch no. 120501) according to a published method (29) were provided by the Laboratory of Biotransformation (Institute of Microbiology of the CAS, Prague, Czech Republic).

The internal reference sample of dried milk thistle extract, containing 139 ± 17 mg/g of silybin A, 179 ± 23 mg/g of silybin B, 38 ± 5.2 mg/g of isosilybin A, 8.8 ± 0.9 mg/g of isosilybin B, 2.5 ± 0.3 mg/g of 2,3-dehydrosilybin, 180 ± 31 mg/g of silychristin, 72 ± 8.3 mg/g of silydianin, and 13.5 ± 6.2 mg/g of taxifolin, was available from our previous study [15]; the reference values were calculated as mean values from repeated analyses (*n* = 40) by the methods described below, obtained over a long period of time.

### 2.2. Samples and Standards Preparation

The milk thistle-based dietary supplement samples investigated in the antioxidation tests were purchased on the Czech and US market between 2016 and 2017, and their characterization as provided by manufacturers, is summarized in Table 1. For each of the supplements, the internal content of twenty capsules was weighed separately and then mixed together to obtain a homogenized representative sample.

For the quantitative analysis of silymarin flavonoid/flavonolignans, the method previously described in Fenclova et al. [15] was followed. Briefly, 1 g of representative sample of the dietary supplement was weighed into a 50 mL PTFE centrifuge tube and 3-times repeatedly extracted by shaking with 15 mL of ethanol, to assure the 100% recovery. The extracts were collected and pooled into a volumetric flask and made up to 50 mL with ethanol. Prior to the analysis, the final extract was diluted 10-, 100-, 1000- and 10,000-fold with ethanol. The analytical standards of silybin A; silybin B; isosilybin A; isosilybin B; 2,3-dehydrosilybin; silychristin; silydianin; and taxifolin were dissolved in ethanol, mixed together and further diluted with ethanol to obtain a set of calibration standards at concentrations of 1, 2.5, 5, 10, 25, 50, 100, 250, 500, 1000, and 2500 ng/mL.

For the purpose of the targeted screening of antioxidant compounds, 0.5 g of the representative sample of the dietary supplement was weighed into 15 mL PTFE centrifuge tube and extracted by shaking with 5 mL of methanol. The silymarin SA was also extracted by the same procedure.

For the purpose of the antioxidant activity determinations, 30 mg of the representative sample of the dietary supplement was weighed into a 50 mL PTFE centrifuge tube and extracted by shaking with 30 mL of methanol. The silymarin SA was dissolved in methanol and further diluted with methanol to obtain a 1 mg/mL solution. To reflect the composition of this solution, the model mixture of analytical standards of silybin A; silybin B; isosilybin A; isosilybin B; 2,3-dehydrosilybin; silychristin; silydianin; and taxifolin in methanol was also prepared. The representation of individual flavonoid / flavonolignans in the silymarin complex determined by the ultra-high performance liquid chromatography high-resolution tandem mass spectrometry (U-HPLC-HRMS/MS), thus in the model mixture, is depicted in Table 2. The silibinin SA purchased from Sigma-Aldrich was dissolved in methanol and further diluted to obtain a set of calibration standards at concentration levels of 10, 25, 50, 100, 250, 500, 750, 1000, 1200, 1750, and 2500 mg/L.

### 2.3. Quantitative Analysis of Silymarin Flavonoid/Flavonolignans by U-HPLC-HRMS/MS

The quantitative analysis of silymarin flavonolignans and flavonoid taxifolin by ultra-high-performance liquid chromatography coupled with high-resolution tandem mass spectrometry (U-HPLC-HRMS/MS) in dietary supplements and the silymarin SA was performed according to [15].

Dionex UltiMate 3000 ultra-high performance liquid chromatograph (Thermo Scientific, Sunnyvale, CA, USA) with a reversed phase Accucore; aQ analytical column (150 mm × 2.1 mm; i.d. 2.6 µm; Thermo Scientific, San Jose, CA, USA) and gradient elution in water—methanol—ammonium formate / formic acid system, and a Q-Exactive^TM^ high resolution tandem mass spectrometer (Thermo Scientific, Bremen, Germany) were used. The following exact masses were considered for detection: *m/z* 303.0510 ([M-H]^−^ions of taxifolin), 479.0984 ([M-H]-ions of 2,3-dehydrosilybin) and 481.1140 ([M-H]^−^ions of the other isomeric flavonolignans). The limits of quantification of silymarin components were estimated as the lowest concentration levels of the calibration batch providing long-term stable signals, and were 0.75, 0.75, 0.5, 0.5, 0.25, 2.5, 1.25, and 1.25 μg/g for silybin A, silybin B, isosilybin A, isosilybin B, 2,3-dehydrosilybin, silychristin, silydianin, and taxifolin, respectively. The reproducibility of the method, expressed as a relative standard deviation (RSD), was assessed by repeated analyses (*n* = 7) of an internal reference sample of milk thistle-based dietary supplement, and was 2.7%, 2.9%, 3.6%, 4.2%, 5.4%, 3.1%, 2.8%, and 3.2% for silybin A; silybin B; isosilybin A; isosilybin B; 2,3-dehydrosilybin; silychristin; silydianin; and taxifolin, respectively.

### 2.4. Antioxidant Activity Determinations

The antioxidant activity of milk thistle-based dietary supplements was studied using several different assays.

#### 2.4.1. ABTS Radical Scavenging Assay

The fresh ABTS^+^ radicals solution was prepared according to [16]. Milk thistle-based dietary supplement extracts were binary diluted in order to determine their individual concentration of a supplement that gives half-maximal response (EC_50_). The tested concentration range was from 0.26 to 66.66 mg/L. The quenching of the ABTS^+^ radicals by the extracts was monitored spectrophotometrically (734 nm), based on the changes in the absorption spectrum of the ABTS radical using the SpectraMax i3x Multi-Mode Detection Platform (Molecular Devices, San Jose, CA, USA). The percentage of inhibition was calculated according to the formula: 100 × (NC absorbance − sample absorbance)/(NC absorbance). The experiment was performed in 3 repetitions.

#### 2.4.2. Oxygen Radical Absorption Capacity (ORAC)

For each experiment, fluorescien freshly diluted with PBS was prepared according to [17]. The binary dilution of the milk thistle-based dietary supplement extracts was done to provide the final tested concentration range of 0.78–200 mg/L. The ability of extracts to quench AAPH radicals was monitored by measuring the fluorescence (excitation/emission 485/535 nm), recording for 2 h at 5 min intervals using the SpectraMax i3x Multi-Mode Detection Platform (Molecular Devices, San Jose, CA, USA). The kinetic parameters were calculated as usual. The relative activity was evaluated as a percentage according to the formula: 100 × (slope of sample fluorescence − average slope of PC)/(average slope of NC − average slope of PC). The experiment was performed in 3 repetitions.

#### 2.4.3. DPPH Radical Scavenging Assay

The DPPH solution was freshly prepared two hours before each measurement according to [18]. The samples were binary diluted in order to determine EC_50_ values. The tested concentration range was from 0.65 to 166.66 mg/L. The quenching of the DPPH-H radicals was recorded using the SpectraMax i3x Multi-Mode Detection Platform (Molecular Devices, USA) as an absorbance difference (at 517 nm). The percentage of inhibition was calculated according to the formula: 100 × (NC absorbance − sample absorbance)/(NC absorbance). The reaction mixture where the sample was replaced with equal amount of solvent (methanol) served as a negative control. The experiment was performed in three independent repetitions.

#### 2.4.4. Cellular Antioxidant Activity (CAA) Assay

The CAA assay was slightly modified according to [19]. Human hepatoblastoma HepG2 cells (ATCC, HB-8065) were seeded at a density of 5 × 10^5^/mL in EMEM medium supplemented with 10% FBS and Antibiotic Antimycotic Solution. After 24 h, the cells were washed with PBS and DCFH-DA (0.0125 mg/mL in medium without FBS) was added. The sample extracts were added to the final concentrations of 0.1–25 mg/L. After 1 h of co-incubation, the medium was replaced with AAPH (44 µM in PBS) and fluorescence (ex./em. 485/540 nm) was immediately recorded at 5min intervals for 1 h using the SpectraMax i3x Multi-Mode Detection Platform (Molecular Devices, USA). The experiment was done in four replicates. The evaluation procedure and controls were the same as in the ORAC assay.

The highest testing concentration of the dietary supplement extracts, 25 mg/L, was chosen intentionally to reflect the real daily dose of overall silymarin when following the producers’ consumption recommendations (i.e., approximately 500 mg, [15]). Taking into account the average volume of human blood of 4 L, the approximately 10% bioavailability of silymarin in the organism [20], and approximately 50% of silymarin in the capsules, we calculated the approximate concentration of 25 mg per liter of a biological fluid.

### 2.5. Targeted Screening of Antioxidants by U-HPLC-HRMS/MS

The targeted screening of non-silymarin antioxidants reported in the literature for all of the plants occurring in the dietary supplements (Table S1) was performed by the U-HPLC-HRMS/MS method using the 1290 Infinity LC system (Agilent Technologies, Sant Clara, CA, USA) coupled with Agilent 6560 QTOF mass spectrometer (Agilent Technologies, USA). The reversed phase Acquity UPLC BEH C18 analytical column (100 mm × 2,1 mm; i.d. 1,7 μm; Waters, USA) was used for the gradient elution, where the mobile phases consisted of H_2_O: MeOH (95:5, v/v) (A) and ^i^-PrOH:MeOH: H_2_O (65:30:5, v/v/v) (B), both containing 5 mM ammonium formate and 0.1% formic acid. The gradient was linear from 100% of A (initial; kept for 1 min) to 100% of B within 14 min, which was held for 5 min and followed by column equilibration for 2 min under the initial conditions. The mobile phase flow rate was 0.35 mL/min and the injection volume 1 µL. The mass spectrometer was operated in Auto MS/MS mode with the following parameters: electrospray ionization in positive and negative mode (ESI^+^ and ESI^−^; separate injections), drying gas (N_2_) temperature 280 °C and flow rate 12 L/min, nebulizer 35 psig, sheath gas (N_2_) temperature 350 °C and flow rate 12 L/min, capillary voltage 3 500 V, nozzle voltage 400 V. The following parameters were used in Auto MS/MS mode: mass range 100–1100 *m/z* (MS) and 50–1100 *m/z* (MS/MS), acquisition rate 3 spectra/s (MS) and 12 spectra/s (MS/MS), collision energy 20 V. The antioxidants were tentatively identified based on exact masses of the particular ions, their isotopic patterns, and the compliance of MS/MS spectra. For some of the compounds, more chromatographic peaks fulfilling the HRMS identity criterions were identified, referring probably to structural isomers of analysed bioactive compounds. The details about the peaks identity, mainly retention times and degrees of certainty of compounds identification, are presented in Table S2. For purposes of correlations of occurrence of these compounds with antioxidant activities (as described in details in the Section 2.6), the peak areas of all isomers were summed up together.

### 2.6. Statistical Analysis and Correlation

The EC_50_ values were obtained using the GraphPad Prism 7 software (GraphPad Software, San Diego, CA, USA). Both standard errors of the mean (SEM) and standard deviations (SDs) were calculated as usual. Data were analysed by one-way analysis of variance (ANOVA) followed by Duncan’s post-hoc test (*p* > 0.05). All computations were done using the statistical software STATISTICA 10. The correlation coefficients were calculated using the automatic function “CORREL” in Microsoft^®^ Office Excel (i.e., “matrix I” and “matrix II” explained below were correlated against each other). The following variables were used as matrix I: (i) concentrations of particular flavonoid/flavonolignans and overall silymarin, (ii) the peak areas of non-silymarin *Silybum marianum* antioxidants identified by targeted U-HPLC-HRMS/MS screening, namely phenolics, flavones, flavone glycosides, isoflavonoids, flavonolignans, and their sums, and (iii) the peak areas of other non-*Silybum marianum* antioxidants identified by targeted U-HPLC-HRMS/MS screening, namely phenolics, coumarins, lignans, lignan glycosides, flavones, isoflavonoids, saponines, terpenes, and their sums. The analytical standards of non-silymarin components were not available, so we correlated the sum of areas of the peaks belonging to the respective chemical class. The results of the antioxidant capacity of 26 dietary supplements obtained from all assays investigated (i.e., (i) ABTS assay, (ii) ORAC assay, (iii) DPPH assay, (iv) CAA assay) were utilized as matrix II. The significance of the correlation coefficient was evaluated using a comparison of coefficients and the critical values (α = 0.05), which were determined using the degrees of freedom (df = *n* − 2).

## 3. Results

### 3.1. Silymarin Content and Composition

We determined the content of silymarin components in order to correlate it with the antioxidant properties of the tested supplements. As shown in Table 3, the main component of the silymarin complex was silybin B, representing 20% (sample 2)—35% (sample 1) of the total amount of silymarin. The exceptions to this rule were samples 2, 12, 13, and 14, where the main component was silychristin, and sample 10, where the dominant component was silybin A. In these samples, the reduced quantity of silybin B constituting only about 20% (sample 2)—26% (sample 10) was replaced by an increased amount of silychristin, representing 24% (sample 14)—28% (sample 2) or silybin A, representing 29% (sample 10). The most effective component of the silymarin complex—taxifolin [21], represented 2% (samples 1, 5, 6, 8, 10, and 16)—6% (sample 14 and 23) of the total amount of silymarin components. The total amount of silymarin varied from 5 (sample 5) to 393 (sample 17) mg/g.

### 3.2. Antioxidant Activity of Milk Thistle-Based Dietary Supplements

**Figure 1:**
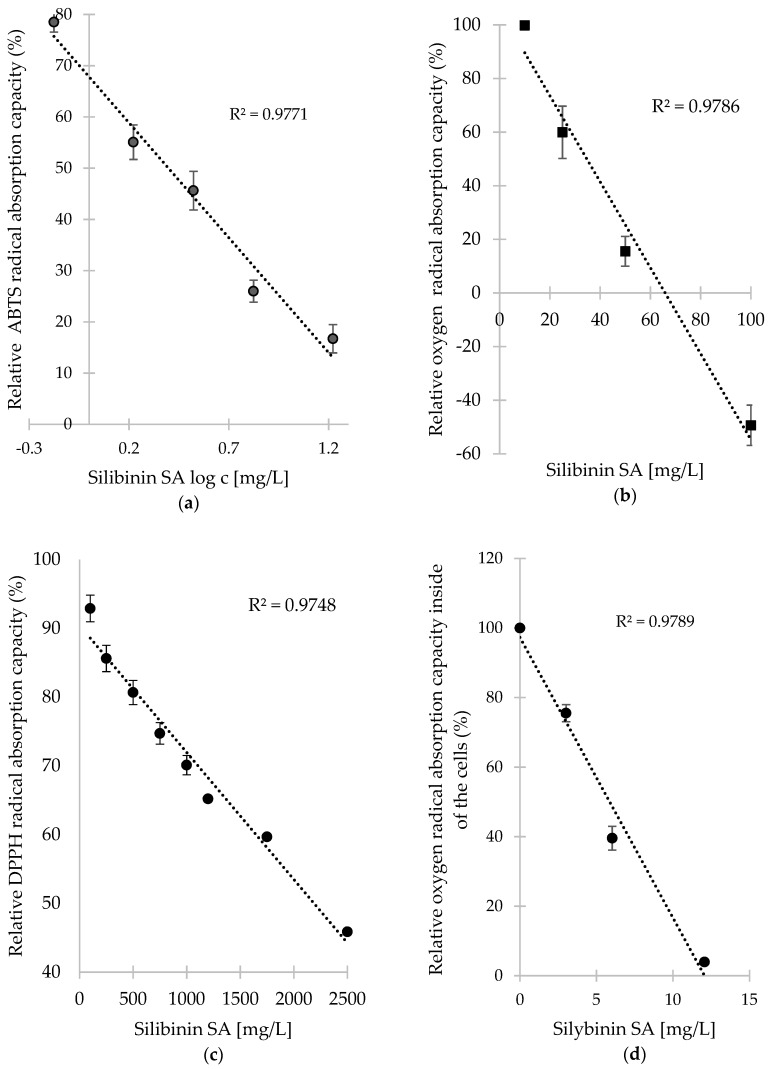
Calibration curve of dependence of silibinin SA content on 2,2′-azino-*bis-*(3-ethylbenzothiazoline-6 sulfonic acid) (ABTS) radical (**a**), oxygen radical—ORAC assay (**b**), 2,2-diphenyl-1-picrylhydrazyl (DPPH) radical (**c**) and oxygen radical inside of cells—CAA assay (**d**) scavenging. Data are presented as an average of three replicates with SDs.

As shown in Figure 1, the linearity ranges tested with the standard of silibinin SA were rather narrow for all the assays investigated. In such cases, the analysis of a big set of samples, where the antioxidant capacity was highly unpredictable, was quite difficult when using a uniform weight of samples. Therefore, to demonstrate the differences between the abilities of particular dietary supplements to act as antioxidant agents, the EC_50_ (effective concentration able to scavenge 50% of free radicals) values were used.

In order to compare the results of antioxidant assays used for evaluating the capacity of milk-thistle dietary supplements to quench different radicals, the correlation of results was performed. The best correlation was observed for the ABTS and CAA assay (R^2^ = 0.463) followed by ABTS and DPPH assays (R^2^ = 0.454); nevertheless, the correlation coefficients values were not high enough to be significant (for both correlations, the df value was equal to 14 and the critical value was equal to 0.497). ORAC and CAA are chemically identical assays, differing only in the cellular environment in the latter one. Their low correlation coefficient (R^2^ = −0.034) was mainly caused by bioavailability, which was taken into the account in the CAA assay. The low correlations were observed for the DPPH assay compared to the ORAC and CAA assays (R^2^ = 0.305, 0.025, respectively) and ORAC and ABTS assay (R^2^ = 0.210).

#### 3.2.1. ABTS Radical Scavenging Activity

The lowest effective concentrations (8.5, 8.7, 9.9, 10.3, and 12.0 mg/L) inhibiting 50% of radicals (EC_50_), i.e., the highest antioxidant properties, were achieved for the samples 1, 3, 18, 19, and 17, respectively. By far the least active was the sample 15 (48.3 mg/L). The samples 4, 5, 6, 10, and 16 failed to inhibit 50% of radicals in the maximum tested concentration of 66.7 mg/L (Table 4). The data obtained by this assay corresponded well to the overall sum of flavonoid/flavonolignans concentrations found in the samples. The highest flavonoid/flavonolignans content was found in the samples 1, 17, 18, and 19, and the lowest in samples 4, 5, 10, and 16, well reflected particular antioxidant capacity (Table 3).

#### 3.2.2. Oxygen Radical Absorption Capacity

In accordance with previous results, the highest antioxidant properties were found for samples 20 and 17 (12.6–13.9 mg/L), followed by sample 19 and 18 (16.2–16.7 mg/L). Again, the EC_50_ value was not determined for samples 4, 5, 10, and 16, because of their low antioxidant ability (Table 4).

#### 3.2.3. DPPH Radical Scavenging Activity

Despite significantly broader linearity range in the DPPH test compared to ABTS and ORAC (see Figure 1), EC_50_ values were also used in this case to facilitate the comparison of the result with the other assays. As shown in Table 4, the maximum concentrations used in the assay (167 mg/L) were unable to scavenge 50% of free radicals in nine of the dietary supplement samples. Besides the samples 4, 5, 10, and 16 that were shown to lack antioxidant activity in previous tests, samples 6, 7, 11, 13, and 15 were also classified as weak antioxidants in the DPPH scavenging assay. Rather surprisingly, the sample 3 exhibited the highest potential to scavenge DPPH radicals, with a low flavonoid/flavonolignans content (Table 3). Similarly to the previous experiments, the sample 17 exhibited a high antioxidant potential.

#### 3.2.4. Cellular Antioxidant Capacity

Human hepatoblastoma cells were used as the model. Their intrinsic antioxidant capacity was measured after co-cultivation with the tested samples. As expected, the samples 4, 5, 10, and 16 were also inactive in the CAA assay (Table 4). Moreover, a low or almost zero antioxidant intracellular capacity was detected for the samples 1, 2, 6, 7, 13, and 25. The most promising milk thistle-based supplement were the samples 3 and 17 (EC_50_ = 11.3 and 12.5 mg/L), which is in agreement with previous biochemical assays.

### 3.3. Comparison of Commercially Available Silymarin SA with Artificially Prepared Mixture of Flavonoid/Flavonolignans Mimicking its Composition

Silymarin is a mixture of flavonolignans of different chemical structures, properties, and antioxidant capacities, therefore the composition of commercially available silymarin SA was characterized in detail. As indicated in Table 2, the main component of the silymarin complex was silybin B, which formed about 26.0% of the silymarin content followed by silybin A (22.1%), and silychristin, which reached 21.5%. Other less abundant components were isosilybin A (13.2%), silydianin (6.2%), isosilybin B (5.1%), and taxifolin (4.9%). Approximately one percent was represented by 2,3-dehydrosilybin. Interestingly, the sum of flavonoid/flavonolignans formed approximately 51% of the purchased silymarin powder. This relatively low total percentage of active components may be associated with the presence of an undefined and non-determined polyphenolic fraction) [3,4], or with co-extracts present after milk thistle fruit (seed) extraction.

**Figure 2:**
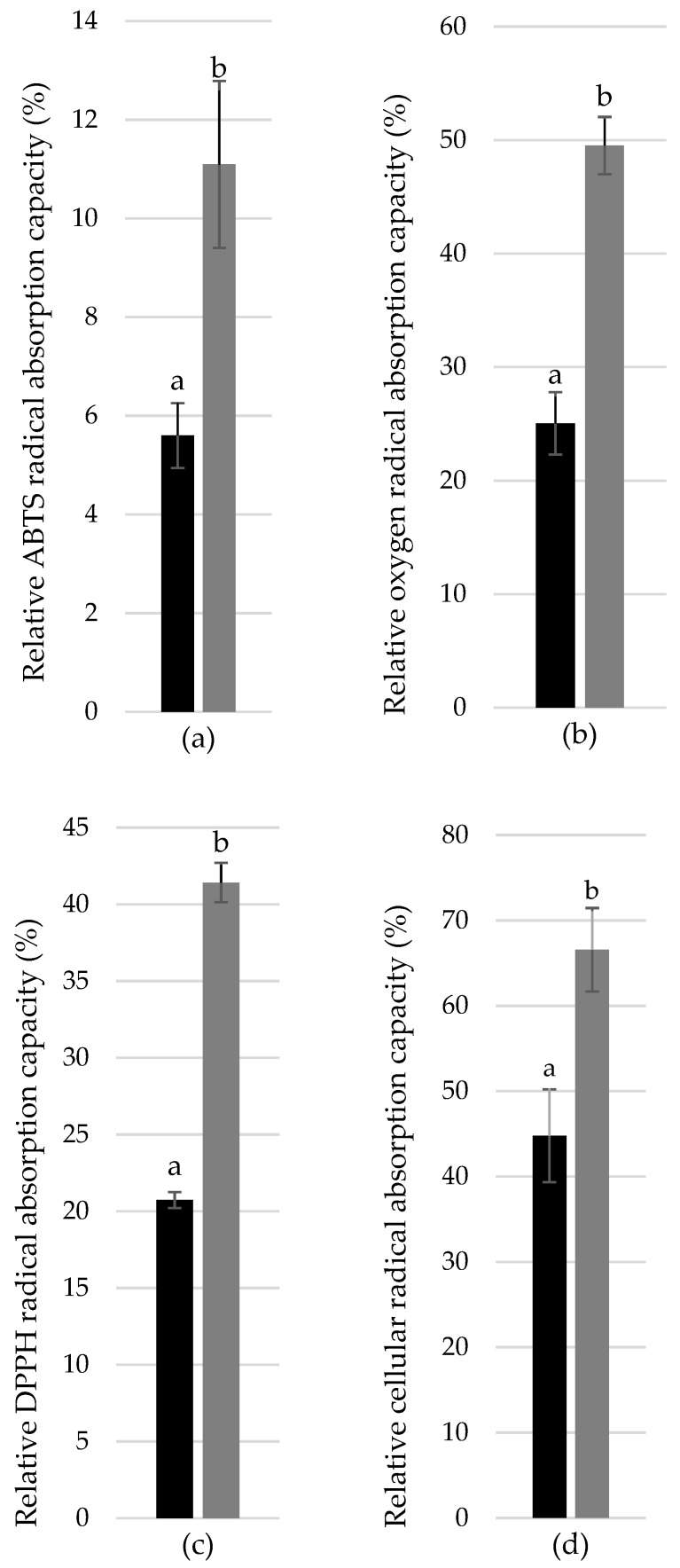
Difference between the antioxidant capacity of commercially available silymarin SA (black columns) and corresponding artificial mixture of flavonolignans (grey columns) determined using ABTS (**a**), ORAC (**b**), DPPH (**c**), and CAA assays (**d**). The lower the value of relative radical quenching activity, the higher antioxidant activity. The same volume of stock solutions [1 g/L] was used in each assay. Data are presented as the average of six replicates with appropriate SEM. Data were analysed by one-way analysis of variance (ANOVA) followed by Duncan’s post-hoc test (*p* > 0.05), the statistical differences are indicated by letters.

As the next step, we prepared the mixture of flavonoid/flavonolignans from chemically pure individuals, precisely mimicking the composition of Silymarin components, and performed the antioxidant activity tests. One-dose measurements were performed in this experiment, comparing commercially available silymarin SA to the artificially prepared flavonoid/flavonolignans mixture. As shown in Figure 2, the antioxidant capacity of silymarin SA was significantly higher than the capacity of the artificial silymarin that mimicked the mixture in all antioxidant assays used in the study (the differences in DPPH, ORAC, and ABTS assay were statistically significant in one-way ANOVA, followed by Duncan’s post hoc test (*p* > 0.05)). Based on this, we could deduce the presence of other, non-silymarin antioxidants, and thus the non-target screening of those compounds was performed. As predicted, many antioxidant substances (*n* = 47) were identified in the silymarin SA preparation (Table S2).

### 3.4. Characterization of Non-Silymarin Antioxidants Occurring in Milk-Thistle-Based Dietary Supplements

The targeted screening of non-silymarin *Silybum marianum* antioxidants was done in order to identify other natural substances responsible for the antioxidation effect. As summarized in Table S2, each milk thistle-based preparation contained at least one additional antioxidant commonly present in *Silybum marianum* plants, mostly the flavonoids (flavonoid glycosides, iso-/flavones, flavanols, flavones, and flavonols) and phenols (polyphenols or simple phenols) were determined. The occurrence of such a large spectrum of different antioxidants partially explains the unexpectedly high responses of some low-silymarin samples in the antioxidant activity tests.

Moreover, as indicated in Table 1, some of the dietary supplements were prepared from other plant species than Silybum marianum, including targeted screening of antioxidants present in plants such as *Schisandra chinensis*, *Cordyceps sinensis*, *Scutellaria baicalensis*, *Cnicus benedictus*, *Foeniculum vulgare*, *Taraxacum officinale,* and *Glycyrrhiza glabra*. As shown in Table S2, 53 structures previously described in the scientific literature were found in the analysed samples.

### 3.5. Correlation of Antioxidant Activity with Particular Antioxidants

To determine the relationship between the antioxidant activity response in the particular test examined, and the amount of the main antioxidants present in the samples, the correlation of the antioxidant capacity results with the concentrations of the antioxidants in each sample was calculated. When plotting the results for all of the 26 samples tested, the correlation coefficient (R^2^) was determined and assessed (see Table 5, Tables S3 and S4). As can be seen in Table 5, the antioxidant activity results determined by ORAC significantly correlated with the concentrations of particular antioxidants (taxifolin, silychristin, silydianin, silybin A/B, isosilibin A/B, and 2,3-dehydrosilybin AB) and overall silymarin (sum of all specific flavonoid/flavonolignans). Similar results were also obtained for the ABTS test, i.e., a significant correlation between the test response and the concentrations of silydianin, silybin A/B, isosilibin A, and overall silymarin. On the other hand, the DPPH method provided very poor correlations with all of the silymarin components concentrations, the only significant correlation was measured for the concentration of taxifolin. As for the cellular CAA assay, the best correlation of the obtained responses and silymarin component concentrations was observed for silydianin, which could be due to its solubility or bioavailability. However, despite the highest value, this correlation was not statistically significant either. Nevertheless, for this type of assay, significant correlation was also observed for other non-silymarin antioxidants of *Silybum marianum* (Table S3), especially for sum of simple phenolics (e.g., dicaffeoylquinic acid; feruloylquinic acid; caffeic acid; coniferylaldehyd; coumaric acid; cynarin; ferulic acid; gallic acid; guaiacol; chlorogenic acid; mariamide A,B; methyl ferulate; salicylic acid; sinapinic acid; syringaldehyde; syringic acid; vanillic acid; and others) and sum of total phenolics (sum of simple phenolics, flavones, flavones glycosides, isoflavonoids, and flavonolignans of non-silymarin origin).

## 4. Discussion

Dietary supplements based on the milk thistle (*Silybum marianum*) are among the most common preparations used by the EU and US adult population [22]; in fact, those are among the six best-selling herbal-based products in the US [23], but unfortunately with an insufficient level of composition control. The increasing popularity of silymarin for the treatment of liver and chemoprevention has generated scientific interest in this topic [6,7,8], and it can be said that despite the suggested beneficial role of silymarin [7,8,9], its clinical importance has still not been clearly proven [2,8,10,11]. The main limitations of the clinical studies conducted so far seem to be the lack of properly controlled clinical trials, especially in terms of the vaguely defined chemical composition of the therapeutic agents, silymarin preparations [14,24].

In our study, where 26 milk thistle-based dietary supplements were investigated, a significant variability in the content of total silymarin was observed, as well as in the composition of the silymarin complex (which is somewhat in line with the results of only two previous studies on this topic [25,26]). Our results showed relatively significant differences in the ratios of the most abundant flavonolignans silybin B, silybin A, and silychristin. Moreover, the content of 2,3-dehydrosilybin and silydianin, a minor flavonolignan possessing potent biological activities [4,27,28,29], differed approximately six- and 60-fold, respectively, across the positive samples (Table 3). It is also important to note that the correspondence of the determined concentrations of silymarin with the producers’ declarations on the packaging is very low, as evidenced in our recent study on an identical set of samples [15].

Because the antioxidant capacity of silymarin preparations, as determined by different analytical methods, reflect not only the chemical composition, but also the mechanisms of antiradical reactions, we compared the four most frequently used antioxidant activity assays (ABTS, ORAC, DPPH, and CAA), and evaluated the relationships between their results and the composition of complex samples. Silymarin has been previously tested for its radical scavenging ability in several separate studies [11,19,20]. Even the chemical assays for antioxidant evaluation can be considered simple, rapid, sensitive, and reproducible [19,21], the cellular assays are more relevant considering such parameters as the bioavailability and bioaccessibility of the tested compounds [18]. The best correlations of antioxidant capacities with concentrations of silymarin flavonoid/flavonolignans was demonstrated for the sum of silymarin components, followed by silychristin as one of the strongest antioxidants of the silymarin complex [16]. The total concentrations of all flavonoid/flavonolignans present in the supplements plotted against the relative radical scavenging activity gave the significant correlation with ORAC (R^2^ = 0.65) and ABTS (R^2^ = 0.52) followed by non-significant correlation with CAA (R^2^ = 0.10) and DPPH (R^2^ = 0.13). The inappropriateness of DPPH for some antioxidants was previously published [30]. Many samples that have the ability to reduce radicals in chemical assays failed to do so in cellular assays [25], which corresponds to our results, where 38% of the tested supplements failed to scavenge 50% of cellular radicals up to a concentration of 25 mg/L (Table 4). The highest tested concentration in CAA (25 mg/L) corresponds to a molar concentration of 26 µM of silymarin. In fact, such a large concentration is not expected in plasma, where the usual concentration is mainly in the nanomolar range and only in rare cases reaches the micromolar level [16]. Taking into account the silymarin bioavailability of 1% (as reported for silybin A/B and rats [16]), and the recommended daily dose of capsules, only two of the tested supplements (17 and 20) can lead to such concentrations in cells that can scavenge 50% of oxygen radicals inside.

The antioxidant activity of the commercially available silymarin SA extract determined by all of the used assays was significantly higher than the activity determined in the flavonolignans mixture mimicking silymarin SA, pointing to the presence of other non-silymarin antioxidants. A significant number of compounds with described biological activities (not only antioxidative) was also determined in the investigated commercially based dietary supplements (Supplementary Tables S3 and S4). The best correlation, especially in the cellular assay, was achieved for the sum of total phenolics (simple phenolics and flavonoids) occurring in *Silybum marianum*. These flavonoids have been reported many times in the literature for their antioxidant properties, which have been summarized e.g., in comprehensive reviews [31,32,33] or in publications focused on individual compounds, e.g., quercetin [34], rutin [35], luteolin [36], apigenin [37], and genistein [38]. In contrast to silymarin, these flavonoids are more bioavailable, i.e., rapidly absorbed from the small intestine and found in plasma [39]. Although the silymarin complex is beneficial for its antioxidant capacity, the effect of other antioxidants originating from the *Silybum marianum* should not be omitted when assessing the results of in vivo tests and / or clinical studies. To verify the presented results, a detailed in vivo evaluation of the samples must be perforSmed.

## Figures and Tables

**Table 1:** Characterization of commercial milk thistle-based dietary supplements as specified by the producer.

No.	Milk Thistle Extract	Other Components of Preparation
1	Milk thistle extract (*Silybum marianum*—seed) 250 mg in 1 capsule—standardized to contain 70% silymarin	*Schizandra chinensis* extract—100 mg in 1 capsule
2	Milk thistle extract (*Silybum marianum*—seed) standardized to contain 80% of silymarin complex, 250 mg of silymarin in 1 capsule	
3	Milk thistle standardized extract (*Silybum marianum*—seed), 100 mg of silymarin in 1 capsule (note: % of silymarin not possible to calculate)	
4	Milk thistle powder (*Silybum marianum*—freeze-grinded seeds)—390 mg in 1 capsule standardized to contain a minimum of 1.5% silymarin	
5	Milk thistle extract (*Silybum marianum*—seed), 150 mg in 1 capsule (note: % of silymarin not possible to calculate)	*Cordyceps sinensis* extract—50 mg in 1 capsule, *Scutellaria baicalensis* extract—50mg in one capsule
6	Silymarin from milk thistle standardized extract (*Silybum marianum*—seed), 100 mg of silymarin in 2 capsules ^1^	
7	Milk thistle extract (*Silybum marianum*—seed), 175 mg in 1 capsule—standardized to contain a minimum of 80% Silymarin	
8	Milk thistle seed extract (*Silybum marianum*) 140 mg in 1 capsule—silymarin (by UV) 98 mg (i.e., 70% of silymarin) ^2^	
9	Milk thistle seed extract (*Silybum marianum*) 140 mg in 1 capsule—silymarin (by UV) 98 mg (i.e., 70% of silymarin) ^2^	
10	Milk thistle extract (*Silybum marianum*—seed) 250 mg in 1 capsule—a 4:1 extract, equivalent to 1000 mg of milk thistle seed (note: % of silymarin not possible to calculate)	
11	Milk thistle extract (*Silybum marianum*-seed) 525 mg in 3 capsules—standardized to contain 80% silymarin, 420 mg	
12	Milk thistle extract (*Silybum marianum*—seed) 525 mg in 3 capsules—standardized to contain 80% silymarin, 420 mg	
13	Milk thistle extract (*Silybum marianum*—seed) 175 mg in 1 capsule—standardized to contain 80% silymarin, 140 mg	
14	Milk thistle extract (*Silybum marianum*—seed) 175 mg in 1 capsule—standardized to contain 80% silymarin, 140 mg	
15	Milk thistle extract (*Silybum marianum*—seed) 175 mg in 1 capsule—standardized to contain 80% silymarin, 140 mg	
16	Milk thistle extract (*Silybum marianum*—seed) 250 mg in 1 capsule—a 4:1 extract, equivalent to 1000 mg whole herb (note: % of silymarin not possible to calculate)	
17	Milk thistle seed extract 175 mg in 1 capsule—standardized to 80% silymarin (140 mg)	*Cnicus benedictus* (stem, leaf, flower) 120 mg in 1 capsule
18	Milk thistle seed extract 175 mg in 1 capsule—standardized to 80% silymarin (140 mg)	*Cnicus benedictus* (stem, leaf, flower) 120 mg in 1 capsule
19	Milk thistle seed extract 175 mg in 1 capsule—standardized to 80% silymarin (140 mg)	*Cnicus benedictus* (stem, leaf, flower) 120 mg in 1 capsule
20	Milk thistle extract (*Silybum marianum*—seed) 240 mg in 2 capsules—standardized to contain 80% silymarin, 192 mg	^3^
21	Milk thistle extract (*Silybum marianum*—seed) 240 mg in 2 capsules—standardized to contain 80% silymarin, 192 mg	^3^
22	Milk thistle extract (*Silybum marianum*—seed) 240 mg in 2 capsules—standardized to contain 80% silymarin, 192 mg	^3^
23	Milk thistle extract (*Silybum marianum*—seed) 240 mg in 2 capsules—standardized to contain 80% silymarin, 192 mg	^3^
24	Milk thistle extract (*Silybum marianum*—seed) 175 mg in 1 capsule—standardized to contain 80% silymarin, 140 mg	
25	Milk thistle extract (*Silybum marianum*—seed) 175 mg in 1 capsule—standardized to contain 80% silymarin, 140 mg	
26	Milk thistle extract (*Silybum marianum*—seed) 250 mg in 1 capsule—standardized to contain a minimum of 80% silymarin	

^1^ The amount of milk thistle extract is unknown. ^2^ Percentage of silymarin calculated from the declared amounts of *Silybum marianum* extract and declared amount of silymarin. ^3^ XTRA Premium Blend^®^ 240 mg in 2 capsules, Fennel (*Foeniculum vulgare*—seed), Dandelion (*Taraxacum officinale*—root), Licorice (*Glycyrrhiza glabra*—root).

**Table 2:** Composition of commercially available silymarin SA [mg/g] and its constituents analysed by U-HPLC-HRMS/MS. Data are presented with SDs.

Compound	Summary Formula	MW [g/mol]	Concentration [mg/g]
Taxifolin	C_15_H_12_O_7_	304.25	25.3 ± 0.8
Silychristin	C_25_H_22_O_10_	482.44	110 ± 3.4
Silydianin	C_25_H_22_O_10_		32.0 ± 0.9
Silybin A	C_25_H_22_O_10_		113 ± 3.1
Silybin B	C_25_H_22_O_10_		133 ± 3.9
Isosilybin A	C_25_H_22_O_10_		67.4 ± 2.4
Isosilybin B	C_25_H_22_O_10_		26.1 ± 1.1
2,3-Dehydrosilybin	C_25_H_20_O_10_	480.43	5.0 ± 0.3
SILYMARIN (sum of constituents)			51,182 ± 2764
Silymarin content in commercial preparation (%)			51.2 ± 2.8

**Table 3:** Content of silymarin (mg/g) and its constituents in tested milk thistle-based dietary supplements analysed by ultra-high performance liquid chromatography high-resolution tandem mass spectrometry (U-HPLC-HRMS/MS). Data are presented with standard deviations (SDs). The statistical significance was assessed by one-way analysis of variance (ANOVA) followed by Duncan’s post-hoc test (*p* > 0.05), and is indicated by superscripts. Statistically significant levels were denoted with different letters.

Formula No.	Taxifolin C_15_H_12_O_7_	Silychristin C_25_H_22_O_10_	Silydianin C_25_H_22_O_10_	Silybin A C_25_H_22_O_10_	Silybin B C_25_H_22_O_10_	Isosilybin A C_25_H_22_O_10_	Isosilybin B C_25_H_22_O_10_	2,3-dehydrosilybin C_25_H_20_O_10_	SILYMARIN—Sum of Constituents
1	6.7 ± 0.2 ^g,h^	49.7 ± 1.5 ^m,n^	13.4 ± 0.4 ^g,h^	89.4 ± 2.4 ^o^	110.7 ± 3.2 ^n^	24.0 ± 0.9 ^k^	8.0 ± 0.3 ^l^	0.9 ± 0.0 ^c,d^	302.8 ± 16.4 ^q^
2	4.3 ± 0.1 ^e^	39.8 ± 1.2 ^i,j^	13.3 ± 0.4 ^g,h^	17.3 ± 0.5 ^c,d^	29.0 ± 0.8 ^d^	18.0 ± 0.6 ^h,i^	6.6 ± 0.3 ^h,i,j,k^	1.3 ± 0.1 ^h,i^	129.5 ± 7.0 ^e,f,g^
3	2.0 ± 0.0 ^c^	12.2 ± 0.1 ^c^	4.7 ± 0.1 ^c^	21.6 ± 0.1 ^d,e^	5.2 ± 0.2 ^c^	5.5 ± 0.0 ^b^	0.5 ± 0.0 ^b^	0.5 ± 0.0b	69.8 ± 3.8 ^c,d^
4	0.5 ± 0.1 ^a^	3.7 ± 0.1 ^c^	2.0 ± 0.1 ^b^	4.4 ± 0.6 ^a,b^	21.5 ± 0.6 ^a^	1.4 ± 0.2 ^a^	1.9 ± 0.1 ^a^	0.1 ± 0.0 ^a^	17.8 ± 1.0 ^a,b^
5	0.1 ± 0.0 ^a^	1.0 ± 0.0 ^a^	0.6 ± 0.0 ^a^	1.1 ± 0.0 ^a^	1.9 ± 0.1 ^a^	0.5 ± 0.0 ^a^	0.2 ± 0.0 ^a^	0.1 ± 0.0a	5.4 ± 0.3 ^a^
6	1.2 ± 0.0 ^b^	9.2 ± 0.3 ^c^	4.3 ± 0.1 ^c^	13.4 ± 0.4 ^c^	13.8 ± 0.4 ^b^	4.3 ± 0.2 ^b^	1.7 ± 0.1 ^b^	0.4 ± 0.0 ^b^	48.5 ± 2.6 ^b,c^
7	3.0 ± 0.1 ^d^	17.5 ± 0.5 ^d^	8.2 ± 0.2 ^e^	28.1 ± 0.8 ^f,g^	32.0 ± 0.9 ^d,e^	8.2 ± 0.3 ^c^	3.0 ± 0.1 ^c^	0.9 ± 0.0 ^c,d^	100.9 ± 5.4 ^d,e^
8	5.3 ± 0.2 ^f^	35.9 ± 1.1 ^g,h^	14.0 ± 0.4 ^h^	59.1 ± 1.6 ^k^	73.2 ± 2.1 ^j^	17.7 ± 0.6 ^h,i^	8.0 ± 0.3 ^l^	2.4 ± 0.1 ^l^	215.5 ± 11.6 ^m,n,o^
9	7.0 ± 0.2 ^h^	40.6 ± 1.3 ^i,j,k^	12.6 ± 0.4 ^g^	70.7 ± 1.9 ^m,n^	84.6 ± 2.5 ^l,m^	19.1 ± 0.7 ^i,j^	6.9 ± 0.3 ^i,j,k^	1.2 ± 0.1 ^h,i^	242.7 ± 13.1 ^o,p^
10	0.4 ± 0.0 ^a^	4.1 ± 0.1 ^a,b^	0.6 ± 0.0 ^a^	6.6 ± 0.2 ^b^	6.0 ± 0.2 ^a^	1.8 ± 0.1 ^a^	0.8 ± 0.0 ^a^	0.5 ± 0.0 ^b^	20.8 ± 1.1 ^a,b^
11	5.1 ± 0.0 ^f^	31.8 ± 1.0 ^e,f^	9.7 ± 0.3 ^f^	46.6 ± 1.3 ^i^	58.4 ± 1.7 ^h,i^	13.9 ± 0.5 ^d,e,f^	4.9 ± 0.2 ^d,e^	1.0 ± 0.1 ^d,e,f^	171.4 ± 9.4 ^h,i,j,k^
12	7.9 ± 0.3 ^i,j^	38.2 ± 1.2 ^g,h,i^	6.2 ± 0.2 ^d^	27.8 ± 0.8 ^f,g^	38.6 ± 1.1 ^f^	15.5 ± 0.6 ^e,g^	4.8 ± 0.2 ^d^	1.9 ± 0.1 ^k^	140.8 ± 7.6 ^f,g,h^
13	4.8 ± 0.2 ^e,f^	31.5 ± 1.0 ^e,f^	8.8 ± 0.2 ^e,f^	18.8 ± 0.5 ^d^	27.2 ± 0.8 ^d^	13.4 ± 0.5 ^d^	5.3 ± 0.2 ^d,e,f^	1.2 ± 0.1 ^g,h,i^	111.1 ± 6.0 ^e,f^
14	9.0 ± 0.3 ^k^	38.1 ± 1.2 ^g,h,i^	13.3 ± 0.4 ^g,h^	25.1 ± 0.7 ^e,f^	37.2 ± 1.1 ^e,f^	16.2 ± 0.6 ^g,h^	6.0 ± 0.3 ^f,g,h^	1.3 ± 0.1 ^h,i^	146.3 ± 7.9 ^g,h,i^
15	8.6 ± 0.3 ^j,k^	38.0 ± 1.2 ^g,h,i^	12.3 ± 0.3 ^g^	42.2 ± 1.1 ^h^	54.0 ± 1.6 ^g,h^	15.5 ± 0.6 ^e,f,g^	5.7 ± 0.2 ^e,f,g^	1.2 ± 0.1 ^g,h,i^	177.5 ± 9.6 ^i,j,k^
16	0.6 ± 0.0 ^a,b^	4.9 ± 0.2 ^b^	1.7 ± 0.0 ^a,b^	6.6 ± 0.2 ^b^	6.8 ± 0.2 ^a^	2.3 ± 0.1 ^a^	0.9 ± 0.0 ^a^	0.4 ± 0.0 ^b^	24.2 ± 1.3 ^a,b^
17	11.0 ± 0.4 ^m,n^	66.8 ± 2.1 ^p^	34.8 ± 1.0 ^l^	105.5 ± 2.8 ^p^	130.2 ± 3.8 ^o^	30.9 ± 1.1 ^m^	11.7 ± 0.5 ^o^	1.6 ± 0.1 ^j^	392.5 ± 21.2 ^s^
18	12.9 ± 0.4 ^p^	58.8 ± 1.8 ^o^	27.4 ± 0.8 ^k^	92.4 ± 2.5 ^o^	111.3 ± 3.2 ^n^	27.7 ± 1.0 ^l^	10.3 ± 0.4 ^n^	1.5 ± 0.1 ^j^	342.4 ± 18.5 ^r^
19	9.9 ± 0.3 ^l^	60.3 ± 1.9 ^o^	36.2 ± 1.0 ^m^	93.2 ± 2.5 ^o^	114.1 ± 3.3 ^n^	30.1 ± 1.1 ^m^	7.1 ± 0.3 ^i,j,k^	1.9 ± 0.1 ^k^	352.7 ± 19.0 ^r^
20	6.1 ± 0.2 ^g^	44.2 ± 1.4 ^k,l^	21.6 ± 0.6 ^j^	41.3 ± 1.1 ^h^	60.0 ± 1.7 ^i^	20.1 ± 0.7 ^j^	8.9 ± 0.4 ^m^	1.4 ± 0.1 ^i^	203.6 ± 11.0 ^l,m,n^
21	10.7 ± 0.3 ^m^	42.8 ± 1.3 ^j,k^	12.1 ± 0.3 ^g^	69.0 ± 1.9 ^m^	81.8 ± 2.4 ^k,l^	18.7 ± 0.7 ^ij^	6.3 ± 0.3 ^g,h,i^	0.8 ± 0.0 ^c,d^	242.3 ± 13.1 ^o,p^
22	11.6 ± 0.4 ^n,o^	46.6 ± 1.4 ^l,m^	13.1 ± 0.4 ^g,h^	74.0 ± 2.0 ^n^	87.8 ± 2.5 ^m^	20.0 ± 0.7 ^j^	7.3 ± 0.3 ^k,l^	0.9 ± 0.0 ^c,d,e^	261.3 ± 14.1 ^p^
23	12.2 ± 0.4 ^o,p^	51.6 ± 1.6 ^n^	15.9 ± 0.4 ^i^	31.4 ± 0.8 ^g^	52.9 ± 1.5 ^g,h^	20.4 ± 0.7 ^j^	7.2 ± 0.3 ^j,k^	1.6 ± 0.1 ^j^	193.2 ± 10.4 ^k,l,m^
24	8.8 ± 0.3 ^k^	34.5 ± 1.1 ^f,g^	9.9 ± 0.3 ^f^	52.0 ± 1.4 ^j^	62.9 ± 1.8 ^i^	13.6 ± 0.5 ^d,f^	5.1 ± 0.2 ^d,e^	0.8 ± 0.0 ^c^	187.5 ± 10.1 ^k,l,m^
25	7.7 ± 0.2 ^i^	30.5 ± 0.9 ^e^	12.1 ± 0.3 ^g^	41.5 ± 1.1 ^h^	50.9 ± 1.5 ^g^	12.8 ± 0.5 ^d^	5.0 ± 0.2 ^d,e^	0.9 ± 0.0 ^c,d^	161.4 ± 8.7 ^g,h,i,j^
26	9.3 ± 0.3 ^k,l^	39.6 ± 1.2 ^h,i,j^	15.3 ± 0.4 ^i^	64.2 ± 1.7 ^l^	76.4 ± 2.2 ^j,k^	17.5 ± 0.6 ^h,i^	6.4 ± 0.3 ^g,h,i,j^	1.1 ± 0.1 ^e,f,g^	230.0 ± 12.4 ^n,o,p^

**Table 4:** Concentration of milk thistle-based dietary supplement that gives half-maximal response (EC_50_, mg/L) for different antioxidant activity assays. The lower is the EC_50_ value, the higher is the anti-radical activity. The EC_50_ values were obtained using the GraphPad Prism 7 software, and are presented as an average of three replicates with standard errors of the mean (SEM). The statistical significance was assessed by one-way analysis of variance (ANOVA) followed by Duncan’s post-hoc test (*p* > 0.05), and is indicated by superscripts. Statistically significant levels were denoted with different letters.

Sample No.	EC_50_ [mg/L]			
	ABTS	ORAC	DPPH	CAA
1	8.5 ± 0.5 ^a^	43.3 ± 3.2 ^f,g^	120.8 ± 6.9 ^c,d^	>25.0
2	17.4 ± 2.2 ^b^	30.7 ± 3.0 ^e^	73.0 ± 2.8 ^b^	>25.0
3	8.7 ± 0.9 ^a^	90.8 ± 6.4 ^i^	9.7 ± 2.3 ^a^	11.3 ± 0.2 ^a^
4	>66.7	>200.0	>166.7	>25.0
5	>66.7	>200.0	>166.7	>25.0
6	>66.7	122.8 ± 14.7 ^j^	>166.7	>25.0
7	38.7 ± 2.3 ^h^	56.9 ± 1.3 ^h^	>166.7	>25.0
8	18.7 ± 2.0 ^b^	53.8 ± 0.7 ^g,h^	108.3 ± 7.0 ^c^	21.2 ± 3.6 ^b,c,d,e,f^
9	20.4 ± 1.2 ^b,d^	30.5 ± 3.3 ^e^	58.7 ± 15.2 ^b^	24.3 ± 4.0 ^d,e,f^
10	>66.7	>200.0	>166.7	>25.0
11	25.7 ± 1.8 ^d,e,f^	31.0 ± 3.3 ^e^	>166.7	21.8 ± 2.9 ^c,d,e,f^
12	27.6 ± 3.0 ^e,f,g^	26.9 ± 0.7 ^c,d,e^	130.4 ± 9.2 ^c,d^	27.3 ± 2.6 ^f^
13	32.3 ± 2.8 ^g^	33.8 ± 5.1 ^e,f^	> 166.7	>25.0
14	27.9 ± 3.1 ^e,f,g^	28.8 ± 0.1 ^d,e^	128.3 ± 15.0 ^c,d^	13.5 ± 1.7 ^a,b,c^
15	48.4 ± 0.7 ^i^	53.5 ± 0.7 ^g,h^	> 166.7	21.7 ± 3.5 ^c,d,e,f^
16	>66.7	>200.0	>166.7	>25.0
17	12.0 ± 0.3 ^a,c^	13.9 ± 0.5 ^a,b^	51.0 ± 1.4 ^b^	12.5 ± 0.7 ^a^
18	9.9 ± 0.3 ^a^	16.7 ± 2.4 ^a,b,c,d^	106.9 ± 3.0 ^c^	16.9 ± 2.3 ^a,b,c,d^
19	10.3 ± 0.7 ^a^	16.2 ± 2.0 ^a,b,c^	131.4 ± 10.5 ^c,d^	17.9 ± 0.7 ^a,b,c,d,e^
20	20.9 ± 2.8 ^b,d^	12.6 ± 1.2 ^a^	143.4 ± 3.0 ^d^	16.7 ± 0.8 ^a,b,c,d^
21	17.5 ± 1.9 ^b,c^	24.8 ± 0.6 ^b,c,d,e^	128.4 ± 2.9 ^c,d^	18.5 ± 3.0 ^a,b,c,d,e^
22	18.0 ± 1.8 ^b^	27.4 ± 1.0 ^c,d,e^	125.4 ± 9.8 ^c,d^	26.4 ± 2.1 ^e,f^
23	20.7 ± 1.1 ^b,d^	28.7 ± 0.1 ^d,e^	145.9 ± 9.0 ^d^	18.4 ± 0.8 ^a,b,c,d,e^
24	23.5 ± 1.8 ^b,d,e^	59.6 ± 4.5 ^h^	206.5 ± 1.1 ^f^	22.8 ± 3.6 ^d,e,f^
25	31.2 ± 2.4 ^f,g^	44.2 ± 3.6 ^f,g^	116.0 ± 7.8 ^c,d^	>25.0
26	17.7 ± 1.5 ^b,c^	25.8 ± 0.6 ^c,d,e^	181.5 ± 2.7 ^e^	12.9 ± 1.4 ^a,b^
Silymarin SA	12.8 ± 2.6	11.0 ± 0.1	>166.7	18.5 ± 3.7
Silymarin mimicking mixture of flavonoid/flavonolignans	1.7 ± 0.3	23.4 ± 2.7	>166.7	>25.0
Quercetin	>1.7	5.6 ± 0.1	4.5 ± 0.1	1.0 ± 0.0
Trolox	>6.7	>200.0	5.1 ± 0.1	0.7 ± 0.1
Silibinin SA	9.1 ± 0.9	19.2 ± 2.2	>166.7	>25.0

**Table 5:** Correlation coefficients (R^2^) of the dependence of the antioxidant activity of 26 dietary supplements on the concentration of silymarin constituents and overall silymarin.

Assay	Silymarin Constituents								
	taxifolin	silychristin	silydianin	silybin A	silybin B	isosilybin A	isosilybin B	2,3-dehydrosilybin	Sum of Silymarin Constituents
ABTS	0.159	0.402	0.453 ^a^	0.530 ^a^	0.527 ^a^	0.493 ^a^	0.417	0.087	0.520 ^a^
ORAC	0.685 ^a^	0.813 ^a^	0.617 ^a^	0.497 ^a^	0.571 ^a^	0.774 ^a^	0.745 ^a^	0.553^a^	0.652 ^a^
DPPH	0.472 ^a^	0.219	0.014	0.092	0.110	0.127	0.093	0.160	0.133
CAA	0.055	0.054	0.366	0.058	0.062	0.111	0.144	0.139	0.098

^a^ Correlation coefficient confirms (α = 0.05) that the results of antioxidant assay linearly depend on the concentration of silymarin constituents or overall silymarin (ABTS df = 19, critical value = 0.433; ORAC df = 20, critical value = 0.423; DPPH df = 15, critical value = 0.448; CAA df = 14, critical value = 0.497).

## Supplementary Materials

The following are available online at https://www.mdpi.com/2076-3921/8/8/317/s1. Table S1: List of non-silymarin bioactive compounds reported in literature for *Silybum marianum* (SM), *Schisandra chinensis* (SCH), *Cordyceps sinensis* (CS), *Scutellaria baicalensis* (SB), *Cnicus benedictus* (CB*), Foeniculum vulgare* (FV), *Taraxacum officinale* (TO), and *Glycyrrhiza glabra* (GG). Table S2: Characteristics of the antioxidant compounds identified by the U-HPLC-HRMS/MS targeted screening. Table S3: Correlation coefficients (R^2^) of dependence of antioxidant activity of 26 dietary supplements on U-HPLC-HRMS/MS responses of non-silymarin antioxidants present in *Silybum marianum*. Table S4: Correlation coefficients (R^2^) of dependence of antioxidant activity of 26 dietary supplements on U-HPLC-HRMS/MS responses of non-silymarin antioxidants present in other plants—*Schisandra chinensis*, *Cordyceps sinensis, Scutellaria baicalensis*, *Cnicus benedictus*, *Foeniculum vulgare*, *Taraxacum officinale,* and *Glycyrrhiza glabra*.

## Abbreviations

AAPH	2,2′-azo-*bis*-(2-methylpropionamidine) dihydrochloride
ABTS	2,2’-azino-*bis*-(3-ethylbenzothiazoline-6-sulphonic acid)
DPPH	2,2-diphenyl-1-picrylhydrazyl
DCFH-DA	2′,7′-dichlorofluorescin diacetate
CAA	cellular antioxidant activity
EMEM	Eagle’s Minimum Essential Medium
FBS	fetal bovine serum
HepG2	human hepatocellular adenocarcinoma
HRMS/MS	high resolution tandem mass spectrometry
ORAC	oxygen radical absorption capacity
PBS	phosphate buffered saline
PTFE	polytetrafluoroethylene
SA	Sigma-Aldrich
U-HPLC	ultra-high performance liquid chromatography
U-HPLC-HRMS/MS	ultra-high performance liquid chromatography high-resolution tandem mass spectrometry
