# Supplementary material for: Complex Evaluation of Antioxidant Capacity of Milk Thistle Dietary Supplements
Source: Antioxidants (Basel). 2019 Aug 18;8(8):317. doi: 10.3390/antiox8080317 (PMC6720444; doi:10.3390/antiox8080317)
Supplement: Supplementary file 1 [file antioxidants-08-00317-s001.zip › antioxidants-550286-Supplementary-proofback_04-send XML-01.docx]

Article

Complex Evaluation of Antioxidant Capacity of Milk Thistle Dietary Supplements

Jitka Viktorova ^1^, Milena Stranska-Zachariasova ^2,^*, Marie Fenclova ^2^, Libor Vitek ^3^,
Jana Hajslova ^2^, Vladimir Kren ^4^ and Tomas Ruml ^1^

^1^ Department of Biochemistry and Microbiology, University of Chemistry and Technology Prague, Technická 5, 166 28 Prague, Czech Republic

^2^ Department of Food Analysis and Nutrition, University of Chemistry and Technology Prague,
Technická 5, 166 28 Prague, Czech Republice

^3^ 4^th^ Department of Internal Medicine and Institute of Medical Biochemistry and Laboratory Diagnostics, 1^st^ Faculty of Medicine, Charles University, Katerinska 32, 12000 Prague, Czech Republic

^4^ Laboratory of Biotransformation, Institute of Microbiology, Czech Academy of Sciences, Vídeňská 1083, 142 20 Prague, Czech Republice

***** Correspondence: milena.stranska@vscht.cz

Supplementary Table 1 List of non-silymarin bioactive compounds reported in literature for *Silybum marianum* (SM)^1, 2, 3-10^, *Schisandra chinensis* (SCH)^11^, *Cordyceps sinensis* (CS)^12, 13^, *Scutellaria baicalensis* (SB)^14, 15^, *Cnicus benedictus* (CB)^16^, *Foeniculum vulgare* (FV)^17, 18^, *Taraxacum officinale* (TO)^19^ and *Glycyrrhiza glabra* (GG)^20^.

| **Compound name** | **Summary formula** | **Plant of origin** |
| --- | --- | --- |
| neusilychristin | C_25_H_22_O_10_ | *SM* |
| silyamandin | C_25_H_22_O_11_ | *SM* |
| isosilandrin A | C_25_H_22_O_9_ | *SM* |
| isosilandrin B | C_25_H_22_O_9_ | *SM* |
| neosilyhermin A | C_25_H_22_O_9_ | *SM* |
| neosilyhermin B | C_25_H_22_O_9_ | *SM* |
| silandrin A | C_25_H_22_O_9_ | *SM* |
| silandrin B | C_25_H_22_O_9_ | *SM* |
| silyhermin | C_25_H_22_O_9_ | *SM* |
| silymonin | C_25_H_22_O_9_ | *SM* |
| apigenin | C_15_H_10_O_5_ | *SM* |
| genistein | C_15_H_10_O_5_ | *SM* |
| luteolin | C_15_H_10_O_6_ | *SM* |
| kaempferol | C_15_H_10_O_6_ | *SM* |
| quercetin | C_15_H_10_O_7_ | *SM* |
| myricetin | C_15_H_10_O_8_ | *SM* |
| naringenin | C_15_H_12_O_5_ | *SM* |
| dihydrokaempferol (aromadendrin) | C_15_H_12_O_6_ | *SM* |
| eriodictyol | C_15_H_12_O_6_ | *SM* |
| catechin | C_15_H_14_O_6_ | *SM* |
| acacetin | C_16_H_12_O_5_ | *SM* |
| genkwanin | C_16_H_12_O_5_ | *SM* |
| hispidulin | C_16_H_12_O_6_ | *SM* |
| chrysoeriol | C_16_H_12_O_6_ | *SM* |
| kaempferol-3-methyl ether (isokaempferide) | C_16_H_12_O_6_ | *SM* |
| nepetin | C_16_H_12_O_7_ | *SM* |
| rhamentin | C_16_H_12_O_7_ | *SM* |
| patuletin | C_16_H_12_O_8_ | *SM* |
| apigenin-5,7-dimethyl ether | C_17_H_14_O_5_ | *SM* |
| pectolinarigenin | C_17_H_14_O_6_ | *SM* |
| kumatakenin | C_17_H_14_O_6_ | *SM* |
| jaceosidin | C_17_H_14_O_7_ | *SM* |
| eupatilin | C_18_H_16_O_7_ | *SM* |
| sudachitin | C_18_H_16_O_8_ | *SM* |
| hymenoxin | C_19_H_18_O_8_ | *SM* |
| quercetin-3-O-arabinoside | C_20_H_18_O_11_ | *SM* |
| luteolin-7-O-glucuronide | C_21_H_17_O_12_ | *SM* |
| apigenin-7-O-glucuronide | C_21_H_18_O_11_ | *SM* |
| miquelianin (quercetin 3-glucoronide) | C_21_H_18_O_13_ | *SM* |
| isovitexin | C_21_H_20_O_10_ | *SM* |
| apigenin-7-O-glucoside | C_21_H_20_O_10_ | *SM* |
| vitexin | C_21_H_20_O_10_ | *SM* |
| isoorientin | C_21_H_20_O_11_ | *SM* |
| orientin | C_21_H_20_O_11_ | *SM* |
| kaempferol-3-O-beta-D-glucoside (astragalin) | C_21_H_20_O_11_ | *SM* |
| luteolin-7-O-glucoside (cynaroside) | C_21_H_20_O_11_ | *SM* |
| quercetin 3-O-galactoside (hyperoside) | C_21_H_20_O_12_ | *SM* |
| quercetin 3-O-glucoside (isoquercetin) | C_21_H_20_O_12_ | *SM* |
| spiraeoside (quercetin 4-O-glucoside) | C_21_H_20_O_12_ | *SM* |
| naringenin 7-O-beta-D-glucopyranoside | C_21_H_22_O_10_ | *SM* |
| isokaempferide 7-rhamnoside | C_22_H_22_O_10_ | *SM* |
| isorhamnetin 3-O-glucoside | C_22_H_22_O_12_ | *SM* |
| apigenin-4,7-diglucoside | C_27_H_30_O_15_ | *SM* |
| kaempferol-3-rutinoside | C_27_H_30_O_15_ | *SM* |
| rutin | C_27_H_30_O_16_ | *SM* |
| naringin | C_27_H_32_O_14_ | *SM* |
| hydroxybenzoic acid (salicylic acid) | C_7_H_6_O_3_ | *SM* |
| beta-resorcylic acid (dihydroxybenzoic acid) | C_7_H_6_O_4_ | *SM* |
| gallic acid | C_7_H_6_O_5_ | *SM* |
| guaiacol | C_7_H_8_O_2_ | *SM* |
| vanillic acid | C_8_H_8_O_4_ | *SM* |
| syringaldehyde | C_9_H_10_O_4_ | *SM* |
| syringic acid | C_9_H_10_O_5_ | *SM* |
| coumaric acid | C_9_H_8_O_3_ | *SM* |
| caffeic acid | C_9_H_8_O_4_ | *SM* |
| coniferylaldehyd | C_10_H_10_O_3_ | *SM* |
| ferulic acid | C_10_H_10_O_4_ | *SM* |
| dihydroconiferyl alcohol | C_10_H_14_O_3_ | *SM* |
| ethyl caffeate | C_11_H_12_O_4_ | *SM* |
| methyl ferulate | C_11_H_12_O_4_ | *SM* |
| sinapinic acid | C_11_H_12_O_5_ | *SM* |
| ellagic acid | C_14_H_6_O_8_ | *SM* |
| 3-O-caffeoylquinic acid (PA1) (chlorogenic acid) | C_16_H_18_O_9_ | *SM* |
| 4-O-caffeoylquinic acid (PA3) | C_16_H_18_O_9_ | *SM* |
| 5-O-feruloylquinic acid (PA4) | C_17_H_20_O_9_ | *SM* |
| 1,5-O- dicaffeoylquinic acid (PA6) | C_25_H_24_O_12_ | *SM* |
| 3,5-O- dicaffeoylquinic acid (PA5) | C_25_H_24_O_12_ | *SM* |
| 4,5-O-dicaffeoylquinic acid (PA7) | C_25_H_24_O_12_ | *SM* |
| cynarin | C_25_H_24_O_12_ | *SM* |
| mariamide A | C_42_H_46_N_4_O_10_ | *SM* |
| mariamide B | C_21_H_24_N_2_O_5_ | *SM* |
| 3- methylcarboxymethyl-indole-1-N-beta-D-glucopyranoside | C_16_H_19_NO_7_ | *SM* |
| angeloylgomisin H | C_28_H_36_O_8_ | *SCH* |
| angeloylgomisin P | C_28_H_34_O_9_ | *SCH* |
| angeloylgomisin Q | C_29_H_38_O_9_ | *SCH* |
| benzoylgomisin H | C_30_H_34_O_8_ | *SCH* |
| benzoylgomisin O | C_30_H_32_O_8_ | *SCH* |
| gomisin A | C_23_H_28_O_7_ | *SCH* |
| gomisin B | C_28_H_34_O_9_ | *SCH* |
| gomisin D | C_28_H_34_O_10_ | *SCH* |
| gomisin F | C_28_H_34_O_9_ | *SCH* |
| gomisin G | C_30_H_32_O_9_ | *SCH* |
| gomisin J | C_22_H_28_O_6_ | *SCH* |
| gomisin K1, K2, K3 | C_23_H_30_O_6_ | *SCH* |
| gomisin M1, M2, L1, L2 | C_22_H_26_O_6_ | *SCH* |
| gomisin N | C_23_H_28_O_6_ | *SCH* |
| isoschisandrin | C_24_H_32_O_7_ | *SCH* |
| propinquanin F | C_28_H_36_O_8_ | *SCH* |
| schisandrin | C_24_H_32_O_7_ | *SCH* |
| schisandrin A | C_24_H_32_O_6_ | *SCH* |
| schisandrin B | C_23_H_28_O_6_ | *SCH* |
| schisandrin C | C_22_H_24_O_6_ | *SCH* |
| schisantherin A | C_30_H_32_O_9_ | *SCH* |
| schisantherin C | C_28_H_34_O_9_ | *SCH* |
| tigloylgomisin P | C_28_H_34_O_9_ | *SCH* |
| adenosine | C_10_H_13_N_5_O_4_ | *CS* |
| cordycedipeptide A | C_9_H_14_N_3_O_3_ | *CS* |
| cordycepic acid | C_6_H_14_O_6_ | *CS* |
| cordycepin | C_10_H_13_N_5_O_3_ | *CS* |
| cordypyridone A | C_16_H_23_NO_3_ | *CS* |
| cordypyridone B | C_16_H_23_NO_3_ | *CS* |
| dipicolinic acid | C_7_H_5_NO_4_ | *CS* |
| farinosone A | C_25_H_27_NO_4_ | *CS* |
| farinosone B | C_25_H_26_NO_5_ | *CS* |
| farinosone C | C_19_H_25_NO_5_ | *CS* |
| hypoxanthine | C_5_H_4_N_4_O | *CS* |
| macrolides | C_10_H_14_O_4_ | *CS* |
| militarinone A | C_26_H_37_NO_6_ | *CS* |
| militarinone B | C_26_H_33_NO_5_ | *CS* |
| militarinone C | C_26_H_33_NO_4_ | *CS* |
| militarinone D | C_26_H_31_NO_4_ | *CS* |
| myriocin | C_21_H_39_NO_6_ | *CS* |
| N-acetylgalactosamine | C_8_H_15_NO_6_ | *CS* |
| naphthaquinone | C_10_H_6_O_2_ | *CS* |
| paecilomycine A | C_15_H_22_O_4_ | *CS* |
| paecilomycine B | C_15_H_22_O_5_ | *CS* |
| paecilomycine C | C_15_H_20_O_4_ | *CS* |
| paecilosetin | C_22_H_31_NO_4_ | *CS* |
| spirotenuipesine A | C_15_H_22_O_4_ | *CS* |
| spirotenuipesine B | C_15_H_22_O_5_ | *CS* |
| 2-(4-hydroxy phenyl) ethyl-O-beta-D- glucopyranoside | C_14_H_20_O_7_ | *SB* |
| apigenin 7-O-glucoside | C_21_H_20_O_10_ | *SB* |
| baicalein | C_15_H_10_O_5_ | *SB* |
| baicalin | C_21_H_18_O_11_ | *SB* |
| caffeic acid | C_9_H_8_O_4_ | *SB* |
| isomartynoside | C_31_H_40_O_15_ | *SB* |
| martynoside | C_31_H_40_O_15_ | *SB* |
| neobaicalein (skullcapflavone II) | C_19_H_18_O_8_ | *SB* |
| oroxylin A | C_16_H_12_O_5_ | *SB* |
| oroxylin A glucoronide | C_22_H_20_O_11_ | *SB* |
| skullcapflavone I | C_17_H_14_O_6_ | *SB* |
| skullcapflavone I 2'-O-glucoside | C_23_H_24_O_11_ | *SB* |
| ursolic acid | C_30_H_48_O_3_ | *SB* |
| verbascoside | C_29_H_36_O_15_ | *SB* |
| wogonin | C_16_H_12_O_5_ | *SB* |
| wogonoside | C_22_H_20_O_11_ | *SB* |
| cinnamaldehyde | C_9_H_8_O | *CB* |
| fenchone | C_10_H_16_O | *CB* |
| citral | C_10_H_16_O | *CB* |
| salonitenolide | C_15_H_20_O_4_ | *CB* |
| cnicin | C_20_H_26_O_7_ | *CB* |
| arctigenin | C_21_H_24_O_6_ | *CB* |
| trachelogenin | C_21_H_24_O_7_ | *CB* |
| nortracheloside | C_26_H_32_O_12_ | *CB* |
| absinthin | C_30_H_40_O_6_ | *CB* |
| alpha-amyrenone | C_30_H_48_O | *CB* |
| alpha-amyrine | C_30_H_50_O | *CB* |
| alpha-amyrin acetate | C_32_H_52_O_2_ | *CB* |
| multiflorenol acetate | C_32_H_52_O_2_ | *CB* |
| p-anisaldehyde | C_8_H_8_O_2_ | *FV* |
| trans-anethole | C_10_H_12_O | *FV* |
| estragole | C_10_H_12_O | *FV* |
| fenchone | C_10_H_16_O | *FV* |
| kaempferol | C_15_H_10_O_6_ | *FV* |
| naringenin | C_15_H_12_O_5_ | *FV* |
| acacetin | C_16_H_12_O_5_ | *FV* |
| isorhamnetin | C_16_H_12_O_7_ | *FV* |
| photoanethole | C_16_H_16_O_2_ | *FV* |
| 3-O-caffeoylquinic acid | C_16_H_18_O_9_ | *FV* |
| 4-O-caffeoylquinic acid | C_16_H_18_O_9_ | *FV* |
| 5-O-caffeoylquinic acid | C_16_H_18_O_9_ | *FV* |
| sinapyl glucoside | C_17_H_22_O_9_ | *FV* |
| rosmarinic acid | C_18_H_16_O_8_ | *FV* |
| dianethole | C_19_H_22_O_2_ | *FV* |
| trans-resveratrol-3-O-beta-d-glucopyranoside | C_20_H_22_O_8_ | *FV* |
| kampferol-3-O-glucuronide | C_21_H_18_O_12_ | *FV* |
| quercetin-3-O-glucuronide | C_21_H_18_O_13_ | *FV* |
| kaempferol-3-O-glucoside | C_21_H_20_O_11_ | *FV* |
| quercetin-3-O-galactoside (hyperoside) | C_21_H_20_O_12_ | *FV* |
| isoquercetin | C_21_H_20_O_12_ | *FV* |
| isorhamnetin-3-O-glucoside | C_22_H_22_O_12_ | *FV* |
| syringin-4-O-beta-glucoside | C_23_H_34_O_14_ | *FV* |
| 1,3-O-di-caffeoylquinic acid | C_25_H_24_O_12_ | *FV* |
| 1,4-O-di-caffeoylquinic acid | C_25_H_24_O_12_ | *FV* |
| 1,5-O-di-caffeoylquinic acid | C_25_H_24_O_12_ | *FV* |
| kaempferol-3-O-rutinoside | C_27_H_30_O_15_ | *FV* |
| eriodictyol-7-rutinoside | C_27_H_32_O_15_ | *FV* |
| cis a trans-miyabenol C | C_42_H_32_O_9_ | *FV* |
| gallic acid | C_7_H_6_O_5_ | *TO* |
| esculetin | C_9_H_6_O_4_ | *TO* |
| caffeic acid | C_9_H_8_O_4_ | *TO* |
| apigenin | C_15_H_10_O_5_ | *TO* |
| luteolin | C_15_H_10_O_6_ | *TO* |
| quercetin | C_15_H_10_O_7_ | *TO* |
| taraxinic acid | C_15_H_18_O_4_ | *TO* |
| coumestrol | C_15_H_8_O_5_ | *TO* |
| chlorogenic acid | C_16_H_18_O_9_ | *TO* |
| artemetin | C_20_H_20_O_8_ | *TO* |
| luteolin-7-O-beta-D-glucopyranoside (cyranoside) | C_21_H_20_O_11_ | *TO* |
| isoquercetin | C_21_H_20_O_12_ | *TO* |
| beta-amyrin | C_30_H_50_O | *TO* |
| taraxerol | C_30_H_50_O | *TO* |
| taraxasterol | C_30_H_50_O | *TO* |
| taraxasteryl acetate | C_32_H_52_O_2_ | *TO* |
| lutein epoxide | C_40_H_56_O_3_ | *TO* |
| lutein | C_40_H_56_O_2_ | *TO* |
| 4-methyl coumarin | C_10_H_8_O_2_ | *GG* |
| liqcoumarin | C_12_H_10_O_4_ | *GG* |
| quercetin | C_15_H_10_O_7_ | *GG* |
| liquiritigenin | C_15_H_12_O_4_ | *GG* |
| isoliquiritigenin | C_15_H_12_O_4_ | *GG* |
| glyzaglabrin | C_16_H_10_O_6_ | *GG* |
| formononetin | C_16_H_12_O_4_ | *GG* |
| 7-methoxy-2-methylisoflavone | C_17_H_14_O_3_ | *GG* |
| 7-acetoxy-2-methyl-isoflavone | C_18_H_14_O_4_ | *GG* |
| glyzarin | C_18_H_14_O_4_ | *GG* |
| licoisoflavone B | C_20_H_16_O_6_ | *GG* |
| glabrene | C_20_H_18_O_4_ | *GG* |
| licoflavonol | C_20_H_18_O_6_ | *GG* |
| licoisoflavone A | C_20_H_18_O_6_ | *GG* |
| glabridin | C_20_H_20_O_4_ | *GG* |
| quercetin-3-glucoside | C_21_H_20_O_12_ | *GG* |
| isoliquiritin | C_21_H_22_O_9_ | *GG* |
| liquiritoside (liquiritin) | C_21_H_22_O_9_ | *GG* |
| glabrol | C_25_H_28_O_4_ | *GG* |
| licuraside | C_26_H_30_O_13_ | *GG* |
| isoglabrolide | C_30_H_44_O_4_ | *GG* |
| glabrolide | C_30_H_44_O_4_ | *GG* |
| liquoric acid | C_30_H_44_O_5_ | *GG* |
| liquiritic acid | C_30_H_46_O_4_ | *GG* |
| glycyrrhetinic acid (enoxolone) | C_30_H_46_O_4_ | *GG* |
| glycyrrhizin | C_42_H_62_O_16_ | *GG* |
| licoagrone | C_45_H_42_O_10_ | *GG* |

^1^ Abenavoli, L.M.; Capasso, R.; Milic, N.; Capasso, F. Milk thistle in liver diseases: Past, present, future. Phytother. Res. 2010, 24, 1423–1432.

^2^ Chambers, C.S.; Holečková, V.; Petrásková, L.; Biedermann, D.; Valentová, K.; Buchta, M.; Křen, V. The silymarin composition… and why does it matter??? Food Res. Int. 2017, 100, 339–353, doi:10.1016/j.foodres.2017.07.017.

^3^ Andrzejewska, J.; Martinelli, T.; Sadowska, K. Silybum marianum: Non-medical exploitation of the species. Ann. Appl. Boil. 2015, 167, 285–297.

^4^ Milić, N.; Milosević, N.; Suvajdzić, L.; Zarkov, M.; Abenavoli, L. New therapeutic potentials of milk thistle (Silybum marianum). Nat. Prod. Commun. 2013, 8, 1801–1810.

^5^ Csupor, D.; Csorba, A.; Hohmann, J. Recent advances in the analysis of flavonolignans of Silybum marianum. J. Pharm. Biomed. Anal. 2016, 130, 301–317.

^6^ de Oliveira, D.R.; Schaffer, L.F.; Busanello, A.; Barbosa, C.P.; Peroza, L.R.; de Freitas, C.M.; Krum, B.N.; Bressan, G.N.; Boligon, A.A.; Athayde, M.L.; et al. Silymarin has antioxidant potential and changes the activity of na+/k+-atpase and monoamine oxidase in vitro. Ind. Crop. Prod. 2015, 70, 347–355, doi:10.1016/j.indcrop.2015.03.060.

^7^ Lucini, L.; Kane, D.; Pellizzoni, M.; Ferrari, A.; Trevisi, E.; Ruzickova, G.; Arslan, D.; Luigi, L. Phenolic profile and in vitro antioxidant power of different milk thistle [Silybum marianum (L.) Gaertn.] cultivars. Ind. Crop. Prod. 2016, 83, 11–16.

^8^ Qin, N.-B.; Jia, C.-C.; Xu, J.; Li, D.-H.; Xu, F.-X.; Bai, J.; Li, Z.-L.; Hua, H.-M. New amides from seeds of Silybum marianum with potential antioxidant and antidiabetic activities. Fitoterapia 2017, 119, 83–89.

^9^ Mhamdi, B.; Abbassi, F.; Smaoui, A.; Abdelly, C.; Marzouk, B. Fatty acids, essential oil and phenolics composition of Silybum marianum seeds and their antioxidant activities. Pak. J. Pharm. Sci. 2016, 29, 953–959.

^10^ Uehara, A.; Nakata, M.; Kitajima, J.; Iwashina, T. Internal and external flavonoids from the leaves of Japanese Chrysanthemum species (Asteraceae). Biochem. Syst. Ecol. 2012, 41, 142–149.

^11^ Lee, D.-K.; Yoon, M.H.; Kang, Y.P.; Yu, J.; Park, J.H.; Lee, J.; Kwon, S.W. Comparison of primary and secondary metabolites for suitability to discriminate the origins of Schisandra chinensis by GC/MS and LC/MS. Food Chem. 2013, 141, 3931–3937.

^12^ Tuli, H.S.; Sandhu, S.S.; Sharma, A.K. Pharmacological and therapeutic potential of cordyceps with special reference to cordycepin. 3 Biotech 2014, 4, 1–12, doi:10.1007/s13205-013-0121-9.

^13^ Xiao, J.-H.; Zhong, J.-J. Secondary Metabolites from Cordyceps Species and Their Antitumor Activity Studies. Recent Patents Biotechnol. 2007, 1, 123–137.

^14^ Mousavi, S.N.M.; Delazar, A.; Nazemiyeh, H.; Khodaie, L. Biological Activity and Phytochemical Study of Scutellaria platystegia. Iran. J. Pharm. Res. 2015, 14, 215–223.

^15^ Gong, P.; Li, Y.; Yao, C.; Guo, H.; Hwang, H.; Liu, X.; Xu, Y.; Wang, X. Traditional Chinese Medicine on the Treatment of Coronary Heart Disease in Recent 20 Years. J. Altern. Complement. Med. 2017, 23, 659–666.

^16^ Chabane, D.; Assani, A.; Mouhoub, F.; Chahinez, B.; Nacer-bey, N. Anatomical, phytochemical and pharmacological studies of roots of Cnicus benedictus L. Int. J. Med. Plant Res. 2013, 2, 204–208.

^17^ Singh, G.; Maurya, S.; De Lampasona, M.P.; Catalán, C. Chemical constituents, antifungal and antioxidative potential of Foeniculum vulgare volatile oil and its acetone extract. Food Control. 2006, 17, 745–752.

^18^ Rather, M.A.; Dar, B.A.; Sofi, S.N.; Bhat, B.A.; Qurishi, M.A. Foeniculum vulgare: A comprehensive review of its traditional use, phytochemistry, pharmacology, and safety. Arab. J. Chem. 2016, 9, S1574–S1583.

^19^ Huber, M.; Triebwasser-Freese, D.; Reichelt, M.; Heiling, S.; Paetz, C.; Chandran, J.N.; Bartram, S.; Schneider, B.; Gershenzon, J.; Erb, M. Identification, quantification, spatiotemporal distribution and genetic variation of major latex secondary metabolites in the common dandelion (Taraxacum officinale agg.). Phytochem 2015, 115, 89–98.

^20^ Fenwick, G.; Lutomski, J.; Nieman, C. Liquorice, Glycyrrhiza glabra L.—Composition, uses and analysis. Food Chem. 1990, 38, 119–143.

**Supplementary Table 2** Characteristics of the bioactive compounds identified by the U-HPLC-HRMS/MS targeted screening.

| **Searched compound^a^** | **Elemental composition** | **Neutral exact mass (m/z)** | **RT (min)** | **Ionization polarity** | **Degree of certainty of compound identification^b^** | **Overview of the samples in which the compounds were detected^c^** | | | | | | | | | | | | | | | | | | | | | | | | | | |
| --- | --- | --- | --- | --- | --- | --- | --- | --- | --- | --- | --- | --- | --- | --- | --- | --- | --- | --- | --- | --- | --- | --- | --- | --- | --- | --- | --- | --- | --- | --- | --- | --- |
|  |  |  |  |  |  | **Silymarin SA** | **1** | **2** | **3** | **4** | **5** | **6** | **7** | **8** | **9** | **10** | **11** | **12** | **13** | **14** | **15** | **16** | **17** | **18** | **19** | **20** | **21** | **22** | **23** | **24** | **25** | **26** |
| **isosilandrin A,B; neosilyhermin A,B; silandrin A,B; silyhermin, silymonin** | C25H22O9 | 466.1264 | 3.42 | POS | 2 |  |  |  |  |  |  |  |  |  |  |  |  |  |  |  |  |  |  |  |  |  |  |  |  |  |  |  |
|  |  |  | 3.72 | POS | 2 |  |  |  |  |  |  |  |  |  |  |  |  |  |  |  |  |  |  |  |  |  |  |  |  |  |  |  |
|  |  |  | 3.82 | POS | 2 |  |  |  |  |  |  |  |  |  |  |  |  |  |  |  |  |  |  |  |  |  |  |  |  |  |  |  |
|  |  |  | 4.45 | POS | 2 |  |  |  |  |  |  |  |  |  |  |  |  |  |  |  |  |  |  |  |  |  |  |  |  |  |  |  |
|  |  |  | 5.13 | NEG | 2 |  |  |  |  |  |  |  |  |  |  |  |  |  |  |  |  |  |  |  |  |  |  |  |  |  |  |  |
|  |  |  | 5.85 | NEG | 2 |  |  |  |  |  |  |  |  |  |  |  |  |  |  |  |  |  |  |  |  |  |  |  |  |  |  |  |
|  |  |  | 6.47 | NEG | 2 |  |  |  |  |  |  |  |  |  |  |  |  |  |  |  |  |  |  |  |  |  |  |  |  |  |  |  |
|  |  |  | 6.57 | NEG | 2 |  |  |  |  |  |  |  |  |  |  |  |  |  |  |  |  |  |  |  |  |  |  |  |  |  |  |  |
|  |  |  | 6.69 | NEG | 2 |  |  |  |  |  |  |  |  |  |  |  |  |  |  |  |  |  |  |  |  |  |  |  |  |  |  |  |
|  |  |  | 6.94 | NEG | 2 |  |  |  |  |  |  |  |  |  |  |  |  |  |  |  |  |  |  |  |  |  |  |  |  |  |  |  |
| **silyamandin** | C25H22O11 | 498.1162 | 3.80 | NEG | 2 |  |  |  |  |  |  |  |  |  |  |  |  |  |  |  |  |  |  |  |  |  |  |  |  |  |  |  |
|  |  |  | 4.80 | POS | 2 |  |  |  |  |  |  |  |  |  |  |  |  |  |  |  |  |  |  |  |  |  |  |  |  |  |  |  |
|  |  |  | 5.16 | NEG | 2 |  |  |  |  |  |  |  |  |  |  |  |  |  |  |  |  |  |  |  |  |  |  |  |  |  |  |  |
| **catechin, epicatechin** | C15H14O6 | 290.0790 | 3.28 | NEG | 1 |  |  |  |  |  |  |  |  |  |  |  |  |  |  |  |  |  |  |  |  |  |  |  |  |  |  |  |
|  |  |  | 3.77 | NEG | 2 |  |  |  |  |  |  |  |  |  |  |  |  |  |  |  |  |  |  |  |  |  |  |  |  |  |  |  |
|  |  |  | 4.79 | NEG | 2 |  |  |  |  |  |  |  |  |  |  |  |  |  |  |  |  |  |  |  |  |  |  |  |  |  |  |  |
|  |  |  | 5.60 | NEG | 2 |  |  |  |  |  |  |  |  |  |  |  |  |  |  |  |  |  |  |  |  |  |  |  |  |  |  |  |
| **naringenin** | C15H12O5 | 272.0685 | 3.77 | NEG | 2 |  |  |  |  |  |  |  |  |  |  |  |  |  |  |  |  |  |  |  |  |  |  |  |  |  |  |  |
|  |  |  | 4.59 | NEG | 2 |  |  |  |  |  |  |  |  |  |  |  |  |  |  |  |  |  |  |  |  |  |  |  |  |  |  |  |
|  |  |  | 5.14 | POS | 2 |  |  |  |  |  |  |  |  |  |  |  |  |  |  |  |  |  |  |  |  |  |  |  |  |  |  |  |
|  |  |  | 5.88 | NEG | 1 |  |  |  |  |  |  |  |  |  |  |  |  |  |  |  |  |  |  |  |  |  |  |  |  |  |  |  |
|  |  |  | 6.09 | NEG | 2 |  |  |  |  |  |  |  |  |  |  |  |  |  |  |  |  |  |  |  |  |  |  |  |  |  |  |  |
|  |  |  | 6.58 | NEG | 2 |  |  |  |  |  |  |  |  |  |  |  |  |  |  |  |  |  |  |  |  |  |  |  |  |  |  |  |
|  |  |  | 7.98 | NEG | 2 |  |  |  |  |  |  |  |  |  |  |  |  |  |  |  |  |  |  |  |  |  |  |  |  |  |  |  |
| **aromadendrin, eriodictyol** | C15H12O6 | 288.0634 | 4.55 | NEG | 2 |  |  |  |  |  |  |  |  |  |  |  |  |  |  |  |  |  |  |  |  |  |  |  |  |  |  |  |
|  |  |  | 5.28 | NEG | 2 |  |  |  |  |  |  |  |  |  |  |  |  |  |  |  |  |  |  |  |  |  |  |  |  |  |  |  |
|  |  |  | 5.65 | POS | 2 |  |  |  |  |  |  |  |  |  |  |  |  |  |  |  |  |  |  |  |  |  |  |  |  |  |  |  |
| **eupatilin** | C18H16O7 | 344.0896 | 4.18 | NEG | 2 |  |  |  |  |  |  |  |  |  |  |  |  |  |  |  |  |  |  |  |  |  |  |  |  |  |  |  |
|  |  |  | 4.92 | NEG | 2 |  |  |  |  |  |  |  |  |  |  |  |  |  |  |  |  |  |  |  |  |  |  |  |  |  |  |  |
|  |  |  | 6.46 | NEG | 2 |  |  |  |  |  |  |  |  |  |  |  |  |  |  |  |  |  |  |  |  |  |  |  |  |  |  |  |
|  |  |  | 6.55 | POS | 2 |  |  |  |  |  |  |  |  |  |  |  |  |  |  |  |  |  |  |  |  |  |  |  |  |  |  |  |
|  |  |  | 6.71 | POS | 2 |  |  |  |  |  |  |  |  |  |  |  |  |  |  |  |  |  |  |  |  |  |  |  |  |  |  |  |
|  |  |  | 7.75 | POS | 2 |  |  |  |  |  |  |  |  |  |  |  |  |  |  |  |  |  |  |  |  |  |  |  |  |  |  |  |
|  |  |  | 8.03 | POS | 2 |  |  |  |  |  |  |  |  |  |  |  |  |  |  |  |  |  |  |  |  |  |  |  |  |  |  |  |
| **hymenoxin** | C19H18O8 | 374.1002 | 6.74 | POS | 2 |  |  |  |  |  |  |  |  |  |  |  |  |  |  |  |  |  |  |  |  |  |  |  |  |  |  |  |
|  |  |  | 6.96 | POS | 2 |  |  |  |  |  |  |  |  |  |  |  |  |  |  |  |  |  |  |  |  |  |  |  |  |  |  |  |
| **jaceosidin** | C17H14O7 | 330.0740 | 4.26 | NEG | 2 |  |  |  |  |  |  |  |  |  |  |  |  |  |  |  |  |  |  |  |  |  |  |  |  |  |  |  |
|  |  |  | 5.32 | POS | 2 |  |  |  |  |  |  |  |  |  |  |  |  |  |  |  |  |  |  |  |  |  |  |  |  |  |  |  |
|  |  |  | 5.45 | NEG | 2 |  |  |  |  |  |  |  |  |  |  |  |  |  |  |  |  |  |  |  |  |  |  |  |  |  |  |  |
|  |  |  | 6.07 | POS | 2 |  |  |  |  |  |  |  |  |  |  |  |  |  |  |  |  |  |  |  |  |  |  |  |  |  |  |  |
|  |  |  | 6.19 | NEG | 2 |  |  |  |  |  |  |  |  |  |  |  |  |  |  |  |  |  |  |  |  |  |  |  |  |  |  |  |
|  |  |  | 6.42 | POS | 2 |  |  |  |  |  |  |  |  |  |  |  |  |  |  |  |  |  |  |  |  |  |  |  |  |  |  |  |
|  |  |  | 6.67 | POS | 2 |  |  |  |  |  |  |  |  |  |  |  |  |  |  |  |  |  |  |  |  |  |  |  |  |  |  |  |
|  |  |  | 6.88 | NEG | 2 |  |  |  |  |  |  |  |  |  |  |  |  |  |  |  |  |  |  |  |  |  |  |  |  |  |  |  |
| **sudachitin** | C18H16O8 | 360.0845 | 4.18 | NEG | 1 |  |  |  |  |  |  |  |  |  |  |  |  |  |  |  |  |  |  |  |  |  |  |  |  |  |  |  |
|  |  |  | 5.66 | POS | 2 |  |  |  |  |  |  |  |  |  |  |  |  |  |  |  |  |  |  |  |  |  |  |  |  |  |  |  |
|  |  |  | 5.75 | NEG | 2 |  |  |  |  |  |  |  |  |  |  |  |  |  |  |  |  |  |  |  |  |  |  |  |  |  |  |  |
| **vitexin, isovitexin, apigenin-7-O-glucoside** | C21H20O10 | 432.1056 | 4.13 | POS | 2 |  |  |  |  |  |  |  |  |  |  |  |  |  |  |  |  |  |  |  |  |  |  |  |  |  |  |  |
|  |  |  | 6.41 | NEG | 2 |  |  |  |  |  |  |  |  |  |  |  |  |  |  |  |  |  |  |  |  |  |  |  |  |  |  |  |
| **luteolin** | C15H10O6 | 286.0477 | 4.01 | NEG | 1 |  |  |  |  |  |  |  |  |  |  |  |  |  |  |  |  |  |  |  |  |  |  |  |  |  |  |  |
|  |  |  | 5.78 | NEG | 1 |  |  |  |  |  |  |  |  |  |  |  |  |  |  |  |  |  |  |  |  |  |  |  |  |  |  |  |
| **kaempferol** | C15H10O6 | 286.0477 | 5.26 | POS | 2 |  |  |  |  |  |  |  |  |  |  |  |  |  |  |  |  |  |  |  |  |  |  |  |  |  |  |  |
|  |  |  | 6.34 | NEG | 2 |  |  |  |  |  |  |  |  |  |  |  |  |  |  |  |  |  |  |  |  |  |  |  |  |  |  |  |
|  |  |  | 6.88 | NEG | 2 |  |  |  |  |  |  |  |  |  |  |  |  |  |  |  |  |  |  |  |  |  |  |  |  |  |  |  |
| **pectolinarigenin, kumatakenin** | C17H14O6 | 314.0790 | 3.69 | NEG | 2 |  |  |  |  |  |  |  |  |  |  |  |  |  |  |  |  |  |  |  |  |  |  |  |  |  |  |  |
|  |  |  | 5.27 | NEG | 2 |  |  |  |  |  |  |  |  |  |  |  |  |  |  |  |  |  |  |  |  |  |  |  |  |  |  |  |
|  |  |  | 5.75 | NEG | 2 |  |  |  |  |  |  |  |  |  |  |  |  |  |  |  |  |  |  |  |  |  |  |  |  |  |  |  |
|  |  |  | 6.71 | POS | 2 |  |  |  |  |  |  |  |  |  |  |  |  |  |  |  |  |  |  |  |  |  |  |  |  |  |  |  |
|  |  |  | 7.10 | NEG | 2 |  |  |  |  |  |  |  |  |  |  |  |  |  |  |  |  |  |  |  |  |  |  |  |  |  |  |  |
|  |  |  | 7.23 | POS | 2 |  |  |  |  |  |  |  |  |  |  |  |  |  |  |  |  |  |  |  |  |  |  |  |  |  |  |  |
|  |  |  | 7.55 | NEG | 2 |  |  |  |  |  |  |  |  |  |  |  |  |  |  |  |  |  |  |  |  |  |  |  |  |  |  |  |
|  |  |  | 5.17 | POS | 2 |  |  |  |  |  |  |  |  |  |  |  |  |  |  |  |  |  |  |  |  |  |  |  |  |  |  |  |
| **apigenin** | C15H10O5 | 270.0528 | 6.35 | POS | 1 |  |  |  |  |  |  |  |  |  |  |  |  |  |  |  |  |  |  |  |  |  |  |  |  |  |  |  |
| **genistein** | C15H10O5 | 270.0528 | 5.63 | NEG | 2 |  |  |  |  |  |  |  |  |  |  |  |  |  |  |  |  |  |  |  |  |  |  |  |  |  |  |  |
|  |  |  | 8.14 | NEG | 2 |  |  |  |  |  |  |  |  |  |  |  |  |  |  |  |  |  |  |  |  |  |  |  |  |  |  |  |
|  |  |  | 3.78 | NEG | 2 |  |  |  |  |  |  |  |  |  |  |  |  |  |  |  |  |  |  |  |  |  |  |  |  |  |  |  |
| **nepetin, rhamentin** | C16H12O7 | 316.0583 | 4.00 | NEG | 2 |  |  |  |  |  |  |  |  |  |  |  |  |  |  |  |  |  |  |  |  |  |  |  |  |  |  |  |
|  |  |  | 4.27 | NEG | 2 |  |  |  |  |  |  |  |  |  |  |  |  |  |  |  |  |  |  |  |  |  |  |  |  |  |  |  |
|  |  |  | 4.80 | POS | 2 |  |  |  |  |  |  |  |  |  |  |  |  |  |  |  |  |  |  |  |  |  |  |  |  |  |  |  |
|  |  |  | 5.63 | NEG | 2 |  |  |  |  |  |  |  |  |  |  |  |  |  |  |  |  |  |  |  |  |  |  |  |  |  |  |  |
|  |  |  | 6.34 | NEG | 2 |  |  |  |  |  |  |  |  |  |  |  |  |  |  |  |  |  |  |  |  |  |  |  |  |  |  |  |
|  |  |  | 6.63 | NEG | 2 |  |  |  |  |  |  |  |  |  |  |  |  |  |  |  |  |  |  |  |  |  |  |  |  |  |  |  |
|  |  |  | 6.74 | POS | 2 |  |  |  |  |  |  |  |  |  |  |  |  |  |  |  |  |  |  |  |  |  |  |  |  |  |  |  |
|  |  |  | 2.98 | POS | 2 |  |  |  |  |  |  |  |  |  |  |  |  |  |  |  |  |  |  |  |  |  |  |  |  |  |  |  |
|  |  |  | 3.88 | NEG | 2 |  |  |  |  |  |  |  |  |  |  |  |  |  |  |  |  |  |  |  |  |  |  |  |  |  |  |  |
|  |  |  | 4.40 | NEG | 2 |  |  |  |  |  |  |  |  |  |  |  |  |  |  |  |  |  |  |  |  |  |  |  |  |  |  |  |
| **orientin, isoorientin, astragalin, cynaroside** | C21H20O11 | 448.1006 | 3.93 | NEG | 2 |  |  |  |  |  |  |  |  |  |  |  |  |  |  |  |  |  |  |  |  |  |  |  |  |  |  |  |
|  |  |  | 4.02 | POS | 2 |  |  |  |  |  |  |  |  |  |  |  |  |  |  |  |  |  |  |  |  |  |  |  |  |  |  |  |
|  |  |  | 4.92 | NEG | 2 |  |  |  |  |  |  |  |  |  |  |  |  |  |  |  |  |  |  |  |  |  |  |  |  |  |  |  |
| **apigenin-5,7-dimethyl ether** | C17H14O5 | 298.0841 | 5.41 | NEG | 2 |  |  |  |  |  |  |  |  |  |  |  |  |  |  |  |  |  |  |  |  |  |  |  |  |  |  |  |
|  |  |  | 5.73 | POS | 2 |  |  |  |  |  |  |  |  |  |  |  |  |  |  |  |  |  |  |  |  |  |  |  |  |  |  |  |
|  |  |  | 6.09 | POS | 2 |  |  |  |  |  |  |  |  |  |  |  |  |  |  |  |  |  |  |  |  |  |  |  |  |  |  |  |
|  |  |  | 6.50 | POS | 2 |  |  |  |  |  |  |  |  |  |  |  |  |  |  |  |  |  |  |  |  |  |  |  |  |  |  |  |
|  |  |  | 6.70 | NEG | 2 |  |  |  |  |  |  |  |  |  |  |  |  |  |  |  |  |  |  |  |  |  |  |  |  |  |  |  |
|  |  |  | 7.76 | NEG | 2 |  |  |  |  |  |  |  |  |  |  |  |  |  |  |  |  |  |  |  |  |  |  |  |  |  |  |  |
|  |  |  | 5.18 | POS | 2 |  |  |  |  |  |  |  |  |  |  |  |  |  |  |  |  |  |  |  |  |  |  |  |  |  |  |  |
|  |  |  | 5.59 | POS | 2 |  |  |  |  |  |  |  |  |  |  |  |  |  |  |  |  |  |  |  |  |  |  |  |  |  |  |  |
|  |  |  | 6.00 | POS | 2 |  |  |  |  |  |  |  |  |  |  |  |  |  |  |  |  |  |  |  |  |  |  |  |  |  |  |  |
| **apigenin-7-O-glucuronide** | C21H18O11 | 446.0849 | 5.78 | POS | 2 |  |  |  |  |  |  |  |  |  |  |  |  |  |  |  |  |  |  |  |  |  |  |  |  |  |  |  |
|  |  |  | 4.26 | NEG | 1 |  |  |  |  |  |  |  |  |  |  |  |  |  |  |  |  |  |  |  |  |  |  |  |  |  |  |  |
|  |  |  | 4.67 | NEG | 2 |  |  |  |  |  |  |  |  |  |  |  |  |  |  |  |  |  |  |  |  |  |  |  |  |  |  |  |
| **isokaempferide-7-rhamnoside** | C22H22O10 | 446.1213 | 8.79 | POS | 2 |  |  |  |  |  |  |  |  |  |  |  |  |  |  |  |  |  |  |  |  |  |  |  |  |  |  |  |
| **rutin** | C27H30O16 | 610.1534 | 4.21 | NEG | 2 |  |  |  |  |  |  |  |  |  |  |  |  |  |  |  |  |  |  |  |  |  |  |  |  |  |  |  |
| **apigenin-4,7-diglucoside, kaempferol-3-rutinoside** | C27H30O15 | 594.1585 | 4.69 | NEG | 2 |  |  |  |  |  |  |  |  |  |  |  |  |  |  |  |  |  |  |  |  |  |  |  |  |  |  |  |
|  |  |  | 5.96 | NEG | 2 |  |  |  |  |  |  |  |  |  |  |  |  |  |  |  |  |  |  |  |  |  |  |  |  |  |  |  |
| **miquelianin** | C21H18O13 | 478.0747 | 6.79 | POS | 2 |  |  |  |  |  |  |  |  |  |  |  |  |  |  |  |  |  |  |  |  |  |  |  |  |  |  |  |
| **patuletin** | C16H12O8 | 332.0532 | 3.14 | POS | 2 |  |  |  |  |  |  |  |  |  |  |  |  |  |  |  |  |  |  |  |  |  |  |  |  |  |  |  |
|  |  |  | 3.69 | NEG | 1 |  |  |  |  |  |  |  |  |  |  |  |  |  |  |  |  |  |  |  |  |  |  |  |  |  |  |  |
|  |  |  | 3.75 | NEG | 2 |  |  |  |  |  |  |  |  |  |  |  |  |  |  |  |  |  |  |  |  |  |  |  |  |  |  |  |
| **quercetin** | C15H10O7 | 302.0427 | 3.99 | POS | 1 |  |  |  |  |  |  |  |  |  |  |  |  |  |  |  |  |  |  |  |  |  |  |  |  |  |  |  |
|  |  |  | 4.30 | POS | 2 |  |  |  |  |  |  |  |  |  |  |  |  |  |  |  |  |  |  |  |  |  |  |  |  |  |  |  |
|  |  |  | 5.61 | NEG | 2 |  |  |  |  |  |  |  |  |  |  |  |  |  |  |  |  |  |  |  |  |  |  |  |  |  |  |  |
|  |  |  | 5.93 | NEG | 1 |  |  |  |  |  |  |  |  |  |  |  |  |  |  |  |  |  |  |  |  |  |  |  |  |  |  |  |
|  |  |  | 6.81 | NEG | 2 |  |  |  |  |  |  |  |  |  |  |  |  |  |  |  |  |  |  |  |  |  |  |  |  |  |  |  |
|  |  |  | 1.80 | POS | 2 |  |  |  |  |  |  |  |  |  |  |  |  |  |  |  |  |  |  |  |  |  |  |  |  |  |  |  |
|  |  |  | 2.88 | POS | 2 |  |  |  |  |  |  |  |  |  |  |  |  |  |  |  |  |  |  |  |  |  |  |  |  |  |  |  |
|  |  |  | 2.96 | POS | 2 |  |  |  |  |  |  |  |  |  |  |  |  |  |  |  |  |  |  |  |  |  |  |  |  |  |  |  |
| **coniferylaldehyd** | C10H10O3 | 178.0630 | 3.56 | NEG | 2 |  |  |  |  |  |  |  |  |  |  |  |  |  |  |  |  |  |  |  |  |  |  |  |  |  |  |  |
|  |  |  | 4.30 | POS | 2 |  |  |  |  |  |  |  |  |  |  |  |  |  |  |  |  |  |  |  |  |  |  |  |  |  |  |  |
|  |  |  | 4.87 | POS | 2 |  |  |  |  |  |  |  |  |  |  |  |  |  |  |  |  |  |  |  |  |  |  |  |  |  |  |  |
|  |  |  | 3.21 | NEG | 2 |  |  |  |  |  |  |  |  |  |  |  |  |  |  |  |  |  |  |  |  |  |  |  |  |  |  |  |
|  |  |  | 3.56 | NEG | 2 |  |  |  |  |  |  |  |  |  |  |  |  |  |  |  |  |  |  |  |  |  |  |  |  |  |  |  |
|  |  |  | 4.52 | POS | 2 |  |  |  |  |  |  |  |  |  |  |  |  |  |  |  |  |  |  |  |  |  |  |  |  |  |  |  |
| **dihydroconiferyl alcohol** | C10H14O3 | 182.0943 | 1.67 | NEG | 2 |  |  |  |  |  |  |  |  |  |  |  |  |  |  |  |  |  |  |  |  |  |  |  |  |  |  |  |
|  |  |  | 3.03 | NEG | 2 |  |  |  |  |  |  |  |  |  |  |  |  |  |  |  |  |  |  |  |  |  |  |  |  |  |  |  |
|  |  |  | 3.20 | NEG | 2 |  |  |  |  |  |  |  |  |  |  |  |  |  |  |  |  |  |  |  |  |  |  |  |  |  |  |  |
| **syringaldehyde** | C9H10O4 | 182.0579 | 3.79 | POS | 2 |  |  |  |  |  |  |  |  |  |  |  |  |  |  |  |  |  |  |  |  |  |  |  |  |  |  |  |
|  |  |  | 5.01 | NEG | 2 |  |  |  |  |  |  |  |  |  |  |  |  |  |  |  |  |  |  |  |  |  |  |  |  |  |  |  |
|  |  |  | 5.95 | POS | 2 |  |  |  |  |  |  |  |  |  |  |  |  |  |  |  |  |  |  |  |  |  |  |  |  |  |  |  |
|  |  |  | 10.20 | POS | 2 |  |  |  |  |  |  |  |  |  |  |  |  |  |  |  |  |  |  |  |  |  |  |  |  |  |  |  |
|  |  |  | 1.65 | POS | 2 |  |  |  |  |  |  |  |  |  |  |  |  |  |  |  |  |  |  |  |  |  |  |  |  |  |  |  |
|  |  |  | 3.25 | NEG | 2 |  |  |  |  |  |  |  |  |  |  |  |  |  |  |  |  |  |  |  |  |  |  |  |  |  |  |  |
|  |  |  | 3.46 | NEG | 2 |  |  |  |  |  |  |  |  |  |  |  |  |  |  |  |  |  |  |  |  |  |  |  |  |  |  |  |
| **ethyl caffeate, methyl ferulate** | C11H12O4 | 208.0736 | 3.97 | NEG | 2 |  |  |  |  |  |  |  |  |  |  |  |  |  |  |  |  |  |  |  |  |  |  |  |  |  |  |  |
|  |  |  | 4.22 | POS | 2 |  |  |  |  |  |  |  |  |  |  |  |  |  |  |  |  |  |  |  |  |  |  |  |  |  |  |  |
|  |  |  | 4.87 | NEG | 2 |  |  |  |  |  |  |  |  |  |  |  |  |  |  |  |  |  |  |  |  |  |  |  |  |  |  |  |
|  |  |  | 5.30 | NEG | 2 |  |  |  |  |  |  |  |  |  |  |  |  |  |  |  |  |  |  |  |  |  |  |  |  |  |  |  |
|  |  |  | 5.71 | NEG | 2 |  |  |  |  |  |  |  |  |  |  |  |  |  |  |  |  |  |  |  |  |  |  |  |  |  |  |  |
|  |  |  | 5.89 | POS | 2 |  |  |  |  |  |  |  |  |  |  |  |  |  |  |  |  |  |  |  |  |  |  |  |  |  |  |  |
|  |  |  | 5.97 | NEG | 2 |  |  |  |  |  |  |  |  |  |  |  |  |  |  |  |  |  |  |  |  |  |  |  |  |  |  |  |
|  |  |  | 7.09 | POS | 2 |  |  |  |  |  |  |  |  |  |  |  |  |  |  |  |  |  |  |  |  |  |  |  |  |  |  |  |
|  |  |  | 4.27 | NEG | 2 |  |  |  |  |  |  |  |  |  |  |  |  |  |  |  |  |  |  |  |  |  |  |  |  |  |  |  |
|  |  |  | 4.59 | NEG | 2 |  |  |  |  |  |  |  |  |  |  |  |  |  |  |  |  |  |  |  |  |  |  |  |  |  |  |  |
|  |  |  | 1.65 | NEG | 2 |  |  |  |  |  |  |  |  |  |  |  |  |  |  |  |  |  |  |  |  |  |  |  |  |  |  |  |
| **1,5-O- dicaffeoylquinic acid, 3,5-O- dicaffeoylquinic acid, 4,5-O-dicaffeoylquinic acid, cynarin** | C25H24O12 | 516.1268 | 3.19 | NEG | 2 |  |  |  |  |  |  |  |  |  |  |  |  |  |  |  |  |  |  |  |  |  |  |  |  |  |  |  |
|  |  |  | 3.61 | NEG | 2 |  |  |  |  |  |  |  |  |  |  |  |  |  |  |  |  |  |  |  |  |  |  |  |  |  |  |  |
| **5-O-feruloylquinic acid** | C17H20O9 | 368.1107 | 3.29 | NEG | 1 |  |  |  |  |  |  |  |  |  |  |  |  |  |  |  |  |  |  |  |  |  |  |  |  |  |  |  |
|  |  |  | 3.36 | POS | 2 |  |  |  |  |  |  |  |  |  |  |  |  |  |  |  |  |  |  |  |  |  |  |  |  |  |  |  |
|  |  |  | 4.95 | POS | 2 |  |  |  |  |  |  |  |  |  |  |  |  |  |  |  |  |  |  |  |  |  |  |  |  |  |  |  |
| **caffeic acid** | C9H8O4 | 180.0423 | 8.69 | POS | 2 |  |  |  |  |  |  |  |  |  |  |  |  |  |  |  |  |  |  |  |  |  |  |  |  |  |  |  |
|  |  |  | 1.73 | POS | 2 |  |  |  |  |  |  |  |  |  |  |  |  |  |  |  |  |  |  |  |  |  |  |  |  |  |  |  |
|  |  |  | 3.64 | NEG | 1 |  |  |  |  |  |  |  |  |  |  |  |  |  |  |  |  |  |  |  |  |  |  |  |  |  |  |  |
|  |  |  | 6.94 | NEG | 2 |  |  |  |  |  |  |  |  |  |  |  |  |  |  |  |  |  |  |  |  |  |  |  |  |  |  |  |
| **coumaric acid** | C9H8O3 | 164.0473 | 5.37 | NEG | 1 |  |  |  |  |  |  |  |  |  |  |  |  |  |  |  |  |  |  |  |  |  |  |  |  |  |  |  |
|  |  |  | 3.57 | NEG | 1 |  |  |  |  |  |  |  |  |  |  |  |  |  |  |  |  |  |  |  |  |  |  |  |  |  |  |  |
|  |  |  | 3.20 | NEG | 1 |  |  |  |  |  |  |  |  |  |  |  |  |  |  |  |  |  |  |  |  |  |  |  |  |  |  |  |
|  |  |  | 3.11 | POS | 2 |  |  |  |  |  |  |  |  |  |  |  |  |  |  |  |  |  |  |  |  |  |  |  |  |  |  |  |
| **dihydroxybenzoic acid** | C7H6O4 | 154.0266 | 3.24 | NEG | 2 |  |  |  |  |  |  |  |  |  |  |  |  |  |  |  |  |  |  |  |  |  |  |  |  |  |  |  |
|  |  |  | 4.10 | NEG | 2 |  |  |  |  |  |  |  |  |  |  |  |  |  |  |  |  |  |  |  |  |  |  |  |  |  |  |  |
| **ferulic acid** | C10H10O4 | 194.0579 | 4.94 | NEG | 2 |  |  |  |  |  |  |  |  |  |  |  |  |  |  |  |  |  |  |  |  |  |  |  |  |  |  |  |
|  |  |  | 5.51 | POS | 2 |  |  |  |  |  |  |  |  |  |  |  |  |  |  |  |  |  |  |  |  |  |  |  |  |  |  |  |
|  |  |  | 1.33 | NEG | 1 |  |  |  |  |  |  |  |  |  |  |  |  |  |  |  |  |  |  |  |  |  |  |  |  |  |  |  |
|  |  |  | 2.37 | POS | 2 |  |  |  |  |  |  |  |  |  |  |  |  |  |  |  |  |  |  |  |  |  |  |  |  |  |  |  |
|  |  |  | 2.98 | POS | 2 |  |  |  |  |  |  |  |  |  |  |  |  |  |  |  |  |  |  |  |  |  |  |  |  |  |  |  |
| **gallic acid** | C7H6O5 | 170.0215 | 4.05 | NEG | 2 |  |  |  |  |  |  |  |  |  |  |  |  |  |  |  |  |  |  |  |  |  |  |  |  |  |  |  |
|  |  |  | 4.74 | POS | 2 |  |  |  |  |  |  |  |  |  |  |  |  |  |  |  |  |  |  |  |  |  |  |  |  |  |  |  |
|  |  |  | 2.99 | NEG | 2 |  |  |  |  |  |  |  |  |  |  |  |  |  |  |  |  |  |  |  |  |  |  |  |  |  |  |  |
|  |  |  | 4.13 | POS | 2 |  |  |  |  |  |  |  |  |  |  |  |  |  |  |  |  |  |  |  |  |  |  |  |  |  |  |  |
|  |  |  | 5.92 | POS | 2 |  |  |  |  |  |  |  |  |  |  |  |  |  |  |  |  |  |  |  |  |  |  |  |  |  |  |  |
| **guaiacol** | C7H8O2 | 124.0524 | 1.88 | NEG | 2 |  |  |  |  |  |  |  |  |  |  |  |  |  |  |  |  |  |  |  |  |  |  |  |  |  |  |  |
|  |  |  | 3.08 | NEG | 1 |  |  |  |  |  |  |  |  |  |  |  |  |  |  |  |  |  |  |  |  |  |  |  |  |  |  |  |
|  |  |  | 3.88 | NEG | 2 |  |  |  |  |  |  |  |  |  |  |  |  |  |  |  |  |  |  |  |  |  |  |  |  |  |  |  |
| **chlorogenic acid** | C16H18O9 | 354.0951 | 1.65 | POS | 2 |  |  |  |  |  |  |  |  |  |  |  |  |  |  |  |  |  |  |  |  |  |  |  |  |  |  |  |
|  |  |  | 2.75 | NEG | 1 |  |  |  |  |  |  |  |  |  |  |  |  |  |  |  |  |  |  |  |  |  |  |  |  |  |  |  |
|  |  |  | 3.00 | NEG | 2 |  |  |  |  |  |  |  |  |  |  |  |  |  |  |  |  |  |  |  |  |  |  |  |  |  |  |  |
| **salicylic acid** | C7H6O3 | 138.0317 | 3.19 | NEG | 2 |  |  |  |  |  |  |  |  |  |  |  |  |  |  |  |  |  |  |  |  |  |  |  |  |  |  |  |
|  |  |  | 3.90 | NEG | 2 |  |  |  |  |  |  |  |  |  |  |  |  |  |  |  |  |  |  |  |  |  |  |  |  |  |  |  |
|  |  |  | 4.55 | NEG | 2 |  |  |  |  |  |  |  |  |  |  |  |  |  |  |  |  |  |  |  |  |  |  |  |  |  |  |  |
|  |  |  | 3.56 | NEG | 2 |  |  |  |  |  |  |  |  |  |  |  |  |  |  |  |  |  |  |  |  |  |  |  |  |  |  |  |
|  |  |  | 4.12 | POS | 2 |  |  |  |  |  |  |  |  |  |  |  |  |  |  |  |  |  |  |  |  |  |  |  |  |  |  |  |
|  |  |  | 4.50 | NEG | 2 |  |  |  |  |  |  |  |  |  |  |  |  |  |  |  |  |  |  |  |  |  |  |  |  |  |  |  |
| **sinapinic acid** | C11H12O5 | 224.0685 | 1.64 | NEG | 2 |  |  |  |  |  |  |  |  |  |  |  |  |  |  |  |  |  |  |  |  |  |  |  |  |  |  |  |
|  |  |  | 2.86 | POS | 2 |  |  |  |  |  |  |  |  |  |  |  |  |  |  |  |  |  |  |  |  |  |  |  |  |  |  |  |
|  |  |  | 4.03 | NEG | 2 |  |  |  |  |  |  |  |  |  |  |  |  |  |  |  |  |  |  |  |  |  |  |  |  |  |  |  |
| **syringic acid** | C9H10O5 | 198.0528 | 1.48 | POS | 2 |  |  |  |  |  |  |  |  |  |  |  |  |  |  |  |  |  |  |  |  |  |  |  |  |  |  |  |
|  |  |  | 1.65 | NEG | 1 |  |  |  |  |  |  |  |  |  |  |  |  |  |  |  |  |  |  |  |  |  |  |  |  |  |  |  |
|  |  |  | 3.02 | POS | 2 |  |  |  |  |  |  |  |  |  |  |  |  |  |  |  |  |  |  |  |  |  |  |  |  |  |  |  |
| **vanillic acid** | C8H8O4 | 168.0423 | 3.16 | POS | 2 |  |  |  |  |  |  |  |  |  |  |  |  |  |  |  |  |  |  |  |  |  |  |  |  |  |  |  |
|  |  |  | 3.34 | POS | 2 |  |  |  |  |  |  |  |  |  |  |  |  |  |  |  |  |  |  |  |  |  |  |  |  |  |  |  |
|  |  |  | 4.69 | POS | 2 |  |  |  |  |  |  |  |  |  |  |  |  |  |  |  |  |  |  |  |  |  |  |  |  |  |  |  |
|  |  |  | 4.89 | POS | 2 |  |  |  |  |  |  |  |  |  |  |  |  |  |  |  |  |  |  |  |  |  |  |  |  |  |  |  |
|  |  |  | 5.05 | POS | 2 |  |  |  |  |  |  |  |  |  |  |  |  |  |  |  |  |  |  |  |  |  |  |  |  |  |  |  |
| **mariamide A** | C42H46N4O10 | 766.3214 | 5.24 | POS | 2 |  |  |  |  |  |  |  |  |  |  |  |  |  |  |  |  |  |  |  |  |  |  |  |  |  |  |  |
|  |  |  | 5.36 | POS | 2 |  |  |  |  |  |  |  |  |  |  |  |  |  |  |  |  |  |  |  |  |  |  |  |  |  |  |  |
|  |  |  | 5.45 | POS | 2 |  |  |  |  |  |  |  |  |  |  |  |  |  |  |  |  |  |  |  |  |  |  |  |  |  |  |  |
|  |  |  | 5.69 | POS | 2 |  |  |  |  |  |  |  |  |  |  |  |  |  |  |  |  |  |  |  |  |  |  |  |  |  |  |  |
|  |  |  | 6.04 | POS | 2 |  |  |  |  |  |  |  |  |  |  |  |  |  |  |  |  |  |  |  |  |  |  |  |  |  |  |  |
|  |  |  | 14.82 | POS | 2 |  |  |  |  |  |  |  |  |  |  |  |  |  |  |  |  |  |  |  |  |  |  |  |  |  |  |  |
|  |  |  | 4.32 | POS | 2 |  |  |  |  |  |  |  |  |  |  |  |  |  |  |  |  |  |  |  |  |  |  |  |  |  |  |  |
|  |  |  | 4.65 | POS | 2 |  |  |  |  |  |  |  |  |  |  |  |  |  |  |  |  |  |  |  |  |  |  |  |  |  |  |  |
|  |  |  | 5.00 | POS | 2 |  |  |  |  |  |  |  |  |  |  |  |  |  |  |  |  |  |  |  |  |  |  |  |  |  |  |  |
| **mariamide B** | C21H24N2O5 | 384.1685 | 3.53 | POS | 2 |  |  |  |  |  |  |  |  |  |  |  |  |  |  |  |  |  |  |  |  |  |  |  |  |  |  |  |
|  |  |  | 3.34 | POS | 2 |  |  |  |  |  |  |  |  |  |  |  |  |  |  |  |  |  |  |  |  |  |  |  |  |  |  |  |
|  |  |  | 7.71 | POS | 2 |  |  |  |  |  |  |  |  |  |  |  |  |  |  |  |  |  |  |  |  |  |  |  |  |  |  |  |
| **3- methylcarboxymethyl-indole-1-N-beta-D-glucopyranoside** | C16H19NO7 | 337.1162 | 8.00 | POS | 2 |  |  |  |  |  |  |  |  |  |  |  |  |  |  |  |  |  |  |  |  |  |  |  |  |  |  |  |
| **naphthaquinone** | C10 H6 O2 | 158.0368 | 9.09 | NEG | 2 |  |  |  |  |  |  |  |  |  |  |  |  |  |  |  |  |  |  |  |  |  |  |  |  |  |  |  |
| **angeloylgomisin H, propinquanin F** | C28 H36 O8 | 500.2410 | 7.97 | POS | 2 |  |  |  |  |  |  |  |  |  |  |  |  |  |  |  |  |  |  |  |  |  |  |  |  |  |  |  |
|  |  |  | 8.15 | POS | 2 |  |  |  |  |  |  |  |  |  |  |  |  |  |  |  |  |  |  |  |  |  |  |  |  |  |  |  |
|  |  |  | 8.36 | POS | 2 |  |  |  |  |  |  |  |  |  |  |  |  |  |  |  |  |  |  |  |  |  |  |  |  |  |  |  |
| **angeloylgomisin P, gomisin B, gomisin F, schisantherin C, tigloylgomisin P** | C28 H34 O9 | 514.2203 | 8.46 | POS | 2 |  |  |  |  |  |  |  |  |  |  |  |  |  |  |  |  |  |  |  |  |  |  |  |  |  |  |  |
|  |  |  | 8.59 | POS | 2 |  |  |  |  |  |  |  |  |  |  |  |  |  |  |  |  |  |  |  |  |  |  |  |  |  |  |  |
|  |  |  | 8.79 | POS | 2 |  |  |  |  |  |  |  |  |  |  |  |  |  |  |  |  |  |  |  |  |  |  |  |  |  |  |  |
|  |  |  | 9.11 | POS | 2 |  |  |  |  |  |  |  |  |  |  |  |  |  |  |  |  |  |  |  |  |  |  |  |  |  |  |  |
|  |  |  | 7.86 | POS | 2 |  |  |  |  |  |  |  |  |  |  |  |  |  |  |  |  |  |  |  |  |  |  |  |  |  |  |  |
|  |  |  | 8.06 | POS | 2 |  |  |  |  |  |  |  |  |  |  |  |  |  |  |  |  |  |  |  |  |  |  |  |  |  |  |  |
|  |  |  | 7.71 | POS | 2 |  |  |  |  |  |  |  |  |  |  |  |  |  |  |  |  |  |  |  |  |  |  |  |  |  |  |  |
| **angeloylgomisin Q** | C29 H38 O9 | 530.2516 | 8.00 | POS | 2 |  |  |  |  |  |  |  |  |  |  |  |  |  |  |  |  |  |  |  |  |  |  |  |  |  |  |  |
|  |  |  | 7.76 | POS | 2 |  |  |  |  |  |  |  |  |  |  |  |  |  |  |  |  |  |  |  |  |  |  |  |  |  |  |  |
| **benzoylgomisin H** | C30 H34 O8 | 522.2254 | 8.44 | POS | 2 |  |  |  |  |  |  |  |  |  |  |  |  |  |  |  |  |  |  |  |  |  |  |  |  |  |  |  |
|  |  |  | 9.09 | NEG | 2 |  |  |  |  |  |  |  |  |  |  |  |  |  |  |  |  |  |  |  |  |  |  |  |  |  |  |  |
| **gomisin A** | C23 H28 O7 | 416.1835 | 6.69 | POS | 2 |  |  |  |  |  |  |  |  |  |  |  |  |  |  |  |  |  |  |  |  |  |  |  |  |  |  |  |
|  |  |  | 7.34 | POS | 2 |  |  |  |  |  |  |  |  |  |  |  |  |  |  |  |  |  |  |  |  |  |  |  |  |  |  |  |
|  |  |  | 8.07 | POS | 2 |  |  |  |  |  |  |  |  |  |  |  |  |  |  |  |  |  |  |  |  |  |  |  |  |  |  |  |
| **gomisin D** | C28 H34 O10 | 530.2152 | 8.64 | POS | 2 |  |  |  |  |  |  |  |  |  |  |  |  |  |  |  |  |  |  |  |  |  |  |  |  |  |  |  |
|  |  |  | 7.90 | POS | 2 |  |  |  |  |  |  |  |  |  |  |  |  |  |  |  |  |  |  |  |  |  |  |  |  |  |  |  |
| **gomisin G, schisantherin A** | C30 H32 O9 | 536.2046 | 8.27 | POS | 2 |  |  |  |  |  |  |  |  |  |  |  |  |  |  |  |  |  |  |  |  |  |  |  |  |  |  |  |
|  |  |  | 8.37 | NEG | 2 |  |  |  |  |  |  |  |  |  |  |  |  |  |  |  |  |  |  |  |  |  |  |  |  |  |  |  |
| **gomisin J** | C22 H28 O6 | 388.1886 | 8.53 | POS | 2 |  |  |  |  |  |  |  |  |  |  |  |  |  |  |  |  |  |  |  |  |  |  |  |  |  |  |  |
|  |  |  | 8.81 | POS | 2 |  |  |  |  |  |  |  |  |  |  |  |  |  |  |  |  |  |  |  |  |  |  |  |  |  |  |  |
|  |  |  | 8.98 | POS | 2 |  |  |  |  |  |  |  |  |  |  |  |  |  |  |  |  |  |  |  |  |  |  |  |  |  |  |  |
|  |  |  | 9.19 | POS | 2 |  |  |  |  |  |  |  |  |  |  |  |  |  |  |  |  |  |  |  |  |  |  |  |  |  |  |  |
| **gomisin K1, K2, K3** | C23 H30 O6 | 402.2042 | 9.26 | POS | 2 |  |  |  |  |  |  |  |  |  |  |  |  |  |  |  |  |  |  |  |  |  |  |  |  |  |  |  |
|  |  |  | 9.46 | POS | 2 |  |  |  |  |  |  |  |  |  |  |  |  |  |  |  |  |  |  |  |  |  |  |  |  |  |  |  |
| **gomisin M1, M2, L1, L2** | C22 H26 O6 | 386.1729 | 9.57 | POS | 2 |  |  |  |  |  |  |  |  |  |  |  |  |  |  |  |  |  |  |  |  |  |  |  |  |  |  |  |
|  |  |  | 8.01 | POS | 2 |  |  |  |  |  |  |  |  |  |  |  |  |  |  |  |  |  |  |  |  |  |  |  |  |  |  |  |
|  |  |  | 8.20 | POS | 2 |  |  |  |  |  |  |  |  |  |  |  |  |  |  |  |  |  |  |  |  |  |  |  |  |  |  |  |
|  |  |  | 10.17 | POS | 2 |  |  |  |  |  |  |  |  |  |  |  |  |  |  |  |  |  |  |  |  |  |  |  |  |  |  |  |
| **gomisin N, schisandrin B** | C23 H28 O6 | 400.1886 | 10.27 | POS | 2 |  |  |  |  |  |  |  |  |  |  |  |  |  |  |  |  |  |  |  |  |  |  |  |  |  |  |  |
|  |  |  | 9.52 | POS | 2 |  |  |  |  |  |  |  |  |  |  |  |  |  |  |  |  |  |  |  |  |  |  |  |  |  |  |  |
|  |  |  | 9.11 | NEG | 2 |  |  |  |  |  |  |  |  |  |  |  |  |  |  |  |  |  |  |  |  |  |  |  |  |  |  |  |
|  |  |  | 10.85 | POS | 2 |  |  |  |  |  |  |  |  |  |  |  |  |  |  |  |  |  |  |  |  |  |  |  |  |  |  |  |
| **schisandrin A** | C24 H32 O6 | 416.2199 | 6.67 | POS | 2 |  |  |  |  |  |  |  |  |  |  |  |  |  |  |  |  |  |  |  |  |  |  |  |  |  |  |  |
| **schisandrin C** | C22 H24 O6 | 384.1573 | 7.06 | POS | 2 |  |  |  |  |  |  |  |  |  |  |  |  |  |  |  |  |  |  |  |  |  |  |  |  |  |  |  |
|  |  |  | 7.76 | POS | 2 |  |  |  |  |  |  |  |  |  |  |  |  |  |  |  |  |  |  |  |  |  |  |  |  |  |  |  |
| **schisandrin, isoschisandrin** | C24 H32 O7 | 432.2148 | 5.62 | POS | 2 |  |  |  |  |  |  |  |  |  |  |  |  |  |  |  |  |  |  |  |  |  |  |  |  |  |  |  |
|  |  |  | 5.95 | POS | 2 |  |  |  |  |  |  |  |  |  |  |  |  |  |  |  |  |  |  |  |  |  |  |  |  |  |  |  |
|  |  |  | 6.31 | POS | 2 |  |  |  |  |  |  |  |  |  |  |  |  |  |  |  |  |  |  |  |  |  |  |  |  |  |  |  |
| **trachelogenin** | C21 H24 O7 | 388.1522 | 5.84 | POS | 2 |  |  |  |  |  |  |  |  |  |  |  |  |  |  |  |  |  |  |  |  |  |  |  |  |  |  |  |
|  |  |  | 4.10 | POS | 2 |  |  |  |  |  |  |  |  |  |  |  |  |  |  |  |  |  |  |  |  |  |  |  |  |  |  |  |
|  |  |  | 3.96 | POS | 2 |  |  |  |  |  |  |  |  |  |  |  |  |  |  |  |  |  |  |  |  |  |  |  |  |  |  |  |
| **arctigenin** | C21 H24 O6 | 372.1573 | 4.18 | NEG | 2 |  |  |  |  |  |  |  |  |  |  |  |  |  |  |  |  |  |  |  |  |  |  |  |  |  |  |  |
| **nortracheloside** | C26 H32 O12 | 536.1894 | 5.74 | POS | 2 |  |  |  |  |  |  |  |  |  |  |  |  |  |  |  |  |  |  |  |  |  |  |  |  |  |  |  |
| **rosmarinic acid** | C18 H16 O8 | 360.0845 | 5.98 | POS | 2 |  |  |  |  |  |  |  |  |  |  |  |  |  |  |  |  |  |  |  |  |  |  |  |  |  |  |  |
|  |  |  | 7.10 | NEG | 2 |  |  |  |  |  |  |  |  |  |  |  |  |  |  |  |  |  |  |  |  |  |  |  |  |  |  |  |
|  |  |  | 0.73 | NEG | 2 |  |  |  |  |  |  |  |  |  |  |  |  |  |  |  |  |  |  |  |  |  |  |  |  |  |  |  |
|  |  |  | 5.19 | POS | 1 |  |  |  |  |  |  |  |  |  |  |  |  |  |  |  |  |  |  |  |  |  |  |  |  |  |  |  |
|  |  |  | 5.40 | POS | 2 |  |  |  |  |  |  |  |  |  |  |  |  |  |  |  |  |  |  |  |  |  |  |  |  |  |  |  |
| **cordycepic acid** | C6 H14 O6 | 182.0790 | 5.63 | NEG | 2 |  |  |  |  |  |  |  |  |  |  |  |  |  |  |  |  |  |  |  |  |  |  |  |  |  |  |  |
| **baicalein** | C15 H10 O5 | 270.0528 | 6.39 | NEG | 2 |  |  |  |  |  |  |  |  |  |  |  |  |  |  |  |  |  |  |  |  |  |  |  |  |  |  |  |
|  |  |  | 6.73 | POS | 2 |  |  |  |  |  |  |  |  |  |  |  |  |  |  |  |  |  |  |  |  |  |  |  |  |  |  |  |
|  |  |  | 6.96 | POS | 2 |  |  |  |  |  |  |  |  |  |  |  |  |  |  |  |  |  |  |  |  |  |  |  |  |  |  |  |
|  |  |  | 5.37 | NEG | 2 |  |  |  |  |  |  |  |  |  |  |  |  |  |  |  |  |  |  |  |  |  |  |  |  |  |  |  |
| **neobaicalein** | C19 H18 O8 | 374.1002 | 5.75 | NEG | 2 |  |  |  |  |  |  |  |  |  |  |  |  |  |  |  |  |  |  |  |  |  |  |  |  |  |  |  |
|  |  |  | 5.97 | NEG | 2 |  |  |  |  |  |  |  |  |  |  |  |  |  |  |  |  |  |  |  |  |  |  |  |  |  |  |  |
| **skullcapflavone I** | C17 H14 O6 | 314.0790 | 7.10 | NEG | 2 |  |  |  |  |  |  |  |  |  |  |  |  |  |  |  |  |  |  |  |  |  |  |  |  |  |  |  |
|  |  |  | 7.23 | POS | 2 |  |  |  |  |  |  |  |  |  |  |  |  |  |  |  |  |  |  |  |  |  |  |  |  |  |  |  |
|  |  |  | 7.55 | NEG | 2 |  |  |  |  |  |  |  |  |  |  |  |  |  |  |  |  |  |  |  |  |  |  |  |  |  |  |  |
|  |  |  | 5.41 | POS | 2 |  |  |  |  |  |  |  |  |  |  |  |  |  |  |  |  |  |  |  |  |  |  |  |  |  |  |  |
|  |  |  | 7.38 | POS | 2 |  |  |  |  |  |  |  |  |  |  |  |  |  |  |  |  |  |  |  |  |  |  |  |  |  |  |  |
|  |  |  | 7.57 | POS | 2 |  |  |  |  |  |  |  |  |  |  |  |  |  |  |  |  |  |  |  |  |  |  |  |  |  |  |  |
| **oroxylin A, wogonin** | C16 H12 O5 | 284.0685 | 5.22 | NEG | 1 |  |  |  |  |  |  |  |  |  |  |  |  |  |  |  |  |  |  |  |  |  |  |  |  |  |  |  |
|  |  |  | 5.58 | NEG | 1 |  |  |  |  |  |  |  |  |  |  |  |  |  |  |  |  |  |  |  |  |  |  |  |  |  |  |  |
|  |  |  | 5.86 | POS | 2 |  |  |  |  |  |  |  |  |  |  |  |  |  |  |  |  |  |  |  |  |  |  |  |  |  |  |  |
| **baicalin** | C21 H18 O11 | 446.0849 | 5.93 | NEG | 2 |  |  |  |  |  |  |  |  |  |  |  |  |  |  |  |  |  |  |  |  |  |  |  |  |  |  |  |
|  |  |  | 5.68 | POS | 2 |  |  |  |  |  |  |  |  |  |  |  |  |  |  |  |  |  |  |  |  |  |  |  |  |  |  |  |
|  |  |  | 5.86 | POS | 2 |  |  |  |  |  |  |  |  |  |  |  |  |  |  |  |  |  |  |  |  |  |  |  |  |  |  |  |
|  |  |  | 6.13 | POS | 2 |  |  |  |  |  |  |  |  |  |  |  |  |  |  |  |  |  |  |  |  |  |  |  |  |  |  |  |
| **oroxylin A glucoronide, wogonoside** | C22 H20 O11 | 460.1006 | 6.70 | POS | 1 |  |  |  |  |  |  |  |  |  |  |  |  |  |  |  |  |  |  |  |  |  |  |  |  |  |  |  |
|  |  |  | 9.12 | NEG | 2 |  |  |  |  |  |  |  |  |  |  |  |  |  |  |  |  |  |  |  |  |  |  |  |  |  |  |  |
| **skullcapflavone I 2-O-glucoside** | C23 H24 O11 | 476.1319 | 9.69 | NEG | 2 |  |  |  |  |  |  |  |  |  |  |  |  |  |  |  |  |  |  |  |  |  |  |  |  |  |  |  |
| **isorhamnetin** | C16 H12 O7 | 316.0583 | 5.19 | POS | 1 |  |  |  |  |  |  |  |  |  |  |  |  |  |  |  |  |  |  |  |  |  |  |  |  |  |  |  |
| **7-acetoxy-2-methyl-isoflavone, glyzarin** | C18 H14 O4 | 294.0892 | 6.86 | POS | 1 |  |  |  |  |  |  |  |  |  |  |  |  |  |  |  |  |  |  |  |  |  |  |  |  |  |  |  |
|  |  |  | 8.60 | POS | 2 |  |  |  |  |  |  |  |  |  |  |  |  |  |  |  |  |  |  |  |  |  |  |  |  |  |  |  |
| **formononetin** | C16 H12 O4 | 268.0736 | 8.72 | NEG | 2 |  |  |  |  |  |  |  |  |  |  |  |  |  |  |  |  |  |  |  |  |  |  |  |  |  |  |  |
|  |  |  | 8.97 | NEG | 2 |  |  |  |  |  |  |  |  |  |  |  |  |  |  |  |  |  |  |  |  |  |  |  |  |  |  |  |
| **licoisoflavone B** | C20 H16 O6 | 352.0947 | 9.10 | NEG | 2 |  |  |  |  |  |  |  |  |  |  |  |  |  |  |  |  |  |  |  |  |  |  |  |  |  |  |  |
|  |  |  | 4.85 | POS | 2 |  |  |  |  |  |  |  |  |  |  |  |  |  |  |  |  |  |  |  |  |  |  |  |  |  |  |  |
|  |  |  | 7.98 | NEG | 2 |  |  |  |  |  |  |  |  |  |  |  |  |  |  |  |  |  |  |  |  |  |  |  |  |  |  |  |
|  |  |  | 8.17 | NEG | 2 |  |  |  |  |  |  |  |  |  |  |  |  |  |  |  |  |  |  |  |  |  |  |  |  |  |  |  |
| **glabrene** | C20 H18 O4 | 322.1205 | 8.61 | NEG | 2 |  |  |  |  |  |  |  |  |  |  |  |  |  |  |  |  |  |  |  |  |  |  |  |  |  |  |  |
|  |  |  | 9.13 | NEG | 2 |  |  |  |  |  |  |  |  |  |  |  |  |  |  |  |  |  |  |  |  |  |  |  |  |  |  |  |
|  |  |  | 9.30 | NEG | 2 |  |  |  |  |  |  |  |  |  |  |  |  |  |  |  |  |  |  |  |  |  |  |  |  |  |  |  |
|  |  |  | 9.72 | NEG | 2 |  |  |  |  |  |  |  |  |  |  |  |  |  |  |  |  |  |  |  |  |  |  |  |  |  |  |  |
|  |  |  | 7.65 | NEG | 2 |  |  |  |  |  |  |  |  |  |  |  |  |  |  |  |  |  |  |  |  |  |  |  |  |  |  |  |
|  |  |  | 8.15 | NEG | 2 |  |  |  |  |  |  |  |  |  |  |  |  |  |  |  |  |  |  |  |  |  |  |  |  |  |  |  |
|  |  |  | 9.20 | NEG | 2 |  |  |  |  |  |  |  |  |  |  |  |  |  |  |  |  |  |  |  |  |  |  |  |  |  |  |  |
| **glabridin** | C20 H20 O4 | 324.1362 | 9.38 | NEG | 2 |  |  |  |  |  |  |  |  |  |  |  |  |  |  |  |  |  |  |  |  |  |  |  |  |  |  |  |
|  |  |  | 9.71 | NEG | 2 |  |  |  |  |  |  |  |  |  |  |  |  |  |  |  |  |  |  |  |  |  |  |  |  |  |  |  |
|  |  |  | 10.68 | POS | 2 |  |  |  |  |  |  |  |  |  |  |  |  |  |  |  |  |  |  |  |  |  |  |  |  |  |  |  |
|  |  |  | 11.15 | NEG | 2 |  |  |  |  |  |  |  |  |  |  |  |  |  |  |  |  |  |  |  |  |  |  |  |  |  |  |  |
| **glabrol** | C25 H28 O4 | 392.1988 | 8.75 | NEG | 2 |  |  |  |  |  |  |  |  |  |  |  |  |  |  |  |  |  |  |  |  |  |  |  |  |  |  |  |
|  |  |  | 9.19 | NEG | 2 |  |  |  |  |  |  |  |  |  |  |  |  |  |  |  |  |  |  |  |  |  |  |  |  |  |  |  |
|  |  |  | 4.96 | NEG | 2 |  |  |  |  |  |  |  |  |  |  |  |  |  |  |  |  |  |  |  |  |  |  |  |  |  |  |  |
| **licoflavonol, licoisoflavone A** | C20 H18 O6 | 354.1103 | 5.13 | NEG | 2 |  |  |  |  |  |  |  |  |  |  |  |  |  |  |  |  |  |  |  |  |  |  |  |  |  |  |  |
|  |  |  | 3.96 | POS | 2 |  |  |  |  |  |  |  |  |  |  |  |  |  |  |  |  |  |  |  |  |  |  |  |  |  |  |  |
| **licuraside** | C26 H30 O13 | 550.1686 | 4.12 | POS | 1 |  |  |  |  |  |  |  |  |  |  |  |  |  |  |  |  |  |  |  |  |  |  |  |  |  |  |  |
|  |  |  | 4.96 | POS | 2 |  |  |  |  |  |  |  |  |  |  |  |  |  |  |  |  |  |  |  |  |  |  |  |  |  |  |  |
| **isoliquiritigenin** | C15 H12 O4 | 256.0736 | 5.30 | POS | 2 |  |  |  |  |  |  |  |  |  |  |  |  |  |  |  |  |  |  |  |  |  |  |  |  |  |  |  |
|  |  |  | 4.12 | NEG | 2 |  |  |  |  |  |  |  |  |  |  |  |  |  |  |  |  |  |  |  |  |  |  |  |  |  |  |  |
| **martynoside, isomartynoside** | C31 H40 O15 | 652.2367 | 4.42 | NEG | 2 |  |  |  |  |  |  |  |  |  |  |  |  |  |  |  |  |  |  |  |  |  |  |  |  |  |  |  |
|  |  |  | 3.04 | POS | 2 |  |  |  |  |  |  |  |  |  |  |  |  |  |  |  |  |  |  |  |  |  |  |  |  |  |  |  |
| **verbascoside** | C29 H36 O15 | 624.2054 | 4.55 | POS | 2 |  |  |  |  |  |  |  |  |  |  |  |  |  |  |  |  |  |  |  |  |  |  |  |  |  |  |  |
|  |  |  | 5.20 | NEG | 2 |  |  |  |  |  |  |  |  |  |  |  |  |  |  |  |  |  |  |  |  |  |  |  |  |  |  |  |
| **syringin-4-O-beta-glucoside** | C23 H34 O14 | 534.1949 | 5.04 | POS | 2 |  |  |  |  |  |  |  |  |  |  |  |  |  |  |  |  |  |  |  |  |  |  |  |  |  |  |  |
| **salonitenolide** | C15 H20 O4 | 264.1362 | 5.20 | POS | 2 |  |  |  |  |  |  |  |  |  |  |  |  |  |  |  |  |  |  |  |  |  |  |  |  |  |  |  |
|  |  |  | 7.45 | POS | 2 |  |  |  |  |  |  |  |  |  |  |  |  |  |  |  |  |  |  |  |  |  |  |  |  |  |  |  |
| **cnicin** | C20 H26 O7 | 378.1679 | 15.31 | NEG | 2 |  |  |  |  |  |  |  |  |  |  |  |  |  |  |  |  |  |  |  |  |  |  |  |  |  |  |  |
|  |  |  | 10.71 | POS | 2 |  |  |  |  |  |  |  |  |  |  |  |  |  |  |  |  |  |  |  |  |  |  |  |  |  |  |  |
| **fenchone, citral** | C10 H16 O | 152.1201 | 11.63 | NEG | 1 |  |  |  |  |  |  |  |  |  |  |  |  |  |  |  |  |  |  |  |  |  |  |  |  |  |  |  |
| **alpha-amyrine, beta-amyrine, taraxerol, taraxasterol** | C30 H50 O | 426.3862 | 11.84 | NEG | 1 |  |  |  |  |  |  |  |  |  |  |  |  |  |  |  |  |  |  |  |  |  |  |  |  |  |  |  |
| **liquiritic acid, glycyrrhetinic acid** | C30 H46 O4 | 470.3396 | 13.71 | POS | 2 |  |  |  |  |  |  |  |  |  |  |  |  |  |  |  |  |  |  |  |  |  |  |  |  |  |  |  |
| **ursolic acid** | C30 H48 O3 | 456.3603 | 8.09 | NEG | 2 |  |  |  |  |  |  |  |  |  |  |  |  |  |  |  |  |  |  |  |  |  |  |  |  |  |  |  |
|  |  |  | 8.40 | NEG | 2 |  |  |  |  |  |  |  |  |  |  |  |  |  |  |  |  |  |  |  |  |  |  |  |  |  |  |  |
| **glabrolide, isoglabrolide** | C30 H44 O4 | 468.3240 | 3.48 | NEG | 2 |  |  |  |  |  |  |  |  |  |  |  |  |  |  |  |  |  |  |  |  |  |  |  |  |  |  |  |
| **glycyrrhizin** | C42 H62 O16 | 822.4038 | 4.85 | POS | 2 |  |  |  |  |  |  |  |  |  |  |  |  |  |  |  |  |  |  |  |  |  |  |  |  |  |  |  |
|  |  |  |  |  |  |  |  |  |  |  |  |  |  |  |  |  |  |  |  |  |  |  |  |  |  |  |  |  |  |  |  |  |
| **methylcoumarin** | C10 H8 O2 | 160.0524 |  |  |  |  |  |  |  |  |  |  |  |  |  |  |  |  |  |  |  |  |  |  |  |  |  |  |  |  |  |  |
|  |  |  |  |  |  |  |  |  |  |  |  |  |  |  |  |  |  |  |  |  |  |  |  |  |  |  |  |  |  |  |  |  |

**Supplementary Table 3** Correlation coefficients (R^2^)^a^ of dependence of antioxidant activity of 26 dietary supplements on U-HPLC-HRMS/MS responses^b^ of non-silymarin bioactive compounds present in *Silybum marianum*.

|  |  |  | **Potential identity (compound from database)** | **CAA** | **ABTS** | **ORAC** | **DPPH** |
| --- | --- | --- | --- | --- | --- | --- | --- |
| Phenolics | Simple phenolics |  | 1,5-O- dicaffeoylquinic acid; 3,5-O- dicaffeoylquinic acid; 4,5-O-dicaffeoylquinic acid; 5-O-feruloylquinic acid; caffeic acid; coniferylaldehyd; coumaric acid; cynarin; dihydroconiferyl alcohol; dihydroxybenzoic acid; ethyl caffeate; ferulic acid; gallic acid; guaiacol; chlorogenic acid; mariamide A,B; methyl ferulate; salicylic acid; sinapinic acid; syringaldehyde; syringic acid; vanillic acid | 0.543^a^ | 0.374 | 0.118 | 0.453 |
|  | Flavonoids | flavone/flavonol aglycones | apigenin; apigenin-5,7-dimethyl ether; kaempferol; kumatakenin; luteolin; nepetin; patuletin; pectolinarigenin; quercetin; rhamentin | 0.122 | -0.189 | -0.642 | 0.472 |
|  |  | flavone/flavonol glycosides | apigenin-4,7-diglucoside; apigenin-7-O-glucoside; apigenin-7-O-glucuronide; astragalin; cynaroside; isokaempferide-7-rhamnoside; isoorientin; isovitexin; kaempferol-3-rutinoside; miquelianin; orientin; rutin; vitexin | 0.221 | -0.051 | -0.052 | 0.339 |
|  |  | flavonolignans | isosilandrin A,B; neosilyhermin A,B; silandrin A,B; silyamandin; silyhermin; silymonin | 0.277 | 0.420 | -0.246 | 0.446 |
|  |  | isoflavone | genistein | -0.380 | 0.045 | 0.740 | 0.165 |
|  | SUM of flavone/flavonol aglycones and glycosides |  |  | 0.201 | -0.139 | -0.409 | 0.465 |
|  | SUM of flavone/flavonol aglycones and glycosides and isoflavonoids |  |  | 0.204 | -0.139 | -0.411 | 0.466 |
|  | SUM of flavonoids |  |  | 0.363 | 0.045 | -0.518 | 0.599^a^ |
|  | SUM of phenolics |  |  | 0.647^a^ | 0.332 | -0.171 | 0.607^a^ |
| Alkaloids |  |  | 3- methylcarboxymethyl-indole-1-N-beta-D-glucopyranoside | 0.520^a^ | 0.232 | -0.445 | 0.232 |

^a^ Correlation coefficient confirms (α=0.05) that the results of antioxidant assay linearly depend on U-HPLC-HRMS/MS responses of non-silymarin antioxidants present in *Silybum marianum* (ABTS df=14, critical value=0.497; ORAC df=19, critical value=0.433; DPPH df=20, critical value=0.423; CAA df=15, critical value=0.482).

^b^ For the non-silymarin antioxidants, the analytical standards were not available, so we correlated the sum of areas of the peaks of U-HPLC-HRMS/MS chromatograms.

**Supplementary Table 4** Correlation coefficients (R^2^) of dependence of antioxidant activity of 26 dietary supplements on U-HPLC-HRMS/MS responses^b^ of non-silymarin bioactive compounds present in other plants - *Schisandra chinensis*, *Cordyceps sinensis,* *Scutellaria baicalensis*, *Cnicus benedictus*, *Foeniculum vulgare*, *Taraxacum officinale* and *Glycyrrhiza glabra*.

|  |  |  | **Potential identity (compound from database)** | **R^2^** | | | | | **critical value** | | | | | |
| --- | --- | --- | --- | --- | --- | --- | --- | --- | --- | --- | --- | --- | --- | --- |
|  |  |  |  | CAA | ABTS | ORAC | | DPPH | CAA | ABTS | | | ORAC | DPPH |
| Phenolics | Simple phenolics |  | cordycepic acid; isomartynoside; martynoside; naphthaquinone; rosmarinic acid; syringin-4-O-beta-glucoside; verbascoside | 0.194 | -0.745 | -0.546 | -0.593 | | 0.95 | | 0.95 | 0.95 | | 0.95 |
|  | Coumarins |  | methylcoumarin | -0.604 | -0.218 | -0.100 | -0.451 | | 0.497 | | 0.433 | 0.423 | | 0.482 |
|  | Lignans | lignans | angeloylgomisin H; angeloylgomisin P; angeloylgomisin Q; arctigenin; benzoylgomisin H; gomisin A,B,DF,G,J,K1,K2,K3,L1,L2,M1,M2,N; isoschisandrin; propinquanin F; schisandrin A,B,C; schisantherin A,C; tigloylgomisin P; trachelogenin | -0.375 | -0.302 | 0.019 | 0.030 | | 0.497 | | 0.433 | 0.423 | | 0.482 |
|  |  | lignan glycosides | nortracheloside | 0.963 | -0.995 | 0.998^a^ | 0.919 | | 0.997 | | 0.997 | 0.997 | | 0.997 |
|  | SUM of lignans and lignan glycosides | |  | -0.390 | -0.305 | 0.017 | 0.029 | | 0.497 | | 0.433 | 0.423 | | 0.482 |
|  | Flavonoids | flavones/flavonols | baicalein; glabrol; isorhamnetin; neobaicalein; oroxylin A; skullcapflavone I; wogonin | 0.048 | -0.029 | -0.351 | 0.240 | | 0.497 | | 0.433 | 0.433 | | 0.497 |
|  |  | isoflavonoids | 7-acetoxy-2-methyl-isoflavone; formononetin; glabrene; glabridin; glyzarin; licoflavonol; licoisoflavone A, B | 0.000 | -0.569 | -0.773 | -0.318 | | 0.95 | | 0.95 | 0.95 | | 0.95 |
|  |  | chalcones | isoliquiritigenin; licuraside | 0.707 | -0.697 | 0.409 | -0.997 | | 0.95 | | 0.95 | 0.95 | | 0.95 |
|  | SUM of flavonoids |  |  | 0.117 | -0.056 | -0.341 | 0.199 | | 0.497 | | 0.433 | 0.433 | | 0.497 |
|  | SUM of phenolics |  |  | 0.029 | -0.315 | -0.023 | 0.058 | | 0.497 | | 0.433 | 0.423 | | 0.482 |
| Saponins | triterpenoidal |  | glabrolide; glycyrrhizin; isoglabrolide | -0.194 | 0.735 | 0.554 | 0.585 | | 0.95 | | 0.95 | 0.95 | | 0.95 |
| Terpenes | mono |  | citral; fenchone | -0.983 | 0.983 | -0.989 | -0.949 | | 0.997 | | 0.997 | 0.997 | | 0.997 |
|  | sesqui |  | cnicin; salonitenolide | -0.454 | 0.102 | -0.138 | -0.568 | | 0.997 | | 0.997 | 0.997 | | 0.997 |
|  | tri |  | alpha,beta-amyrine; glycyrrhetinic acid; liquiritic acid; taraxasterol; taraxerol; ursolic acid | -0.572 | 0.822 | -0.097 | 0.964^a^ | | 0.95 | | 0.95 | 0.95 | | 0.950 |
|  | SUM of terpenes |  |  | -0.596 | -0.893 | -0.606 | -0.726 | | 0.754 | | 0.754 | 0.754 | | 0.754 |

^a^ Correlation coefficient confirms (α=0.05) that the results of antioxidant assay linearly depend on U-HPLC-HRMS/MS responses of non-silymarin antioxidants present in other plants - *Schisandra chinensis*, *Cordyceps sinensis,* *Scutellaria baicalensis*, *Cnicus benedictus*, *Foeniculum vulgare*, *Taraxacum officinale* and *Glycyrrhiza glabra.*

^b^ For the non-silymarin antioxidants, the analytical standards were not available, so we correlated the sum of areas of the peaks of U-HPLC-HRMS/MS chromatograms
