# Supplementary material for: Complex Evaluation of Antioxidant Capacity of Milk Thistle Dietary Supplements
Source: Antioxidants (Basel). 2019 Aug 18;8(8):317. doi: 10.3390/antiox8080317 (PMC6720444; doi:10.3390/antiox8080317)
Supplement: Supplementary file 1 [file antioxidants-08-00317-s001.zip › antioxidants-550286-Supplementary-proofback_04-send XML-01.pdf]

Article

Complex Evaluation of Antioxidant Capacity of Milk Thistle Dietary Supplements

Jitka Viktorova [1], Milena Stranska-Zachariasova [2,*], Marie Fenclova [2], Libor Vitek [3], Jana Hajslova [2], Vladimir Kren [4] and Tomas Ruml [1]

1 Department of Biochemistry and Microbiology, University of Chemistry and Technology Prague, Technická 5, 166 28 Prague, Czech Republic
2 Department of Food Analysis and Nutrition, University of Chemistry and Technology Prague, Technická 5, 166 28 Prague, Czech Republice
3 4th Department of Internal Medicine and Institute of Medical Biochemistry and Laboratory Diagnostics, 1st Faculty of Medicine, Charles University, Katerinska 32, 12000 Prague, Czech Republic
4 Laboratory of Biotransformation, Institute of Microbiology, Czech Academy of Sciences, Vídeňská 1083, 142 20 Prague, Czech Republice
* Correspondence: milena.stranska@vscht.cz

Supplementary Table 1 List of non-silymarin bioactive compounds reported in literature for *Silybum marianum* (SM)[1, 2, 3-10], *Schisandra chinensis* (SCH)[11], *Cordyceps sinensis* (CS)[12, 13], *Scutellaria baicalensis* (SB)[14, 15], *Cnicus benedictus* (CB)[16], *Foeniculum vulgare* (FV)[17, 18], *Taraxacum officinale* (TO)[19] and *Glycyrrhiza glabra* (GG)[20].

Compound name	Summary formula	Plant of origin
neusilychristin	$C_{25}H_{22}O_{10}$	*SM*
silyamandin	$C_{25}H_{22}O_{11}$	*SM*
isosilandrin A	$C_{25}H_{22}O_{9}$	*SM*
isosilandrin B	$C_{25}H_{22}O_{9}$	*SM*
neosilyhermin A	$C_{25}H_{22}O_{9}$	*SM*
neosilyhermin B	$C_{25}H_{22}O_{9}$	*SM*
silandrin A	$C_{25}H_{22}O_{9}$	*SM*
silandrin B	$C_{25}H_{22}O_{9}$	*SM*
silyhermin	$C_{25}H_{22}O_{9}$	*SM*
silymonin	$C_{25}H_{22}O_{9}$	*SM*
apigenin	$C_{15}H_{10}O_{5}$	*SM*
genistein	$C_{15}H_{10}O_{5}$	*SM*
luteolin	$C_{15}H_{10}O_{6}$	*SM*
kaempferol	$C_{15}H_{10}O_{6}$	*SM*
quercetin	$C_{15}H_{10}O_{7}$	*SM*

myricetin	$C_{15}H_{10}O_8$	*SM*
naringenin	$C_{15}H_{12}O_5$	*SM*
dihydrokaempferol (aromadendrin)	$C_{15}H_{12}O_6$	*SM*
eriodictyol	$C_{15}H_{12}O_6$	*SM*
catechin	$C_{15}H_{14}O_6$	*SM*
acacetin	$C_{16}H_{12}O_5$	*SM*
genkwanin	$C_{16}H_{12}O_5$	*SM*
hispidulin	$C_{16}H_{12}O_6$	*SM*
chrysoeriol	$C_{16}H_{12}O_6$	*SM*
kaempferol-3-methyl ether (isokaempferide)	$C_{16}H_{12}O_6$	*SM*
nepetin	$C_{16}H_{12}O_7$	*SM*
rhamentin	$C_{16}H_{12}O_7$	*SM*
patuletin	$C_{16}H_{12}O_8$	*SM*
apigenin-5,7-dimethyl ether	$C_{17}H_{14}O_5$	*SM*
pectolinarigenin	$C_{17}H_{14}O_6$	*SM*
kumatakenin	$C_{17}H_{14}O_6$	*SM*
jaceosidin	$C_{17}H_{14}O_7$	*SM*
eupatilin	$C_{18}H_{16}O_7$	*SM*
sudachitin	$C_{18}H_{16}O_8$	*SM*
hymenoxin	$C_{19}H_{18}O_8$	*SM*
quercetin-3-O-arabinoside	$C_{20}H_{18}O_{11}$	*SM*
luteolin-7-O-glucuronide	$C_{21}H_{17}O_{12}$	*SM*
apigenin-7-O-glucuronide	$C_{21}H_{18}O_{11}$	*SM*
miquelianin (quercetin 3-glucoronide)	$C_{21}H_{18}O_{13}$	*SM*
isovitexin	$C_{21}H_{20}O_{10}$	*SM*
apigenin-7-O-glucoside	$C_{21}H_{20}O_{10}$	*SM*
vitexin	$C_{21}H_{20}O_{10}$	*SM*
isoorientin	$C_{21}H_{20}O_{11}$	*SM*
orientin	$C_{21}H_{20}O_{11}$	*SM*
kaempferol-3-O-beta-D-glucoside (astragalin)	$C_{21}H_{20}O_{11}$	*SM*

luteolin-7-O-glucoside (cynaroside)	$C_{21}H_{20}O_{11}$	*SM*
quercetin 3-O-galactoside (hyperoside)	$C_{21}H_{20}O_{12}$	*SM*
quercetin 3-O-glucoside (isoquercetin)	$C_{21}H_{20}O_{12}$	*SM*
spiraeoside (quercetin 4-O-glucoside)	$C_{21}H_{20}O_{12}$	*SM*
naringenin 7-O-beta-D-glucopyranoside	$C_{21}H_{22}O_{10}$	*SM*
isokaempferide 7-rhamnoside	$C_{22}H_{22}O_{10}$	*SM*
isorhamnetin 3-O-glucoside	$C_{22}H_{22}O_{12}$	*SM*
apigenin-4,7-diglucoside	$C_{27}H_{30}O_{15}$	*SM*
kaempferol-3-rutinoside	$C_{27}H_{30}O_{15}$	*SM*
rutin	$C_{27}H_{30}O_{16}$	*SM*
naringin	$C_{27}H_{32}O_{14}$	*SM*
hydroxybenzoic acid (salicylic acid)	$C_{7}H_{6}O_{3}$	*SM*
beta-resorcylic acid (dihydroxybenzoic acid)	$C_{7}H_{6}O_{4}$	*SM*
gallic acid	$C_{7}H_{6}O_{5}$	*SM*
guaiacol	$C_{7}H_{8}O_{2}$	*SM*
vanillic acid	$C_{8}H_{8}O_{4}$	*SM*
syringaldehyde	$C_{9}H_{10}O_{4}$	*SM*
syringic acid	$C_{9}H_{10}O_{5}$	*SM*
coumaric acid	$C_{9}H_{8}O_{3}$	*SM*
caffeic acid	$C_{9}H_{8}O_{4}$	*SM*
coniferylaldehyd	$C_{10}H_{10}O_{3}$	*SM*
ferulic acid	$C_{10}H_{10}O_{4}$	*SM*
dihydroconiferyl alcohol	$C_{10}H_{14}O_{3}$	*SM*
ethyl caffeate	$C_{11}H_{12}O_{4}$	*SM*
methyl ferulate	$C_{11}H_{12}O_{4}$	*SM*
sinapinic acid	$C_{11}H_{12}O_{5}$	*SM*
ellagic acid	$C_{14}H_{6}O_{8}$	*SM*
3-O-caffeoylquinic acid (PA1) (chlorogenic acid)	$C_{16}H_{18}O_{9}$	*SM*
4-O-caffeoylquinic acid (PA3)	$C_{16}H_{18}O_{9}$	*SM*
5-O-feruloylquinic acid (PA4)	$C_{17}H_{20}O_{9}$	*SM*

1,5-O- dicaffeoylquinic acid (PA6)	$C_{25}H_{24}O_{12}$	*SM*
3,5-O- dicaffeoylquinic acid (PA5)	$C_{25}H_{24}O_{12}$	*SM*
4,5-O-dicaffeoylquinic acid (PA7)	$C_{25}H_{24}O_{12}$	*SM*
cynarin	$C_{25}H_{24}O_{12}$	*SM*
mariamide A	$C_{42}H_{46}N_4O_{10}$	*SM*
mariamide B	$C_{21}H_{24}N_2O_5$	*SM*
3- methylcarboxymethyl-indole-1-N-beta-D-glucopyranoside	$C_{16}H_{19}NO_7$	*SM*
angeloylgomisin H	$C_{28}H_{36}O_8$	*SCH*
angeloylgomisin P	$C_{28}H_{34}O_9$	*SCH*
angeloylgomisin Q	$C_{29}H_{38}O_9$	*SCH*
benzoylgomisin H	$C_{30}H_{34}O_8$	*SCH*
benzoylgomisin O	$C_{30}H_{32}O_8$	*SCH*
gomisin A	$C_{23}H_{28}O_7$	*SCH*
gomisin B	$C_{28}H_{34}O_9$	*SCH*
gomisin D	$C_{28}H_{34}O_{10}$	*SCH*
gomisin F	$C_{28}H_{34}O_9$	*SCH*
gomisin G	$C_{30}H_{32}O_9$	*SCH*
gomisin J	$C_{22}H_{28}O_6$	*SCH*
gomisin K1, K2, K3	$C_{23}H_{30}O_6$	*SCH*
gomisin M1, M2, L1, L2	$C_{22}H_{26}O_6$	*SCH*
gomisin N	$C_{23}H_{28}O_6$	*SCH*
isoschisandrin	$C_{24}H_{32}O_7$	*SCH*
propinquanin F	$C_{28}H_{36}O_8$	*SCH*
schisandrin	$C_{24}H_{32}O_7$	*SCH*
schisandrin A	$C_{24}H_{32}O_6$	*SCH*
schisandrin B	$C_{23}H_{28}O_6$	*SCH*
schisandrin C	$C_{22}H_{24}O_6$	*SCH*
schisantherin A	$C_{30}H_{32}O_9$	*SCH*
schisantherin C	$C_{28}H_{34}O_9$	*SCH*
tigloylgomisin P	$C_{28}H_{34}O_9$	*SCH*

adenosine	$C_{10}H_{13}N_5O_4$	*CS*
cordycedipeptide A	$C_9H_{14}N_3O_3$	*CS*
cordycepic acid	$C_6H_{14}O_6$	*CS*
cordycepin	$C_{10}H_{13}N_5O_3$	*CS*
cordypyridone A	$C_{16}H_{23}NO_3$	*CS*
cordypyridone B	$C_{16}H_{23}NO_3$	*CS*
dipicolinic acid	$C_7H_5NO_4$	*CS*
farinosone A	$C_{25}H_{27}NO_4$	*CS*
farinosone B	$C_{25}H_{26}NO_5$	*CS*
farinosone C	$C_{19}H_{25}NO_5$	*CS*
hypoxanthine	$C_5H_4N_4O$	*CS*
macrolides	$C_{10}H_{14}O_4$	*CS*
militarinone A	$C_{26}H_{37}NO_6$	*CS*
militarinone B	$C_{26}H_{33}NO_5$	*CS*
militarinone C	$C_{26}H_{33}NO_4$	*CS*
militarinone D	$C_{26}H_{31}NO_4$	*CS*
myriocin	$C_{21}H_{39}NO_6$	*CS*
N-acetylgalactosamine	$C_8H_{15}NO_6$	*CS*
naphthaquinone	$C_{10}H_6O_2$	*CS*
paecilomycine A	$C_{15}H_{22}O_4$	*CS*
paecilomycine B	$C_{15}H_{22}O_5$	*CS*
paecilomycine C	$C_{15}H_{20}O_4$	*CS*
paecilosetin	$C_{22}H_{31}NO_4$	*CS*
spirotenuipesine A	$C_{15}H_{22}O_4$	*CS*
spirotenuipesine B	$C_{15}H_{22}O_5$	*CS*
2-(4-hydroxy phenyl) ethyl-O-beta-D- glucopyranoside	$C_{14}H_{20}O_7$	*SB*
apigenin 7-O-glucoside	$C_{21}H_{20}O_{10}$	*SB*
baicalein	$C_{15}H_{10}O_5$	*SB*
baicalin	$C_{21}H_{18}O_{11}$	*SB*
caffeic acid	$C_9H_8O_4$	*SB*

isomartynoside	$C_{31}H_{40}O_{15}$	*SB*
martynoside	$C_{31}H_{40}O_{15}$	*SB*
neobaicalein (skullcapflavone II)	$C_{19}H_{18}O_{8}$	*SB*
oroxylin A	$C_{16}H_{12}O_{5}$	*SB*
oroxylin A glucoronide	$C_{22}H_{20}O_{11}$	*SB*
skullcapflavone I	$C_{17}H_{14}O_{6}$	*SB*
skullcapflavone I 2'-O-glucoside	$C_{23}H_{24}O_{11}$	*SB*
ursolic acid	$C_{30}H_{48}O_{3}$	*SB*
verbascoside	$C_{29}H_{36}O_{15}$	*SB*
wogonin	$C_{16}H_{12}O_{5}$	*SB*
wogonoside	$C_{22}H_{20}O_{11}$	*SB*
cinnamaldehyde	$C_{9}H_{8}O$	*CB*
fenchone	$C_{10}H_{16}O$	*CB*
citral	$C_{10}H_{16}O$	*CB*
salonitenolide	$C_{15}H_{20}O_{4}$	*CB*
cnicin	$C_{20}H_{26}O_{7}$	*CB*
arctigenin	$C_{21}H_{24}O_{6}$	*CB*
trachelogenin	$C_{21}H_{24}O_{7}$	*CB*
nortracheloside	$C_{26}H_{32}O_{12}$	*CB*
absinthin	$C_{30}H_{40}O_{6}$	*CB*
alpha-amyrenone	$C_{30}H_{48}O$	*CB*
alpha-amyrine	$C_{30}H_{50}O$	*CB*
alpha-amyrin acetate	$C_{32}H_{52}O_{2}$	*CB*
multiflorenol acetate	$C_{32}H_{52}O_{2}$	*CB*
p-anisaldehyde	$C_{8}H_{8}O_{2}$	*FV*
trans-anethole	$C_{10}H_{12}O$	*FV*
estragole	$C_{10}H_{12}O$	*FV*
fenchone	$C_{10}H_{16}O$	*FV*
kaempferol	$C_{15}H_{10}O_{6}$	*FV*
naringenin	$C_{15}H_{12}O_{5}$	*FV*

acacetin	$C_{16}H_{12}O_5$	*FV*
isorhamnetin	$C_{16}H_{12}O_7$	*FV*
photoanethole	$C_{16}H_{16}O_2$	*FV*
3-O-caffeoylquinic acid	$C_{16}H_{18}O_9$	*FV*
4-O-caffeoylquinic acid	$C_{16}H_{18}O_9$	*FV*
5-O-caffeoylquinic acid	$C_{16}H_{18}O_9$	*FV*
sinapyl glucoside	$C_{17}H_{22}O_9$	*FV*
rosmarinic acid	$C_{18}H_{16}O_8$	*FV*
dianethole	$C_{19}H_{22}O_2$	*FV*
trans-resveratrol-3-O-beta-d-glucopyranoside	$C_{20}H_{22}O_8$	*FV*
kampferol-3-O-glucuronide	$C_{21}H_{18}O_{12}$	*FV*
quercetin-3-O-glucuronide	$C_{21}H_{18}O_{13}$	*FV*
kaempferol-3-O-glucoside	$C_{21}H_{20}O_{11}$	*FV*
quercetin-3-O-galactoside (hyperoside)	$C_{21}H_{20}O_{12}$	*FV*
isoquercetin	$C_{21}H_{20}O_{12}$	*FV*
isorhamnetin-3-O-glucoside	$C_{22}H_{22}O_{12}$	*FV*
syringin-4-O-beta-glucoside	$C_{23}H_{34}O_{14}$	*FV*
1,3-O-di-caffeoylquinic acid	$C_{25}H_{24}O_{12}$	*FV*
1,4-O-di-caffeoylquinic acid	$C_{25}H_{24}O_{12}$	*FV*
1,5-O-di-caffeoylquinic acid	$C_{25}H_{24}O_{12}$	*FV*
kaempferol-3-O-rutinoside	$C_{27}H_{30}O_{15}$	*FV*
eriodictyol-7-rutinoside	$C_{27}H_{32}O_{15}$	*FV*
cis a trans-miyabenol C	$C_{42}H_{32}O_9$	*FV*
gallic acid	$C_7H_6O_5$	*TO*
esculetin	$C_9H_6O_4$	*TO*
caffeic acid	$C_9H_8O_4$	*TO*
apigenin	$C_{15}H_{10}O_5$	*TO*
luteolin	$C_{15}H_{10}O_6$	*TO*
quercetin	$C_{15}H_{10}O_7$	*TO*
taraxinic acid	$C_{15}H_{18}O_4$	*TO*

coumestrol	$C_{15}H_8O_5$	*TO*
chlorogenic acid	$C_{16}H_{18}O_9$	*TO*
artemetin	$C_{20}H_{20}O_8$	*TO*
luteolin-7-O-beta-D-glucopyranoside (cyranoside)	$C_{21}H_{20}O_{11}$	*TO*
isoquercetin	$C_{21}H_{20}O_{12}$	*TO*
beta-amyrin	$C_{30}H_{50}O$	*TO*
taraxerol	$C_{30}H_{50}O$	*TO*
taraxasterol	$C_{30}H_{50}O$	*TO*
taraxasteryl acetate	$C_{32}H_{52}O_2$	*TO*
lutein epoxide	$C_{40}H_{56}O_3$	*TO*
lutein	$C_{40}H_{56}O_2$	*TO*
4-methyl coumarin	$C_{10}H_8O_2$	*GG*
liqcoumarin	$C_{12}H_{10}O_4$	*GG*
quercetin	$C_{15}H_{10}O_7$	*GG*
liquiritigenin	$C_{15}H_{12}O_4$	*GG*
isoliquiritigenin	$C_{15}H_{12}O_4$	*GG*
glyzaglabrin	$C_{16}H_{10}O_6$	*GG*
formononetin	$C_{16}H_{12}O_4$	*GG*
7-methoxy-2-methylisoflavone	$C_{17}H_{14}O_3$	*GG*
7-acetoxy-2-methyl-isoflavone	$C_{18}H_{14}O_4$	*GG*
glyzarin	$C_{18}H_{14}O_4$	*GG*
licoisoflavone B	$C_{20}H_{16}O_6$	*GG*
glabrene	$C_{20}H_{18}O_4$	*GG*
licoflavonol	$C_{20}H_{18}O_6$	*GG*
licoisoflavone A	$C_{20}H_{18}O_6$	*GG*
glabridin	$C_{20}H_{20}O_4$	*GG*
quercetin-3-glucoside	$C_{21}H_{20}O_{12}$	*GG*
isoliquiritin	$C_{21}H_{22}O_9$	*GG*
liquiritoside (liquiritin)	$C_{21}H_{22}O_9$	*GG*
glabrol	$C_{25}H_{28}O_4$	*GG*

licuraside	$C_{26}H_{30}O_{13}$	*GG*
isoglabrolide	$C_{30}H_{44}O_4$	*GG*
glabrolide	$C_{30}H_{44}O_4$	*GG*
liquoric acid	$C_{30}H_{44}O_5$	*GG*
liquiritic acid	$C_{30}H_{46}O_4$	*GG*
glycyrrhetinic acid (enoxolone)	$C_{30}H_{46}O_4$	*GG*
glycyrrhizin	$C_{42}H_{62}O_{16}$	*GG*
licoagrone	$C_{45}H_{42}O_{10}$	*GG*

[1] Abenavoli, L.M.; Capasso, R.; Milic, N.; Capasso, F. Milk thistle in liver diseases: Past, present, future. Phytother. Res. 2010, 24, 1423–1432.

[2] Chambers, C.S.; Holečková, V.; Petrásková, L.; Biedermann, D.; Valentová, K.; Buchta, M.; Křen, V. The silymarin composition… and why does it matter??? Food Res. Int. 2017, 100, 339–353, doi:10.1016/j.foodres.2017.07.017.

[3] Andrzejewska, J.; Martinelli, T.; Sadowska, K. Silybum marianum: Non-medical exploitation of the species. Ann. Appl. Boil. 2015, 167, 285–297.

[4] Milić, N.; Milosević, N.; Suvajdzić, L.; Zarkov, M.; Abenavoli, L. New therapeutic potentials of milk thistle (Silybum marianum). Nat. Prod. Commun. 2013, 8, 1801–1810.

[5] Csupor, D.; Csorba, A.; Hohmann, J. Recent advances in the analysis of flavonolignans of Silybum marianum. J. Pharm. Biomed. Anal. 2016, 130, 301–317.

[6] de Oliveira, D.R.; Schaffer, L.F.; Busanello, A.; Barbosa, C.P.; Peroza, L.R.; de Freitas, C.M.; Krum, B.N.; Bressan, G.N.; Boligon, A.A.; Athayde, M.L.; et al. Silymarin has antioxidant potential and changes the activity of na+/k+-atpase and monoamine oxidase in vitro. Ind. Crop. Prod. 2015, 70, 347–355, doi:10.1016/j.indcrop.2015.03.060.

[7] Lucini, L.; Kane, D.; Pellizzoni, M.; Ferrari, A.; Trevisi, E.; Ruzickova, G.; Arslan, D.; Luigi, L. Phenolic profile and in vitro antioxidant power of different milk thistle [Silybum marianum (L.) Gaertn.] cultivars. Ind. Crop. Prod. 2016, 83, 11–16.

[8] Qin, N.-B.; Jia, C.-C.; Xu, J.; Li, D.-H.; Xu, F.-X.; Bai, J.; Li, Z.-L.; Hua, H.-M. New amides from seeds of Silybum marianum with potential antioxidant and antidiabetic activities. Fitoterapia 2017, 119, 83–89.

[9] Mhamdi, B.; Abbassi, F.; Smaoui, A.; Abdelly, C.; Marzouk, B. Fatty acids, essential oil and phenolics composition of Silybum marianum seeds and their antioxidant activities. Pak. J. Pharm. Sci. 2016, 29, 953–959.

[10] Uehara, A.; Nakata, M.; Kitajima, J.; Iwashina, T. Internal and external flavonoids from the leaves of Japanese Chrysanthemum species (Asteraceae). Biochem. Syst. Ecol. 2012, 41, 142–149.

[11] Lee, D.-K.; Yoon, M.H.; Kang, Y.P.; Yu, J.; Park, J.H.; Lee, J.; Kwon, S.W. Comparison of primary and secondary metabolites for suitability to discriminate the origins of Schisandra chinensis by GC/MS and LC/MS. Food Chem. 2013, 141, 3931–3937.

[12] Tuli, H.S.; Sandhu, S.S.; Sharma, A.K. Pharmacological and therapeutic potential of cordyceps with special reference to cordycepin. 3 Biotech 2014, 4, 1–12, doi:10.1007/s13205-013-0121-9.

[13] Xiao, J.-H.; Zhong, J.-J. Secondary Metabolites from Cordyceps Species and Their Antitumor Activity Studies. Recent Patents Biotechnol. 2007, 1, 123–137.

[14] Mousavi, S.N.M.; Delazar, A.; Nazemiyeh, H.; Khodaie, L. Biological Activity and Phytochemical Study of Scutellaria platystegia. Iran. J. Pharm. Res. 2015, 14, 215–223.

[15] Gong, P.; Li, Y.; Yao, C.; Guo, H.; Hwang, H.; Liu, X.; Xu, Y.; Wang, X. Traditional Chinese Medicine on the Treatment of Coronary Heart Disease in Recent 20 Years. J. Altern. Complement. Med. 2017, 23, 659–666.

[16] Chabane, D.; Assani, A.; Mouhoub, F.; Chahinez, B.; Nacer-bey, N. Anatomical, phytochemical and pharmacological studies of roots of Cnicus benedictus L. Int. J. Med. Plant Res. 2013, 2, 204–208.

[17] Singh, G.; Maurya, S.; De Lampasona, M.P.; Catalán, C. Chemical constituents, antifungal and antioxidative potential of Foeniculum vulgare volatile oil and its acetone extract. Food Control. 2006, 17, 745–752.

[18] Rather, M.A.; Dar, B.A.; Sofi, S.N.; Bhat, B.A.; Qurishi, M.A. Foeniculum vulgare: A comprehensive review of its traditional use, phytochemistry, pharmacology, and safety. Arab. J. Chem. 2016, 9, S1574–S1583.

[19] Huber, M.; Triebwasser-Freese, D.; Reichelt, M.; Heiling, S.; Paetz, C.; Chandran, J.N.; Bartram, S.; Schneider, B.; Gershenzon, J.; Erb, M. Identification, quantification, spatiotemporal distribution and genetic variation of major latex secondary metabolites in the common dandelion (Taraxacum officinale agg.). Phytochem 2015, 115, 89–98.

[20] Fenwick, G.; Lutomski, J.; Nieman, C. Liquorice, Glycyrrhiza glabra L.—Composition, uses and analysis. Food Chem. 1990, 38, 119–143.

Supplementary Table 2 Characteristics of the bioactive compounds identified by the U-HPLC-HRMS/MS targeted screening.

Searched compound[a]	Elemental composition	Neutral exact mass (m/z)	RT (min)	Ionization polarity	Degree of certainty of compound identification[b]	Overview of the samples in which the compounds were detected[c]																										
						Silymarin SA	1	2	3	4	5	6	7	8	9	10	11	12	13	14	15	16	17	18	19	20	21	22	23	24	25	26
**isosilandrin A,B; neosilyhermin A,B; silandrin A,B; silyhermin, silymonin**	C25H22O9	466.1264	3.42	POS	2																											
			3.72	POS	2																											
			3.82	POS	2																											
			4.45	POS	2																											
			5.13	NEG	2																											
			5.85	NEG	2																											
			6.47	NEG	2																											
			6.57	NEG	2																											
			6.69	NEG	2																											
			6.94	NEG	2																											
**silyamandin**	C25H22O11	498.1162	3.80	NEG	2																											
			4.80	POS	2																											
			5.16	NEG	2																											
**catechin, epicatechin**	C15H14O6	290.0790	3.28	NEG	1																											
			3.77	NEG	2																											
			4.79	NEG	2																											
			5.60	NEG	2																											
**naringenin**	C15H12O5	272.0685	3.77	NEG	2																											
			4.59	NEG	2																											
			5.14	POS	2																											
			5.88	NEG	1																											
			6.09	NEG	2																											
			6.58	NEG	2																											
			7.98	NEG	2																											
**aromadendrin, eriodictyol**	C15H12O6	288.0634	4.55	NEG	2																											
			5.28	NEG	2																											
			5.65	POS	2																											
**eupatilin**	C18H16O7	344.0896	4.18	NEG	2																											
			4.92	NEG	2																											
			6.46	NEG	2																											
			6.55	POS	2																											
			6.71	POS	2																											

			7.75	POS	2
			8.03	POS	2
**hymenoxin**	C19H18O8	374.1002	6.74	POS	2
			6.96	POS	2
**jaceosidin**	C17H14O7	330.0740	4.26	NEG	2
			5.32	POS	2
			5.45	NEG	2
			6.07	POS	2
			6.19	NEG	2
			6.42	POS	2
			6.67	POS	2
			6.88	NEG	2
**sudachitin**	C18H16O8	360.0845	4.18	NEG	1
			5.66	POS	2
			5.75	NEG	2
**vitexin, isovitexin, apigenin-7-O-glucoside**	C21H20O10	432.1056	4.13	POS	2
			6.41	NEG	2
**luteolin**	C15H10O6	286.0477	4.01	NEG	1
			5.78	NEG	1
**kaempferol**	C15H10O6	286.0477	5.26	POS	2
			6.34	NEG	2
			6.88	NEG	2
**pectolinarigenin, kumatakenin**	C17H14O6	314.0790	3.69	NEG	2
			5.27	NEG	2
			5.75	NEG	2
			6.71	POS	2
			7.10	NEG	2
			7.23	POS	2
			7.55	NEG	2
			5.17	POS	2
**apigenin**	C15H10O5	270.0528	6.35	POS	1
**genistein**	C15H10O5	270.0528	5.63	NEG	2
			8.14	NEG	2

			3.78	NEG	2
**nepetin, rhamentin**	C16H12O7	316.0583	4.00	NEG	2
			4.27	NEG	2
			4.80	POS	2
			5.63	NEG	2
			6.34	NEG	2
			6.63	NEG	2
			6.74	POS	2
			2.98	POS	2
			3.88	NEG	2
			4.40	NEG	2
**orientin, isoorientin, astragalin, cynaroside**	C21H20O11	448.1006	3.93	NEG	2
			4.02	POS	2
			4.92	NEG	2
**apigenin-5,7-dimethyl ether**	C17H14O5	298.0841	5.41	NEG	2
			5.73	POS	2
			6.09	POS	2
			6.50	POS	2
			6.70	NEG	2
			7.76	NEG	2
			5.18	POS	2
			5.59	POS	2
			6.00	POS	2
**apigenin-7-O-glucuronide**	C21H18O11	446.0849	5.78	POS	2
			4.26	NEG	1
			4.67	NEG	2
**isokaempferide-7-rhamnoside**	C22H22O10	446.1213	8.79	POS	2
**rutin**	C27H30O16	610.1534	4.21	NEG	2
	C27H30O15	594.1585	4.69	NEG	2

apigenin-4,7-diglucoside, kaempferol-3-rutinoside			5.96	NEG	2
miquelianin	C21H18O13	478.0747	6.79	POS	2
patuletin	C16H12O8	332.0532	3.14	POS	2
			3.69	NEG	1
			3.75	NEG	2
quercetin	C15H10O7	302.0427	3.99	POS	1
			4.30	POS	2
			5.61	NEG	2
			5.93	NEG	1
			6.81	NEG	2
			1.80	POS	2
			2.88	POS	2
			2.96	POS	2
coniferylaldehyd	C10H10O3	178.0630	3.56	NEG	2
			4.30	POS	2
			4.87	POS	2
			3.21	NEG	2
			3.56	NEG	2
			4.52	POS	2
dihydroconiferyl alcohol	C10H14O3	182.0943	1.67	NEG	2
			3.03	NEG	2
			3.20	NEG	2
syringaldehyde	C9H10O4	182.0579	3.79	POS	2
			5.01	NEG	2
			5.95	POS	2
			10.20	POS	2
			1.65	POS	2
			3.25	NEG	2
			3.46	NEG	2
ethyl caffeate, methyl ferulate	C11H12O4	208.0736	3.97	NEG	2
			4.22	POS	2
			4.87	NEG	2

			5.30	NEG	2
			5.71	NEG	2
			5.89	POS	2
			5.97	NEG	2
			7.09	POS	2
			4.27	NEG	2
			4.59	NEG	2
			1.65	NEG	2
**1,5-O-dicaffeoylquinic acid, 3,5-O-dicaffeoylquinic acid, 4,5-O-dicaffeoylquinic acid, cynarin**	C25H24O12	516.1268	3.19	NEG	2
			3.61	NEG	2
**5-O-feruloylquinic acid**	C17H20O9	368.1107	3.29	NEG	1
			3.36	POS	2
			4.95	POS	2
**caffeic acid**	C9H8O4	180.0423	8.69	POS	2
			1.73	POS	2
			3.64	NEG	1
			6.94	NEG	2
**coumaric acid**	C9H8O3	164.0473	5.37	NEG	1
			3.57	NEG	1
			3.20	NEG	1
			3.11	POS	2
**dihydroxybenzoic acid**	C7H6O4	154.0266	3.24	NEG	2
			4.10	NEG	2
**ferulic acid**	C10H10O4	194.0579	4.94	NEG	2
			5.51	POS	2
			1.33	NEG	1
			2.37	POS	2
			2.98	POS	2
**gallic acid**	C7H6O5	170.0215	4.05	NEG	2
			4.74	POS	2

			2.99	NEG	2																											
			4.13	POS	2																											
			5.92	POS	2																											
**guaiacol**	C7H8O2	124.0524	1.88	NEG	2																											
			3.08	NEG	1																											
			3.88	NEG	2																											
**chlorogenic acid**	C16H18O9	354.0951	1.65	POS	2																											
			2.75	NEG	1																											
			3.00	NEG	2																											
**salicylic acid**	C7H6O3	138.0317	3.19	NEG	2																											
			3.90	NEG	2																											
			4.55	NEG	2																											
			3.56	NEG	2																											
			4.12	POS	2																											
			4.50	NEG	2																											
**sinapinic acid**	C11H12O5	224.0685	1.64	NEG	2																											
			2.86	POS	2																											
			4.03	NEG	2																											
**syringic acid**	C9H10O5	198.0528	1.48	POS	2																											
			1.65	NEG	1																											
			3.02	POS	2																											
**vanillic acid**	C8H8O4	168.0423	3.16	POS	2																											
			3.34	POS	2																											
			4.69	POS	2																											
			4.89	POS	2																											
			5.05	POS	2																											
**mariamide A**	C42H46N4O10	766.3214	5.24	POS	2																											
			5.36	POS	2																											
			5.45	POS	2																											
			5.69	POS	2																											
			6.04	POS	2																											
			14.82	POS	2																											
			4.32	POS	2																											
			4.65	POS	2																											

			5.00	POS	2
**mariamide B**	C21H24N2O5	384.1685	3.53	POS	2
			3.34	POS	2
			7.71	POS	2
**3-methylcarboxymethyl-indole-1-N-beta-D-glucopyranoside**	C16H19NO7	337.1162	8.00	POS	2
**naphthaquinone**	C10 H6 O2	158.0368	9.09	NEG	2
**angeloylgomisin H, propinquanin F**	C28 H36 O8	500.2410	7.97	POS	2
			8.15	POS	2
			8.36	POS	2
**angeloylgomisin P, gomisin B, gomisin F, schisantherin C, tigloylgomisin P**	C28 H34 O9	514.2203	8.46	POS	2
			8.59	POS	2
			8.79	POS	2
			9.11	POS	2
			7.86	POS	2
			8.06	POS	2
			7.71	POS	2
**angeloylgomisin Q**	C29 H38 O9	530.2516	8.00	POS	2
			7.76	POS	2
**benzoylgomisin H**	C30 H34 O8	522.2254	8.44	POS	2
			9.09	NEG	2
**gomisin A**	C23 H28 O7	416.1835	6.69	POS	2
			7.34	POS	2
			8.07	POS	2
**gomisin D**	C28 H34 O10	530.2152	8.64	POS	2
			7.90	POS	2
**gomisin G, schisantherin A**	C30 H32 O9	536.2046	8.27	POS	2
			8.37	NEG	2
**gomisin J**	C22 H28 O6	388.1886	8.53	POS	2

			8.81	POS	2	
			8.98	POS	2	
			9.19	POS	2	
**gomisin K1, K2, K3**	C23 H30 O6	402.2042	9.26	POS	2	
			9.46	POS	2	
**gomisin M1, M2, L1, L2**	C22 H26 O6	386.1729	9.57	POS	2	
			8.01	POS	2	
			8.20	POS	2	
			10.17	POS	2	
**gomisin N, schisandrin B**	C23 H28 O6	400.1886	10.27	POS	2	
			9.52	POS	2	
			9.11	NEG	2	
			10.85	POS	2	
**schisandrin A**	C24 H32 O6	416.2199	6.67	POS	2	
**schisandrin C**	C22 H24 O6	384.1573	7.06	POS	2	
			7.76	POS	2	
**schisandrin, isoschisandrin**	C24 H32 O7	432.2148	5.62	POS	2	
			5.95	POS	2	
			6.31	POS	2	
**trachelogenin**	C21 H24 O7	388.1522	5.84	POS	2	
			4.10	POS	2	
			3.96	POS	2	
**arctigenin**	C21 H24 O6	372.1573	4.18	NEG	2	
**nortracheloside**	C26 H32 O12	536.1894	5.74	POS	2	
**rosmarinic acid**	C18 H16 O8	360.0845	5.98	POS	2	
			7.10	NEG	2	
			0.73	NEG	2	
			5.19	POS	1	
			5.40	POS	2	
**cordycepic acid**	C6 H14 O6	182.0790	5.63	NEG	2	
**baicalein**	C15 H10 O5	270.0528	6.39	NEG	2	
			6.73	POS	2	

			6.96	POS	2
			5.37	NEG	2
**neobaicalein**	C19 H18 O8	374.1002	5.75	NEG	2
			5.97	NEG	2
**skullcapflavone I**	C17 H14 O6	314.0790	7.10	NEG	2
			7.23	POS	2
			7.55	NEG	2
			5.41	POS	2
			7.38	POS	2
			7.57	POS	2
**oroxylin A, wogonin**	C16 H12 O5	284.0685	5.22	NEG	1
			5.58	NEG	1
			5.86	POS	2
**baicalin**	C21 H18 O11	446.0849	5.93	NEG	2
			5.68	POS	2
			5.86	POS	2
			6.13	POS	2
**oroxylin A glucoronide, wogonoside**	C22 H20 O11	460.1006	6.70	POS	1
			9.12	NEG	2
**skullcapflavone I 2-O-glucoside**	C23 H24 O11	476.1319	9.69	NEG	2
**isorhamnetin**	C16 H12 O7	316.0583	5.19	POS	1
**7-acetoxy-2-methyl-isoflavone, glyzarin**	C18 H14 O4	294.0892	6.86	POS	1
			8.60	POS	2
**formononetin**	C16 H12 O4	268.0736	8.72	NEG	2
			8.97	NEG	2
**licoisoflavone B**	C20 H16 O6	352.0947	9.10	NEG	2
			4.85	POS	2
			7.98	NEG	2
			8.17	NEG	2
**glabrene**	C20 H18 O4	322.1205	8.61	NEG	2
			9.13	NEG	2

			9.30	NEG	2
			9.72	NEG	2
			7.65	NEG	2
			8.15	NEG	2
			9.20	NEG	2
**glabridin**	C20 H20 O4	324.1362	9.38	NEG	2
			9.71	NEG	2
			10.68	POS	2
			11.15	NEG	2
**glabrol**	C25 H28 O4	392.1988	8.75	NEG	2
			9.19	NEG	2
			4.96	NEG	2
**licoflavonol, licoisoflavone A**	C20 H18 O6	354.1103	5.13	NEG	2
			3.96	POS	2
**licuraside**	C26 H30 O13	550.1686	4.12	POS	1
			4.96	POS	2
**isoliquiritigenin**	C15 H12 O4	256.0736	5.30	POS	2
			4.12	NEG	2
**martynoside, isomartynoside**	C31 H40 O15	652.2367	4.42	NEG	2
			3.04	POS	2
**verbascoside**	C29 H36 O15	624.2054	4.55	POS	2
			5.20	NEG	2
**syringin-4-O-beta-glucoside**	C23 H34 O14	534.1949	5.04	POS	2
**salonitenolide**	C15 H20 O4	264.1362	5.20	POS	2
			7.45	POS	2
**cnicin**	C20 H26 O7	378.1679	15.31	NEG	2
			10.71	POS	2
**fenchone, citral**	C10 H16 O	152.1201	11.63	NEG	1

**alpha-amyrine, beta-amyrine, taraxerol, taraxasterol**	C30 H50 O	426.3862	11.84	NEG	1
**liquiritic acid, glycyrrhetinic acid**	C30 H46 O4	470.3396	13.71	POS	2
**ursolic acid**	C30 H48 O3	456.3603	8.09	NEG	2
			8.40	NEG	2
**glabrolide, isoglabrolide**	C30 H44 O4	468.3240	3.48	NEG	2
**glycyrrhizin**	C42 H62 O16	822.4038	4.85	POS	2
**methylcoumarin**	C10 H8 O2	160.0524			

Supplementary Table 3 Correlation coefficients (R^2)[a] of dependence of antioxidant activity of 26 dietary supplements on U-HPLC-HRMS/MS responses[b] of non-silymarin bioactive compounds present in *Silybum marianum*.

			**Potential identity (compound from database)**	**CAA**	**ABTS**	**ORAC**	**DPPH**
Phenolics	Simple phenolics		1,5-O- dicaffeoylquinic acid; 3,5-O-dicaffeoylquinic acid; 4,5-O-dicaffeoylquinic acid; 5-O-feruloylquinic acid; caffeic acid; coniferylaldehyd; coumaric acid; cynarin; dihydroconiferyl alcohol; dihydroxybenzoic acid; ethyl caffeate; ferulic acid; gallic acid; guaiacol; chlorogenic acid; mariamide A,B; methyl ferulate; salicylic acid; sinapinic acid; syringaldehyde; syringic acid; vanillic acid	0.543[a]	0.374	0.118	0.453
	Flavonoids	flavone/flavonol aglycones	apigenin; apigenin-5,7-dimethyl ether; kaempferol; kumatakenin; luteolin; nepetin; patuletin; pectolinarigenin; quercetin; rhamentin	0.122	-0.189	-0.642	0.472
		flavone/flavonol glycosides	apigenin-4,7-diglucoside; apigenin-7-O-glucoside; apigenin-7-O-glucuronide; astragalin; cynaroside;	0.221	-0.051	-0.052	0.339

			isokaempferide-7-rhamnoside; isoorientin; isovitexin; kaempferol-3-rutinoside; miquelianin; orientin; rutin; vitexin				
		flavonolignans	isosilandrin A,B; neosilyhermin A,B; silandrin A,B; silyamandin; silyhermin; silymonin	0.277	0.420	-0.246	0.446
		isoflavone	genistein	-0.380	0.045	0.740	0.165
	SUM of flavone/flavonol aglycones and glycosides			0.201	-0.139	-0.409	0.465
	SUM of flavone/flavonol aglycones and glycosides and isoflavonoids			0.204	-0.139	-0.411	0.466
	SUM of flavonoids			0.363	0.045	-0.518	0.599[a]
	SUM of phenolics			0.647[a]	0.332	-0.171	0.607[a]
Alkaloids			3- methylcarboxymethyl-indole-1-N-beta-D-glucopyranoside	0.520[a]	0.232	-0.445	0.232

[a] Correlation coefficient confirms (α=0.05) that the results of antioxidant assay linearly depend on U-HPLC-HRMS/MS responses of non-silymarin antioxidants present in *Silybum marianum* (ABTS df=14, critical value=0.497; ORAC df=19, critical value=0.433; DPPH df=20, critical value=0.423; CAA df=15, critical value=0.482).

[b] For the non-silymarin antioxidants, the analytical standards were not available, so we correlated the sum of areas of the peaks of U-HPLC-HRMS/MS chromatograms.

Supplementary Table 4 Correlation coefficients (R^2) of dependence of antioxidant activity of 26 dietary supplements on U-HPLC-HRMS/MS responses[b] of non-silymarin bioactive compounds present in other plants - *Schisandra chinensis, Cordyceps sinensis, Scutellaria baicalensis, Cnicus benedictus, Foeniculum vulgare, Taraxacum officinale* and *Glycyrrhiza glabra*.

			**Potential identity (compound from database)**	**R^2**				**critical value**			
				CAA	ABTS	ORAC	DPPH	CAA	ABTS	ORAC	DPPH
Phenolics	Simple phenolics		cordycepic acid; isomartynoside; martynoside; naphthaquinone; rosmarinic acid; syringin-4-O-beta-glucoside; verbascoside	0.194	-0.745	-0.546	-0.593	0.95	0.95	0.95	0.95
	Coumarins		methylcoumarin	-0.604	-0.218	-0.100	-0.451	0.497	0.433	0.423	0.482
	Lignans	lignans	angeloylgomisin H; angeloylgomisin P; angeloylgomisin Q; arctigenin; benzoylgomisin H; gomisin A,B,DF,G,J,K1,K2,K3,L1,L2,M1,M2,N; isoschisandrin; propinquanin F; schisandrin A,B,C; schisantherin A,C; tigloylgomisin P; trachelogenin	-0.375	-0.302	0.019	0.030	0.497	0.433	0.423	0.482
		lignan glycosides	nortracheloside	0.963	-0.995	0.998[a]	0.919	0.997	0.997	0.997	0.997
	SUM of lignans and lignan glycosides			-0.390	-0.305	0.017	0.029	0.497	0.433	0.423	0.482
	Flavonoids	flavones/flavonols	baicalein; glabrol; isorhamnetin; neobaicalein; oroxylin A; skullcapflavone I; wogonin	0.048	-0.029	-0.351	0.240	0.497	0.433	0.433	0.497
		isoflavonoids	7-acetoxy-2-methyl-isoflavone; formononetin; glabrene; glabridin; glyzarin; licoflavonol; licoisoflavone A, B	0.000	-0.569	-0.773	-0.318	0.95	0.95	0.95	0.95
		chalcones	isoliquiritigenin; licuraside	0.707	-0.697	0.409	-0.997	0.95	0.95	0.95	0.95
	SUM of flavonoids			0.117	-0.056	-0.341	0.199	0.497	0.433	0.433	0.497
	SUM of phenolics			0.029	-0.315	-0.023	0.058	0.497	0.433	0.423	0.482
Saponins	triterpenoidal		glabrolide; glycyrrhizin; isoglabrolide	-0.194	0.735	0.554	0.585	0.95	0.95	0.95	0.95
Terpenes	mono		citral; fenchone	-0.983	0.983	-0.989	-0.949	0.997	0.997	0.997	0.997
	sesqui		cnicin; salonitenolide	-0.454	0.102	-0.138	-0.568	0.997	0.997	0.997	0.997
	tri		alpha,beta-amyrine; glycyrrhetinic acid; liquiritic acid; taraxasterol; taraxerol; ursolic acid	-0.572	0.822	-0.097	0.964[a]	0.95	0.95	0.95	0.950

SUM of terpenes	-0.596	-0.893	-0.606	-0.726	0.754	0.754	0.754	0.754

[a] Correlation coefficient confirms (α=0.05) that the results of antioxidant assay linearly depend on U-HPLC-HRMS/MS responses of non-silymarin antioxidants present in other plants - *Schisandra chinensis*, *Cordyceps sinensis*, *Scutellaria baicalensis*, *Cnicus benedictus*, *Foeniculum vulgare*, *Taraxacum officinale* and *Glycyrrhiza glabra.*

[b] For the non-silymarin antioxidants, the analytical standards were not available, so we correlated the sum of areas of the peaks of U-HPLC-HRMS/MS chromatograms
